# Sequence- and Structure-Based Analysis of Tissue-Specific Phosphorylation Sites

**DOI:** 10.1371/journal.pone.0157896

**Published:** 2016-06-22

**Authors:** Nermin Pinar Karabulut, Dmitrij Frishman

**Affiliations:** 1 Department of Genome Oriented Bioinformatics, Technische Universität München, Freising, Germany; 2 Helmholtz Zentrum Munich; German Research Center for Environmental Health (GmbH), Institute of Bioinformatics and Systems Biology, Neuherberg, Germany; 3 St Petersburg State Polytechnical University, St Petersburg, Russia; Institut de Génétique et Développement de Rennes, FRANCE

## Abstract

Phosphorylation is the most widespread and well studied reversible posttranslational modification. Discovering tissue-specific preferences of phosphorylation sites is important as phosphorylation plays a role in regulating almost every cellular activity and disease state. Here we present a comprehensive analysis of global and tissue-specific sequence and structure properties of phosphorylation sites utilizing recent proteomics data. We identified tissue-specific motifs in both sequence and spatial environments of phosphorylation sites. Target site preferences of kinases across tissues indicate that, while many kinases mediate phosphorylation in all tissues, there are also kinases that exhibit more tissue-specific preferences which, notably, are not caused by tissue-specific kinase expression. We also demonstrate that many metabolic pathways are differentially regulated by phosphorylation in different tissues.

## Introduction

Protein phosphorylation is a reversible posttranslational modification (PTM) that represents the most common PTM type in eukaryotes and plays a crucial role in many essential cellular processes, including cellular signaling, metabolism, differentiation, regulation of protein activity and subcellular localization [1]. Protein phosphorylation and de-phosphorylation are controlled by more than 500 protein kinases and more than 100 phosphatases, respectively, which, in their turn, are regulated by phosphorylation, yielding a complex picture of interconnected signaling pathways. As many of these pathways are disease-related, understanding the mechanisms of phosphorylation has become a high priority for drug design.

Quantitative mass spectrometry-based phosphoproteomics has allowed for a comprehensive characterization of serine/threonine/tyrosine phosphorylation sites, enabling the analyses of molecular mechanisms behind phosphorylation. Multiple attempts have been made to derive consensus sequence motifs of phosphorylation sites [2, 3], analyze their structural properties [4, 5] as well as amino acid preferences in their spatial environments [6, 7], establish kinase-phosphorylation site associations [2, 8–10], and predict phosphorylated sites [11–13], their kinase-specificity [13–20], and subcellular distribution [21]. Many of these studies focused on the sequence and structural determinants of substrate-specificity across different kinase types.

Recent research indicates that many PTMs, including phosphorylation sites, are differential across tissues [22–24]. In our previous work we have shown that the sequence and spatial properties of acetylation sites vary from tissue to tissue and proposed that this diversity depends on differential usage of lysine acetyltranferases and lysine deacetylases [25]. A similar comprehensive analysis of sequence and structural preferences of tissue-specific phosphorylation sites has not been performed yet, although differential PTM sites across tissues have already been experimentally characterized.

Here we present a comprehensive sequence- and structure-based analysis of tissue-specific phosphorylation sites as well as global phosphorylation sites, employing a recent experimental dataset made available by Lundby *et al*. [23]. We examined differential biological pathways and domain preferences of phosphorylation sites across tissues. Our principal finding is that phosphorylation sites demonstrate tissue-specific preferences for certain residues in their sequence and spatial environments. We also identified kinases that are active in a tissue-specific manner, an effect which is apparently not caused by tissue-specific expression of kinase genes.

## Materials and Methods

### Datasets of Phosphorylated and Reference (Non-Phosphorylated) Sites

We used the dataset of 31480 phosphorylation sites in 7280 proteins identified by high-resolution tandem mass spectrometry in 14 rat tissues: brain (dissected into cerebellum, cortex and brainstem), heart, muscle, lung, kidney, liver, stomach, pancreas, spleen, thymus, intestine, testis, perirenal fat, and blood [23]. The UniProt [26] IDs of the best-matching proteins (one or more), the sequence position of the phosphorylation site, and the intensity values (summed up extracted ion current of all isotopic clusters associated with the peptide in the corresponding tissue) were gathered for each phosphorylation site in each tissue.

The best-matching UniProt ID for each phosphorylated peptide was identified as described previously [25]. Briefly, we aligned all pairs of proteins associated with a given peptide and chose the longer protein of the pair having the maximum sequence identity out of all pairs. We obtained 17917 phosphorylation sites in 5443 proteins, each of them having only one best-matching UniProt ID. This dataset contains 14661 phosphorylated serine sites (PSSs), 2832 phosphorylated threonine sites (PTSs), and 424 phosphorylated tyrosine sites (PYSs) (Table 1). The decrease in the number of phosphorylation sites may be due to failure in finding either the best-matching UniProt ID, or a serine (S)/threonine (T)/tyrosine (Y) residue in the specified sequence position, or the sequence of the corresponding full-length protein in the UniProt database.

**Table 1:** Data summary of phosphorylation sites.

Datasets	Number of proteins^a^	S^b^	T^c^	Y^d^	Total^e^
Initial dataset^f^	5443	14661/348754	2832/215735	424/100083	17917/664572
PS1D-70^g^	5286	9254/128578	1594/82698	249/38598	11097/249874
Structure-based dataset^h^	399	729/7377	232/6564	69/4384	1030/18325
Structure-based and solvent accessible dataset^i^	338	489/5941	181/5482	53/3981	723/15404
PS3D-90^j^	332	423/4162	140/3790	46/2804	609/10756

^a^ Number of serine phosphorylated proteins. See S1 Data for protein UniProt ids.

^b^ Number of serine phosphorylation sites/non-phosphorylation sites.

^c^ Number of threonine phosphorylation sites/non-phosphorylation sites.

^d^ Number of tyrosine phosphorylation sites/non-phosphorylation sites.

^e^ Total number of phosphorylation sites/non-phosphorylation sites including all residue types.

^f^ Initial dataset directly obtained from the study of Lundby *et al*. [23].

^g^ Sequence-based dataset after sequence redundancy reduction on peptides at the 70% identity level.

^h^ Structure-based dataset where phosphoproteins were mapped on PDB structures.

^i^ Structure-based dataset including solvent accessible phosphorylation/non-phosphorylation sites.

^j^ Structure-based dataset after the sequence redundancy reduction on peptides at the 90% identity level.

We also generated a negative (reference or non-PSSs, non-PTSs, non PYSs) dataset by extracting all S/T/Y residues not annotated as phosphorylated by Lundby *et al*. and matching them to those tissues in which the protein containing the reference site had at least one experimentally observed PSS/PTS/PYS. Then, the 21-mer sequences (from -10 to +10) surrounding each site in both positive and negative datasets were extracted and homology reduction on these 21-mers was performed using CD-HIT [27] at the 70% identity threshold. Some of the phosphorylation and reference sites are found in more than one tissue. The statistics of the resulting dataset, PS1D-70, can be found in Table 1 (11097 phosphorylation sites in 5286 proteins).

### Identification of Sequence Motifs

We used the Two Sample Logo method [28] to find enriched and depleted residues in the 21-mer sequences occurring in different tissues, using the associated PSSs/PTSs/PYSs and non-PSS/non-PTSs/non-PYSs as positive and negative sample inputs, respectively. For instance, PSSs/PTSs/PYSs found in kidney were compared to non-PSS/non-PTSs/non-PYSs in kidney. The Motif-X online tool [29] was employed to detect sequence motifs from 21-mer sequences where PSSs/PTSs/PYSs and non-PSS/non-PTSs/non-PYSs were used as the foreground and background datasets, respectively.

### Obtaining 3D Structures of Phosphorylated Proteins

We applied the same procedure as in our previous study [25] to collect 3D structures of phosphorylated proteins. We extracted the total of 2079 related 3D structures from the Protein Data Bank based on BLAST-P hits. After requiring at least 80% identity within the sequence segment spanning ±50 sequence positions around the phosphorylated residue, we obtained 1030 phosphorylation sites in 399 protein structures with the resolution better that 3Å.

Once the structure-based positive and negative PSSs/PTSs/PYSs datasets were generated as described above for the sequence-based data, homology reduction was again performed at the 90% identity level, taking into account only solvent accessible phosphorylation sites. The resulting dataset, which we call PS3D-90, contains 609 phosphorylation sites (423 PSSs, 140 PTSs and 46 PYSs) and 10756 non-phosphorylation sites (4162 PSSs, 3790 PTSs and 2804 PYSs) in 332 proteins with known structures (Table 1). The number of PSSs, PTSs and PYSs in each tissue can be found in Tables D, E and F in S1 Supporting Tables, respectively. Since the number of PYS per tissue in the PS3D-90 dataset is too low, we generally avoided tissue-based analyses of PYSs in the PS3D-90 dataset.

### Statistics

The R environment [30] was used for statistical analyses. For the numerical data we used the non-parametric two-sample Kolmogorov-Smirnov test, whereas the Fisher exact test was applied for the categorical data. In each tissue the occurrence of a particular property of phosphorylation sites was compared to that of non-phosphorylation sites.

### Spatial (3D) Environments of Phosphorylated and Reference (Non-Phosphorylated) Serine/Threonine/Tyrosine Residues

By calculating the occurrence of 20 different amino acid types within the radial distances of 2 to 12 Å from the phosphorylated S/T/Y residue, and excluding amino acids already present in the sequence vicinity of PSSs/PTSs/PYSs, 3D and pure 3D environments of phosphorylation sites in the PS3D-90 dataset were determined, respectively. Distances between amino acid residues were defined based on the minimal distance between any pair of atoms belonging to these residues. For both types of environment the Fisher exact test was performed to assess the significance of the differences between PSSs/PTSs/PYSs and non-PSS/non-PTSs/non-PYSs in each tissue, and we used our in-house software tool to visualize these differences efficiently. We applied the procedure described in our previous work [25] to find the propensity of each amino acid type at each radial distance ranging from 2 to 12 Å (in increments of 1Å).

### Structural Properties of Phosphorylation Sites

We extracted structural features of phosphorylation sites as described previously [25]. Briefly, we utilized NACCESS [31] to calculate the surface accessibility of phosphorylation sites and their sequence environments. DisEMBL [32] was used to predict disordered regions in each phosphorylated protein sequence. We gathered secondary structure annotations from the DSSP database [33]. Note that the number of PYSs associated with known secondary structures was not sufficient for comparison; therefore, they were excluded from this analysis.

### Analysis of Structural Folds and Functional Domains

Structural folds of phosphorylated proteins from the PS3D-90 dataset in each tissue were examined according to the *class* and *protein domain* levels of the SCOP hierarchy [34]. At the *structural class* level, the significance threshold of 0.05 was used, whereas at the *protein domain* level false discovery rate control was performed for multiple hypothesis correction in each tissue, and the significance threshold of 0.05 was used after all p-values were adjusted. Note that the numbers of PTSs and PYSs associated with known SCOP folds were not sufficient for comparison; therefore, they were excluded from this analysis.

### KEGG Pathway Analysis

Using the best-matching UniProt identifiers of each PSSs/PTSs/PYSs and non-PSSs/non-PTSs/non-PYSs in the PS1D-70 dataset, pathways obtained from Kyoto Encyclopedia of Genes and Genomes (KEGG) database [35] were analyzed across tissues, and enriched pathways were detected. False discovery rate control was performed for multiple hypothesis correction in each tissue, and the significance threshold of 0.01 was used after all p-values were adjusted.

### Kinase Analysis

In order to analyze enriched kinases across tissues, we used the substrate-matched kinase data given by Lundby *et al*. [23] where phosphorylated protein sequences were matched against motifs of known kinases using the PHOSIDA Motif Matcher toolkit [9]. This toolkit finds matches for 33 previously identified kinase motifs around each phosphorylation site in the input protein sequence. The list of matched kinases is as follows: AKT, ATM, ATR, AURORA, AURORA-A, CAMK2, CDK1, CDK2, CHK1, CHK2, CK1, CK2, ERK/MAPK, GSK3, NEK6, PKA, PKD, PLK, PLK1, and RAD53. Based on these assignments, matched kinases of 8836 out of 11097 phosphorylation sites could be determined for the PS1D-70 dataset. We compared the counts of PSSs/PTSs/PYSs in each tissue phosphorylated by each kinase to the occurrence of non-PSSs/non-PTSs/non-PYSs in the corresponding tissue. False discovery rate control was performed for multiple hypothesis correction in each kinase class, all p-values were adjusted, and the significance threshold of 0.01 after the correction was used.

Relationships between kinases, tissues and motifs were visualized by means of a tripartite graph as implemented in Cytoscape [36]. Only the motifs that are significantly enriched in each tissue (Table A in S1 Supporting Tables) and the corresponding kinase motifs provided in Phosida [9] were considered while drawing the graph.

### Tissue-Specific Expression of Kinases

For each kinase we identified the corresponding rat UniProt ID from the PhosphoSitePlus database [37], which contains experimentally identified kinase-substrate data. If no rat information was found, we attempted to find mouse and human UniProt IDs, in this order of preference. If a query kinase could not be identified in the rat, mouse or human proteomes based on the PhosphoSitePlus database, it was excluded from further analysis. Protein expression levels of kinases across tissues were obtained from the PaxDb database [38]. Since no tissue-specific expression data for rat proteins is provided in PaxDb, we used PaxDb data for the mouse orthologs of rat or human kinases obtained from the KEGG database. For some of the kinases considered in this study PhosphoSitePlus contains information for multiple isoforms (for instance, GSK3A and GSK3B isoforms for GSK3). In such cases, the expression of each isoform was analyzed separately. To assess the existence of tissue-specific kinase expression, a one-sample *t*-Test was used. The expression value of a particular kinase in a particular tissue was compared to expression values of the same kinase in all tissues. All p-values were adjusted and the significance threshold of 0.01 after the correction was used.

## Results and Discussion

### Analysis of Sequence Motifs of Global Phosphorylation Sites

**Fig 1:**
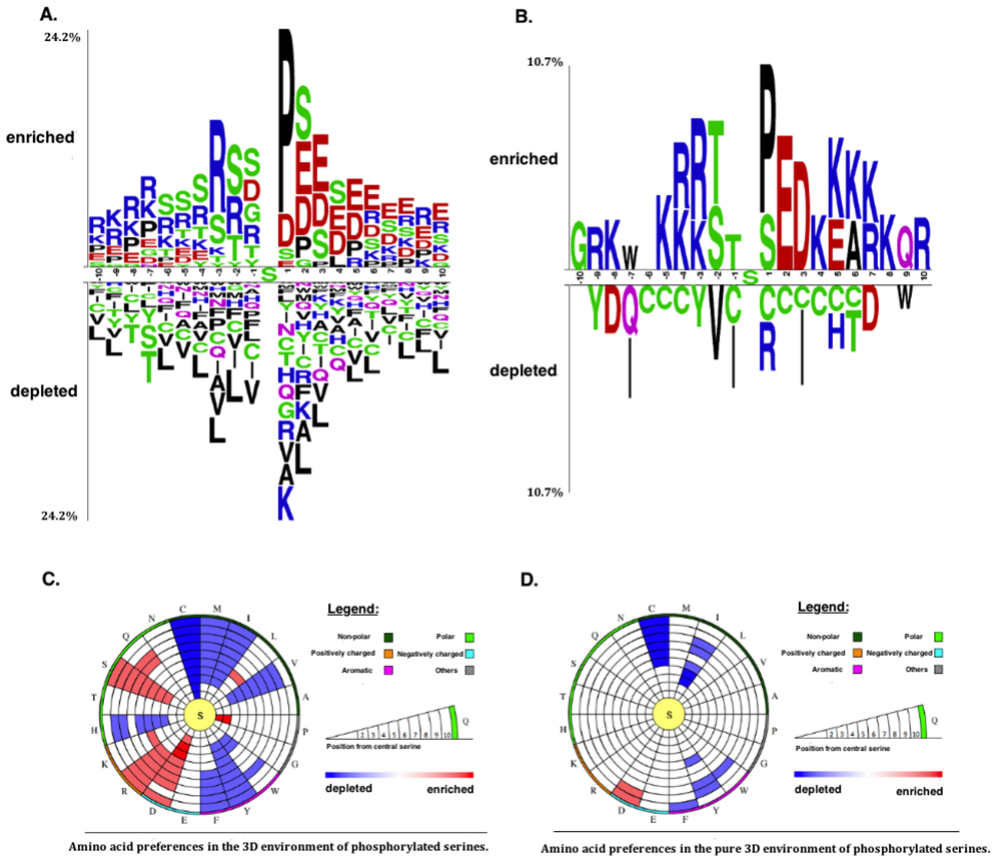
Sequence (1D) and structural (3D and pure 3D) environments of globally phosphorylated serine sites represented by two sample logos and circular plots, respectively. **(A)** Two sample logo of global PSSs in the PS1D-70 dataset. (**B**) Two sample logo of global PSSs in the PS3D-90 dataset. **(C)** 3D environments of global PSSs in the PS3D-90 dataset. **(D)** Pure 3D environments of global PSSs in the PS3D-90 dataset.

We first examined global phosphorylation trends at the sequence level based on the PS1D-70 dataset. In general our findings are in line with previous studies [11, 21, 39, 40]. Proline (P) residues are enriched at position +1 with respect to globally phosphorylated serine and threonine sites (Fig 1A and Fig A in S1 Supporting Figures). Negatively charged glutamic acid (E) and aspartic acid (D) residues as well as polar serines are also enriched in the upstream regions of phosphorylated serine, threonine and tyrosine (Y) sites (Fig 1A, and Figs A and B in S1 Supporting Figures). There is also a strong trend for charged lysine (K) and arginine (R) residues to be enriched in the sequence neighborhood of phosphorylated serines and threonines, except for the positions from +1 to +4.

### Tissue-Based Analysis of Sequence Motifs of Phosphorylated Sites

**Fig 2:**
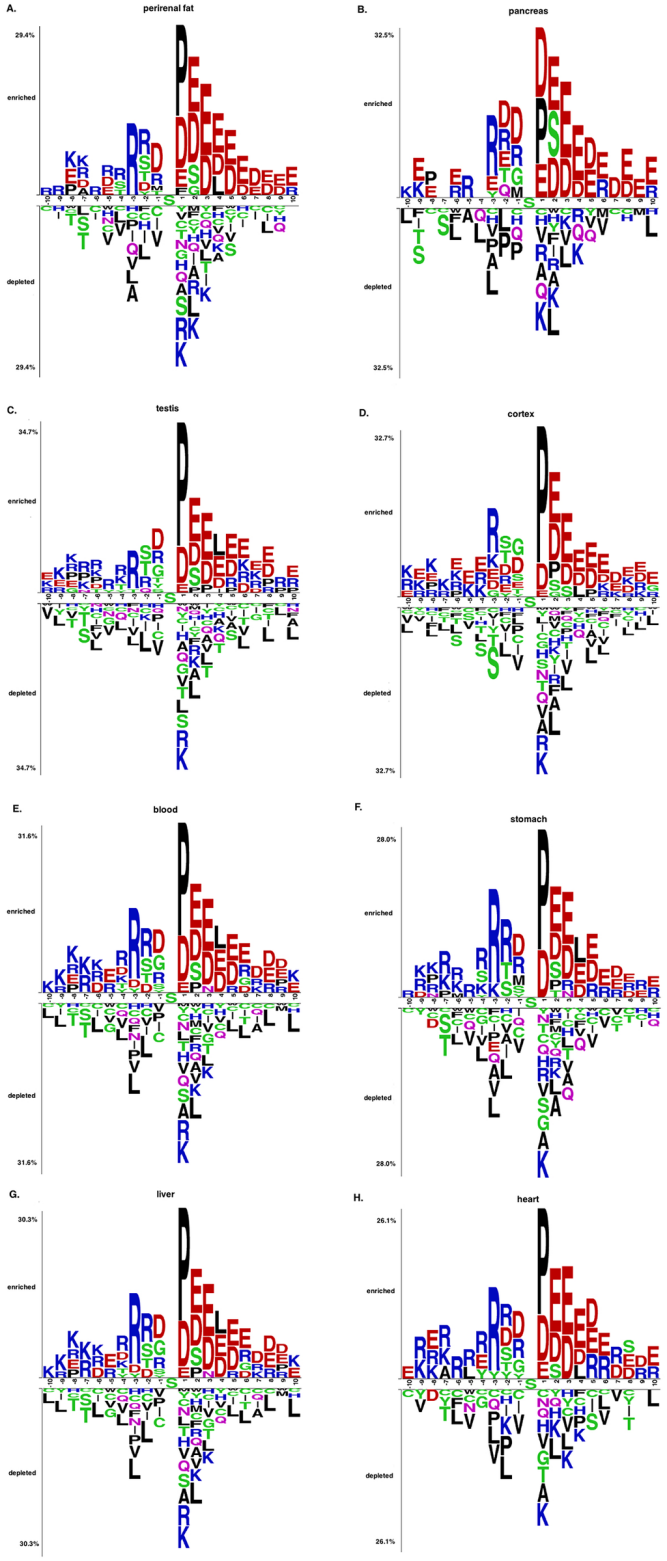
Two sample logos of PSSs from the PS1D-70 dataset in different tissues. Two sample logos of PSSs from the PS1D-70 dataset in perirenal fat **(A)**, pancreas **(B)**, testis **(C)**, cortex **(D),** blood **(E)**, stomach **(F)**, liver **(G)** and heart **(H)**. See data on other tissues in Figs C–T in S1 Supporting Figures.

Previous studies have shown that beyond the general trends, phosphorylation sites exhibit kinase-specific sequence and spatial motifs as well as compartment-specific sequence motifs [6, 7, 21]. The availability of experimentally identified tissue-specific phosphorylation sites has now enabled us to examine phosphorylation trends across tissues. Our analysis of the PS1D-70 dataset revealed clear tissue-specific preferences. For instance, only PSSs in perirenal fat have phenylalanine (F) residues at position +1 (Fig 2A), whereas only PSSs in pancreas and testis have a preference for glutamine (Q) residues at position -2 (Fig 2B and 2C). Only in cortex, glycine (G) residues are enriched at position -3 with respect to central phosphorylated serine sites (Fig 2D). Histidine residues are only enriched at position +6 in blood (Fig 2E), whereas PSSs in stomach and liver show preferences for asparagine (N) residues at position +3 (Fig 2F and 2G). In contrast to the global trends, none of the tissue-specific phosphorylation sites show any enrichment for serine residues at position +1 (See Figs C–T in S1 Supporting Figures for more detailed graphs for these and other tissues). Tissue-specific trends for PTSs are also quite prominent. For instance, only PTS in cortex show a preference for methionine (M) residues at position -6, whereas asparagine residues are only strongly preferred at positions -7 and -8 only in muscle (Fig U in S1 Supporting Figures). Glutamine residues are enriched for PTSs in blood at position -7.

We utilized the Motif-X software to identify enriched sequence motifs that are exclusively tissue-specific and cannot be detected when analyzing global trends (Table A in S1 Supporting Tables). For instance, the motifs pS-P-E, pS-P-X-X-E, E-X-X-X-X-X-X-X-X-pS-P, E-X-X-X-X-pS, pS-X-X-X-X-X-X-X-X-D, pS-X-X-X-X-X-X-X-X-E, E-X-X-X-X-X-X-X-X-pS and pS-X-X-X-X-X-X-X-X-K are only associated with brain-specific PSS. On the other hand, the motif pS-X-X-X-X-D is only observed in PSS in liver. These observations indicate that individual tissues harbor specific sequence environments for phosphorylation.

Lundby *et al*. have already investigated sequence motifs of phosphorylation sites in two tissues—brain and testis—but in their work comparison was performed between tissue-specific and global phosphorylation sites. Using global sequence signatures of phosphorylation sites as a background dataset for studying tissue-specific motifs may fail to reveal potential systematic biases existing in the foreground dataset and can lead to random and non-informative motif signals [41]. In this work we investigate the enrichment or depletion of phosphorylation sites relative to their non-phosphorylated counterparts in each individual tissue, thus ensuring that the trends found are not due to tissue-dependent variation of protein abundance or individual biological roles of proteins. In other words, enriched motifs identified through this approach are solely due to phosphorylation processes occurring in a particular tissue and not any other tissue-specific properties. We detected 15.4% and 17.1% more enriched/depleted residues in the sequence environment of phosphorylation sites in brain and testis, respectively, based on two sample logos, even though some trends for certain residues at particular positions overlap in both studies (58.8% and 57.5% in brain and testis, respectively) (see Tables B and C in S1 Supporting Tables). Note that we applied separate two sample logo analyses for serine and threonine sites, whereas Lundby *et al*. combined both residues. Furthermore, discriminative motif analysis using the Motif-X software yielded qualitatively different tissue-specific motifs compared to the motifs obtained in the previous study by Lundby *et al*. One of the most significant motifs we identified—E-X-X-X-X-pS—is only observed in brain but not in any other tissue (Table A in S1 Supporting Tables), whereas residue E in the corresponding position has not been even found enriched using the former method (X and pS here represent any residue and phosphorylated serine, respectively). We found that the residue G is enriched at position -1 in testis and the positively charged residues R and K are enriched at almost all positions in downstream regions of phosphorylated serines in both brain and testis, whereas none of these effects could be observed using global phosphorylation sites as a background set. Similarly, residue P is enriched in all tissues at position +1 in our analysis; however, Lundby *et al*. found P to be enriched at that position in brain, but not in testis. We also found the motif pS-X-X-R to be highly specific for testis, while the residue R is in fact depleted in the corresponding position in the previous study.

High specificity of kinase action is an important prerequisite for exquisite regulation of signal transduction processes [42]. In accordance with this notion, compartment-specific kinases have been proposed in a previous work [21], whereas the existence of compartment- and tissue-specific acetyltransferases (KATs) has also been discussed [22, 25, 43]. The substrate sequence specificity among tissues identified in this work suggests the existence of tissue-specific kinases and phosphatases.

### Tissue-Based Analysis of Phosphorylation Sequence Motifs in Proteins with Known 3D Structure

**Fig 3:**
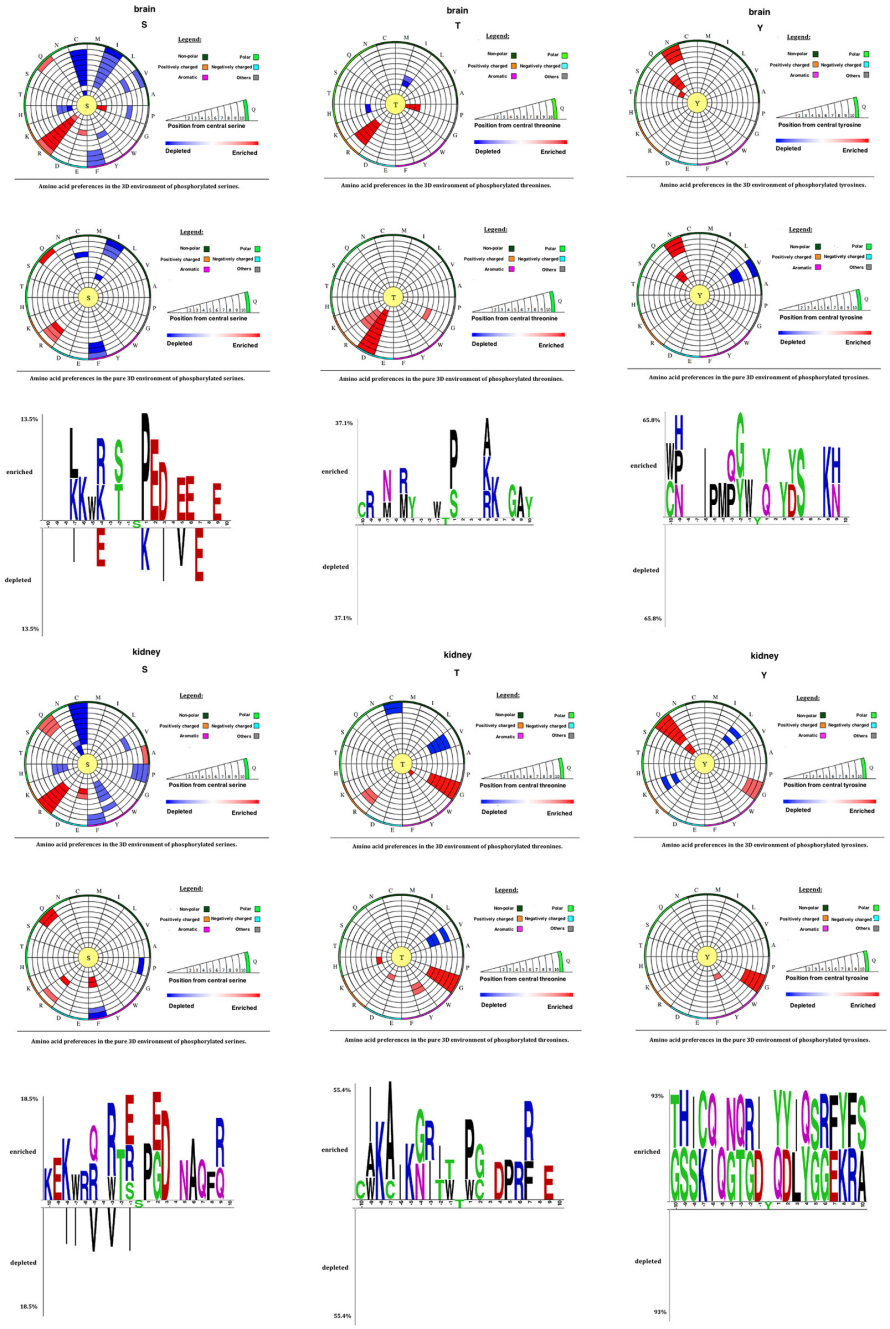
Sequence (1D) and structural (3D and pure 3D) environments of PSSs, PTSs, and PYSs from the PS3D-90 dataset represented by two sample logos and circular plots, respectively. Data for brain and kidney is shown (see S1 Supporting Figures for all tissues). The circular amino acid propensity plots were produced by our in-house software tool. Color code for amino acid residue enrichment in the circular plots: (i) red–enriched, if p-value < 0.05 and odds ratio > 1, (ii) blue–depleted, if p-value < 0.05 and odds ratio < 1, and (iii) white–neither enriched nor depleted, if p-value ≥ 0.05.

As a pre-requisite for the investigation of structural trends (see below), we performed a separate analysis of phosphorylation sequence motifs in the subset of proteins possessing a known 3D structure (dataset PS3D-90). The results obtained are markedly different from the ones derived from the PS1D-70 dataset due to the fact that structurally characterized proteins are depleted in disordered regions, which leads to a different amino acid composition. Specifically, negatively charged residues, as well as disorder-related glycine and serine residues are less pronounced in this dataset. For instance, aspartic acid and glutamic acid are not observed at position +1 of the global and tissue-specific PSSs (Figs 1B and 3, and Fig W in S1 Supporting Figures). Lysine and alanine (A) residues are enriched at positions +4 and +6 of the global PSSs in the PS3D-90 dataset, whereas they are not enriched at the same positions of the global PSSs in the PS1D-70 dataset, where lysine residues are even depleted at position +4. Glutamic acid residues are depleted at positions -4 and +7 for structurally known PSSs in brain, whereas they are enriched at these positions in the PS1D-70 dataset. PSS in heart have strong preferences for alanine and proline residues at positions -4 and -3, respectively, whereas such preferences could not be observed in the PS1D-70 dataset, and proline residues are even depleted at position -3. Asparagine and glutamine residues are generally enriched in the upstream regions of structurally known tissue-specific sites, whereas they are generally depleted in the PS1D-70 dataset.

### Tissue-Specific Spatial Motifs of Phosphorylation Sites

Previous studies found only minor differences between the 3D structural surroundings of phosphorylated and non-phosphorylated sites, whereas stronger trends were observed when taking into account kinase preferences [6]. In this study we detected more significant spatial motifs associated both with global and tissue-specific PSSs. In accordance with [6], arginine, proline, leucine and serine residues are enriched in the spatial environment of global PSSs, but we also found aspartic acid, lysine and glutamine residues to be enriched (see Fig 1C). Frequent occurrence of aspartic acid residues has also been observed in 1D and 3D environments of PSSs in previous studies [7, 23].

Similar to the trend in the sequence motifs discussed above cysteine (C) residues are strongly avoided in the spatial surroundings of PSSs in all tissues. Strong enrichment of proline and aspartic acid residues is observed in close proximity of global PSSs (around 2–5 Å away, see Fig 1C), which again parallels the trends in sequence motifs. However, the enrichment of leucine residues at a distance range of 6 to 7 Å is not found in the sequence environment. On the other hand, no enrichment of glutamic acid is observed in the 3D environment even though glutamic acid is highly enriched in the sequence motifs of global PSSs.

We also found tissue-specific trends in the spatial environments of phosphorylation sites (Fig 3, and Figs Z, AA and AB in S1 Supporting Figures). PSSs in brain have a strong preference for arginine residues, and it is much more predominant than the enrichment in the 1D environment. Histidine and cysteine residues are strongly depleted in the 3D environment of PSSs in kidney, whereas no preference for these residues is observed in the 1D environment. Thus, patterns of amino acid usage around phosphorylated sites are generally consistent between 1D and 3D environments, although some of the tendencies found at the spatial environment are not observed in the 1D environment alone.

In order to disentangle the influence of local amino acid content from the effects caused by spatial proximity, we performed a separate analysis of *pure* structural environments (see Methods). While only a weak enrichment of certain amino acid residues is observed in global pure 3D environments of PSSs (aspartic acid, Fig 1D) and PTSs (aspartic acid and glycine, Fig A in S1 Supporting Figures), tissue-specific preferences are more clear cut (Figs AC, AD and AE in S1 Supporting Figures). For instance, aspartic acid residues at a distance between 3 Å and 12 Å with respect to PTSs are strongly enriched in brain, which is not observed when sequence context is also considered. Similarly, PTSs residing in kidney have a preference for histidine only when their pure 3D environments are considered. These findings imply that there are statistically significant patterns in spatial residue preferences of phosphorylation sites in addition to significant sequence patterns. Previous studies have shown that the spatial context plays a role in the recognition of substrates by kinases [6, 7]. Here we found that the amino acid composition in 3D varies in a tissue-dependent manner, which implies that both sequence and spatial environments of phosphorylation sites may play a role in determining the substrate-specificity of kinases and phosphatases across tissues.

### Structural Properties of Phosphorylation Sites

It has been previously shown that phosphorylation sites generally reside in unstructured and disordered regions [44, 45]. We assessed the disordered region preferences of phosphorylation sites in the PS1D-70 dataset, where disordered regions were predicted from protein sequences. We found that PSSs and the residues surrounding them follow the same tendency in all individual tissues (Fig AF in S1 Supporting Figures), while PTSs display the preference for disordered regions in all tissues except for muscle (Fig AG in S1 Supporting Figures).

Based on the experimental structures of phosphorylated proteins, we also analyzed structural properties of phosphorylation sites in the PS3D-90 dataset. However, we were only able to assess the global preferences of phosphorylated sites since the datasets of proteins with known 3D structure phosphorylated in a tissue specific manner were not large enough to obtain statistically significant results.

Global PSSs, PTSs and PYSs along with the residues surrounding them are consistently and significantly more solvent exposed than non-PSSs, non-PTSs and non-PYSs and their 1D environments by about 30.73% (p < 2.2 x 10^−16^), 27.05% (p < 6.9 x 10^−6^) and 30.02% (p < 1.5 x 10^−3^) on average, respectively (see Tables D, E and F in S1 Supporting Tables), which is in line with previous reports [6, 46, 47]. We also found that PSSs preferentially occur in more flexible regions of protein structures, as assessed by the B-factor analysis. Globally phosphorylated serine sites and the residues surrounding them in a very close proximity have larger B-factor values than their non-phosphorylated counterparts do (Table D in S1 Supporting Tables). Globally phosphorylated PSSs and PTSs in the PS3D-90 dataset display a clear tendency to reside in loops (Figs AI and AJ in S1 Supporting Figures).

### Phosphorylated Proteins Take Part in Tissue-Specific Biological Pathways

**Fig 4:**
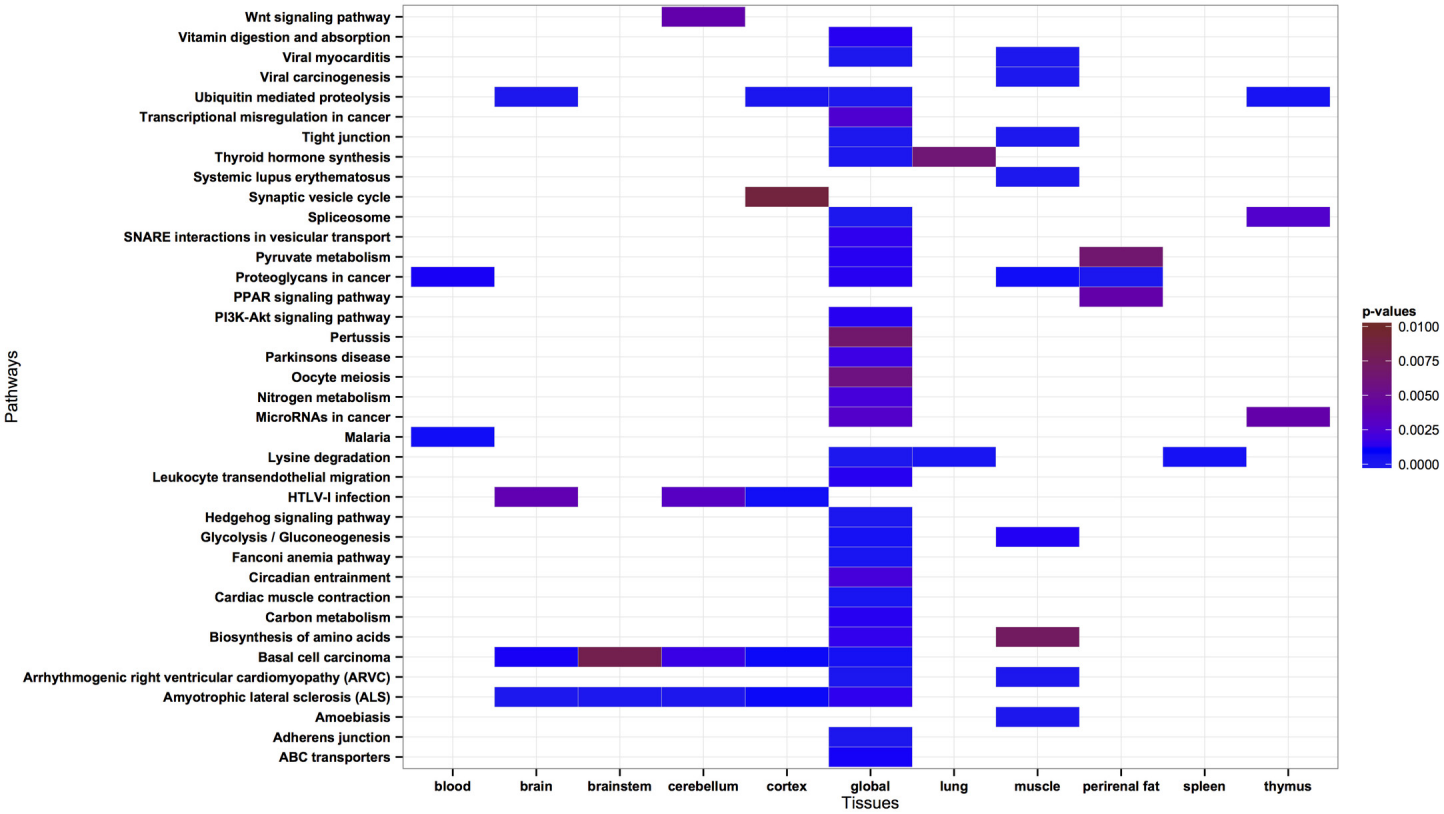
KEGG pathway analysis of the serine phosphorylated proteins from the PS1D-70 dataset. Pathways with a corrected p-value < 0.01 in each tissue are considered significantly enriched. Insignificant results are represented by white color. Only the tissues with at least one significantly enriched pathway are shown.

Previous studies have shown that the structural environment of phosphorylation and acetylation sites plays an important role in determining kinase/acetyltransferase specificity and ultimately the functional role of phosphorylation and acetylation processes [4, 7, 25]. Hence, we conducted a tissue-based analysis of phosphorylated proteins to assess their specialization for various functions in different tissues. Following our previous work [25], we compare phosphorylated proteins in specific pathways to non-phosphorylated counterparts in each individual tissue (see Methods), which makes this analysis independent from the mere presence or absence of certain functions in the respective tissues. Crucial roles of phosphorylated proteins in membrane transport, metabolism, signaling pathways, and their disease related pathways are in general well-known. We found that globally phosphorylated serine sites are linked to various processes, including ABC transporters activities, adherens junction formation, cardiac muscle contraction system, energy generation processes of glycolysis/gluconeogenesis, pyruvate metabolism and lysine degradation, hedhedog signaling pathway, leukocyte transendothelial migration system, tight junction, and ubiquitin mediated proteolysis (see full list in Fig 4). Proteins carrying serine phosphorylation are associated with disease processes and macromolecules affecting tumor progress, including the regulation of arryhythmogenic right ventricular cardiomyopathy (ARVC) and basel cell carcinoma diseases, and proteoglycans in cancer.

Serine phosphorylated proteins display distinctly different biological pathway preferences both in a global and tissue-specific manner. For example, proteins involved in synaptic vesicle cycle show significant enrichment of PSSs only in cortex. Only proteins phosphorylated on serine residues in muscle play a role in regulation of diseases including amoebiasis, systematic lupus erythematosus and viral carcinogenesis, whereas only phosphorylation in blood appears to be involved in the regulation of malaria disease, which is a parasitic protozoans-caused disease transmitted by the biting of mosquitoes [48].

Proteins globally phosphorylated on threonine residues are involved in energy metabolism and disease related pathways, including viral myocarditis, viral carcinogenesis and tight function processes. Tissue-specific preferences can only be detected for phosphoproteins in muscle where they take part in the regulation of diseases such as amoebiasis, arrhythmogenic right ventricular cardiomyopathy (ARVC) and systematic lupus erythematosus (Fig AK in S1 Supporting Figures). These findings show that biological processes are regulated by phosphorylation in a tissue dependent manner. Kinases are one of the most important drug targets for a number of diseases, including cancer, hypertension, Parkinson’s disease, and autoimmune diseases [1]. The fact that phosphoproteins exhibit tissue-specific preferences in the regulation of disease pathways implies that designing drugs targeting tissue-specific disease pathways may be a promising avenue towards improved and more specific therapeutic effects, as suggested previously [25].

In order to assess the association between biological pathways and functional domains harboring phosphorylated sites, we first analyzed statistical preferences at the top level of the SCOP hierarchy, structural class. Except for the slight enrichment of cerebellum-, brain- and blood-specific PSSs in all-α proteins, we could not detect any significant trends (Fig AM in S1 Supporting Figures). At the second level of the SCOP hierarchy, which reflects structural folds, we identified a number of significant preferences for PSSs, which parallel tissue-specific biological pathway preferences described above. Protein domains found to be phosphorylated in a tissue-specific manner include fructose-1,6-bisphosphate aldolase domain, which belongs to the glycolysis pathway, and creatine kinase C-terminal domain, which is a key enzyme responsible for intracellular energetic homeostasis of vertebrate excitable tissues and for catalysis of the reversible transfer of the high-energy phosphate between the ATP/ADP and creatine/phosphocreatine systems [49] (Fig AN in S1 Supporting Figures). The beta-chain of hemoglobin is phosphorylated on serine residues only in blood, which may imply the relevance of phosphorylation for the oxygen transport function. The cytosolic class alpha glutathione S-transferase (GST) domain resides in proteins phosphorylated on serine residues only in liver where the GST domain catalyzes the joining of glutathione to xenobiotic substrates for detoxification. GSTs also play a role in cell signaling and the overexpression of GSTP1-1 –an isozyme of the mammalian GST family—has been associated to cancer [50], making it a potential drug target and warranting an experimental investigation of its phosphorylation. Globally phosphorylated proteins carrying threonine phosphorylation harbor the same domains as serine phosphorylated proteins do, but their tissue-specific preferences differ (Fig AO in S1 Supporting Figures). For instance, only phosphoproteins in thymus include the histone H2A domain, which gains function in DNA repair after serine phosphorylation of one of its variants [51]. We therefore propose that threonine phosphorylation in thymus might also generate new functions of the H2A proteins. The adipocyte lipid-binding protein (ALBP), which is a carrier protein in fatty acid metabolism and has roles in many diseases [52], creatine kinase N-domain, which is an important enzyme in energy-consuming processes, and cAMP-dependent PK catalytic subunit domain, which is essential for phosphorylation of some proteins [53], are phosphorylated on threonine residues only in perirenal fat.

### Kinases Target Tissue-Specific Phosphorylation Sites

**Fig 5:**
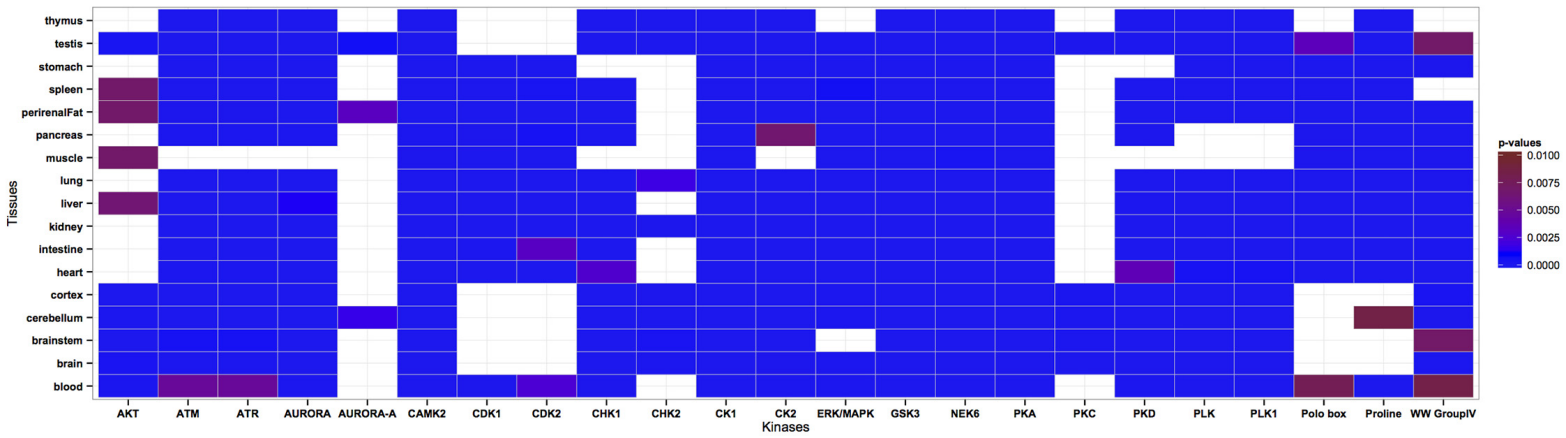
Tissue preferences of kinases targeting serine phosphorylated sites in the PS1D-70 dataset. Tissues with a corrected p-value < 0.01 in each kinase class are considered significantly enriched for the corresponding kinase classes. Insignificant results are represented by white color. See S2 Data for corrected p-values.

**Fig 6:**
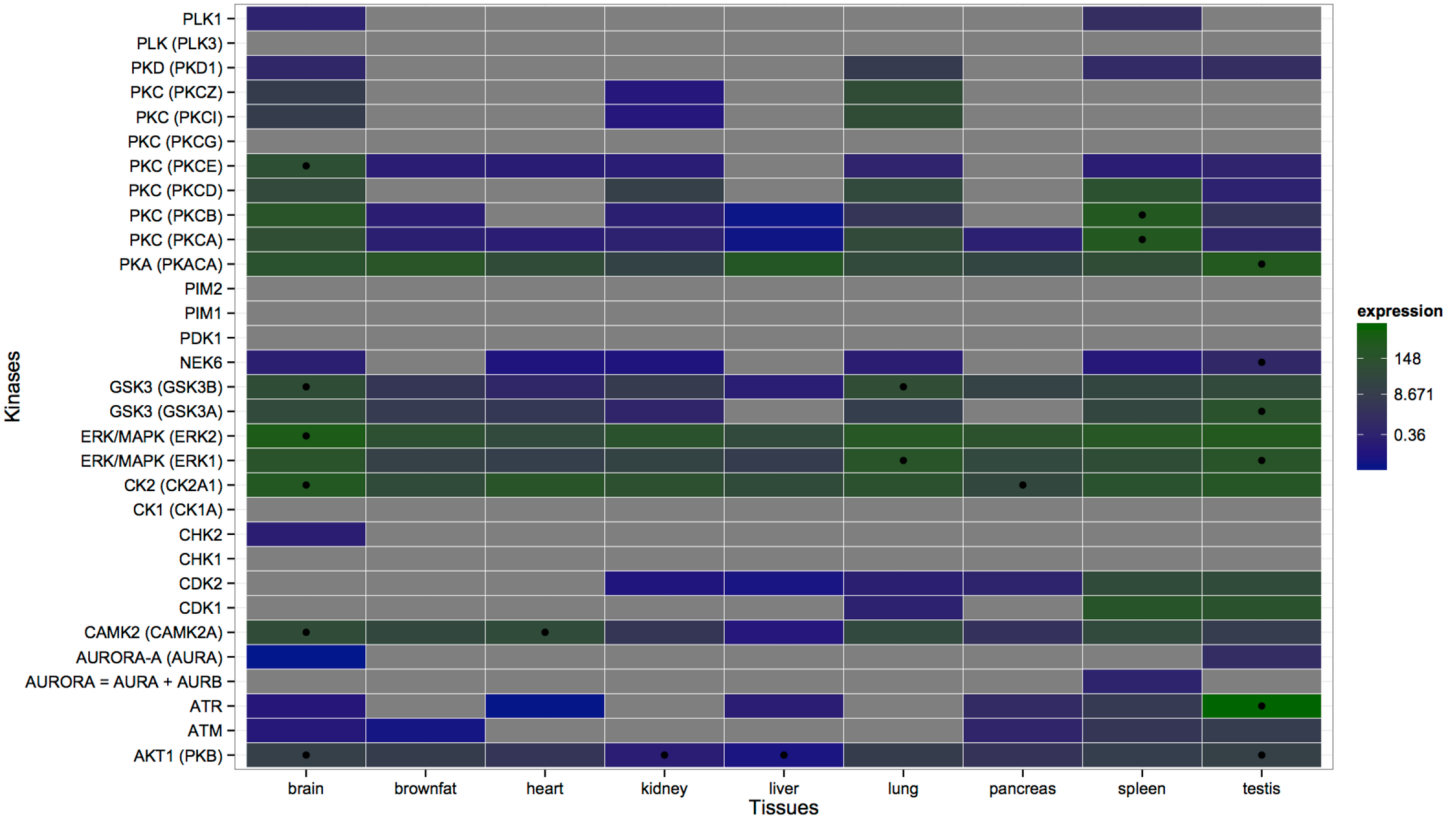
Comparison of the Ser/Thr kinase expression across tissues. Expression values are colored in log scale. Kinases with a corrected p-value < 0.01 in each tissue are considered significant and are indicated with black circles. Grey rectangles represent kinases whose expression values could not be found in the downloadable dataset of the PaxDb database. Note that only the expression values of tissues drawn here could be found in the PaxDb database. The median value 8.671 is assumed as the midpoint, calculated from all expression values in this heatmap. See S2 Data for expression values and corrected p-values.

Echoing our previous study, in which we proposed the existence of tissue-specific lysine acetyltranferases (KATs) and lysine deacetylases (KDACs) [25], the findings presented here imply the existence of tissue-specific protein kinases and phosphatases. The wealth of information on kinase-substrate association enabled us to examine kinase classes in different enriched tissues. Lundby *et al*. matched known sequence motifs of kinases to identified sequence motifs of tissue-specific phosphorylation and observed differential involvement of kinases across tissues. Based on the association between phosphorylation sites and kinases provided by Lundby *et al*., we examined tissue-specific target site preferences of kinases. Many kinases, including CK1, GSK3, NEK6, and PKA, mediate serine phosphorylation in all tissues, while some other kinases show tissue-specific preferences (Fig 5). For instance, phosphorylation sites targeted by AURORA-A are only prominent in cerebellum, perirenal fat and testis, whereas PKC mediated phosphorylation is particularly pronounced in brain (as well as in brainstem, cerebellum and cortex) and testis. Similar to the study by Huttlin *et al*. [45] where tissue-specific protein expression and phosphorylation were shown to be uncorrelated, we compared the expression of each kinase in all tissues and found that the observed tissue-specific kinase preferences in phosphorylation are not caused by tissue-specific kinase expression (Fig 6). For instance, even though PKC mediated phosphorylation is only prominent in testis and brain (also including brainstem, cerebellum and cortex), the expression levels of PKCA (an isoform of PKC) are quite similar in all tissues except for spleen where it is more strongly expressed. Conversely, we found phosphorylation sites targeted by AKT1 in many tissues including spleen and liver (Fig 5), whereas AKT1 itself is less expressed in liver relative to other tissues and its expression level is similar in spleen and other tissues (Fig 6). Note that expression levels of kinases have been obtained from different organisms due to the lack of tissue-specific protein expression data for rat; our results are thus likely to be biased due to the differences in expression levels between different organisms. Moreover, our analysis clearly does not catch higher order effects, such as cooperativity and multiplicity of kinase action.

**Fig 7:**
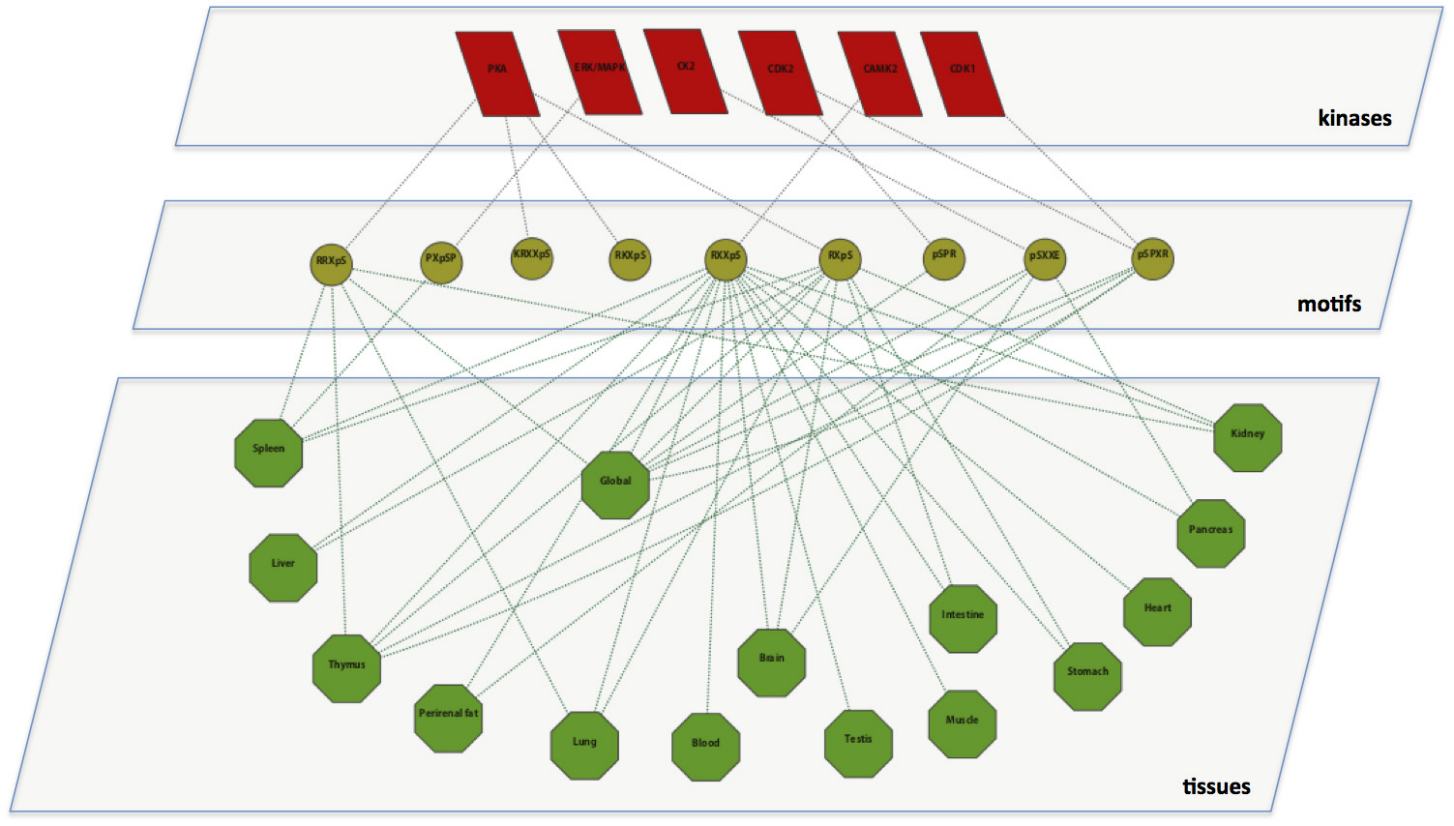
Tripartite graph showing interactions between serine phosphorylation motifs, kinases and tissues. The red parallelograms, yellow circles and green octagons represent kinases, motifs and tissues, respectively. Phosphorylated serine residues in motifs are represented with *pS*. Threonine and tyrosine phosphorylation sites are not shown in the network since no match between consensus kinase motifs and enriched tissue motifs was found.

To better understand the kinase-tissue association, we built a tripartite graph joining kinases, motifs and tissues as nodes (Fig 7). Edges between kinases and motifs indicate that a consensus motif for the corresponding kinase is provided in the PHOSIDA database [9]. Edges between motifs and tissues mean that a given tissue contains phosphorylation sites enriched for the corresponding motifs in their sequence environment (as given in Table A in S1 Supporting Tables). Note that this graph shows only a very small currently available subset of the general network connecting motifs, kinases and tissues. Nevertheless, we discovered some remarkable trends, such as the enrichment of the motif RXXpS in all tissues. This motif serves as a hub motif on the network, but only the kinase CAMK2 targets the phosphorylation sites containing the motif RXXpS in their environments. Phosphorylation sites in liver are enriched for the motif RXpS in addition to the motif RXXpS, and as a result it can be inferred that the kinases PKA and CAMK2 are highly active in liver. The motif PXpSP, is only targeted by the kinase ERK/MAPK in spleen. In pancreas the motifs pSXXE and RXXpS are enriched, which are required by the kinases CK2 and CAMK2. The kinases CDK1 and CDK2 target the motif pSPXR both globally and in a thymus-specific fashion. Note that our analysis of kinase-motif associations is solely based on the results of the *De Novo* Motif Finder tool of the PHOSIDA database.

## Conclusions

Here we present a comprehensive study of phosphorylation sites at the sequence and structure level in 14 rat tissues based on the proteomics data recently published by Lundby *et al*. [23]. We show that phosphorylation sites display tissue-specific preferences for certain residues in their linear amino acid sequence. Primary sequence motifs of phosphorylation sites in two tissues—brain and testis—have already been investigated by Lundby *et al*., but in their work the comparison was performed between tissue-specific and global phosphorylation sites. By contrast, in this work we compare the enrichment or depletion of phosphorylation sites to their non-phosphorylated counterparts in each individual tissue, which allowed us to uncover some previously unnoticed trends. Beyond the known tendency of phosphorylation sites and the residues surrounding them to reside in disordered regions and irregular secondary structures, we also identified tissue-specific preferences for certain residues in their spatial environments. In addition to the previously described tissue-specific sequence motifs targeted by kinases [23, 45], our findings would seem to indicate that tissue-specific spatial motifs in the substrates also play a role in kinase targeting. We also demonstrate that while many kinases mediate phosphorylation in all tissues, there are also kinases that operate in a tissue-specific manner. Interestingly, tissue-specific kinase preferences are not correlated with tissue-specific kinase expression. The tripartite graph connecting kinases, tissues and motifs reveals that some motifs are prominent in many tissues, but are only targeted by few kinases.

Similar to our previous work, in which we postulated that tissue-specific KATs and KDACs may control lysine acetylation [25], the findings presented here both at the sequence and structure level confirm the existence of tissue-specific protein kinases and phosphatases, as initially suggested by Huttlin *et al*. [45]. Given the strong dependence of protein function on tissue context, it appears plausible that the intricate processes involved in kinase action, including the regulation of the catalytic domain by the hydrophobic spines [54], co-localization of the kinase and the substrate as a pre-requisite for substrate recruitment [42], substrate sequestration or masking, which modulate availability for phosphorylation, may be tissue-specific. However, our analysis of the associations between kinases and amino acid sequence motifs failed to reveal any clear preferences of kinases for individual tissues. We therefore speculate that tissue specialization for kinase-substrate binding may be encoded at the 3D structure level.

## Supporting Information

S1 Data Protein Uniprot Ids used in PS1D-70 and PS3D-90 datasets. (XLSX)

S2 Data Expression values and tissue preferences of kinases with corrected p-values in the PS1D-70 dataset. (XLSX)

S1 Supporting Figures All supplementary figures. (DOCX)

S1 Supporting Tables All supplementary tables. (DOCX)
