# Supplementary material for: Sequence- and Structure-Based Analysis of Tissue-Specific Phosphorylation Sites
Source: PLoS One. 2016 Jun 22;11(6):e0157896. doi: 10.1371/journal.pone.0157896 (PMC4917084; doi:10.1371/journal.pone.0157896)
Supplement: S1 Supporting Figures — (DOCX) [file pone.0157896.s003.docx]

**SUPPLEMENTARY FIGURES**

**
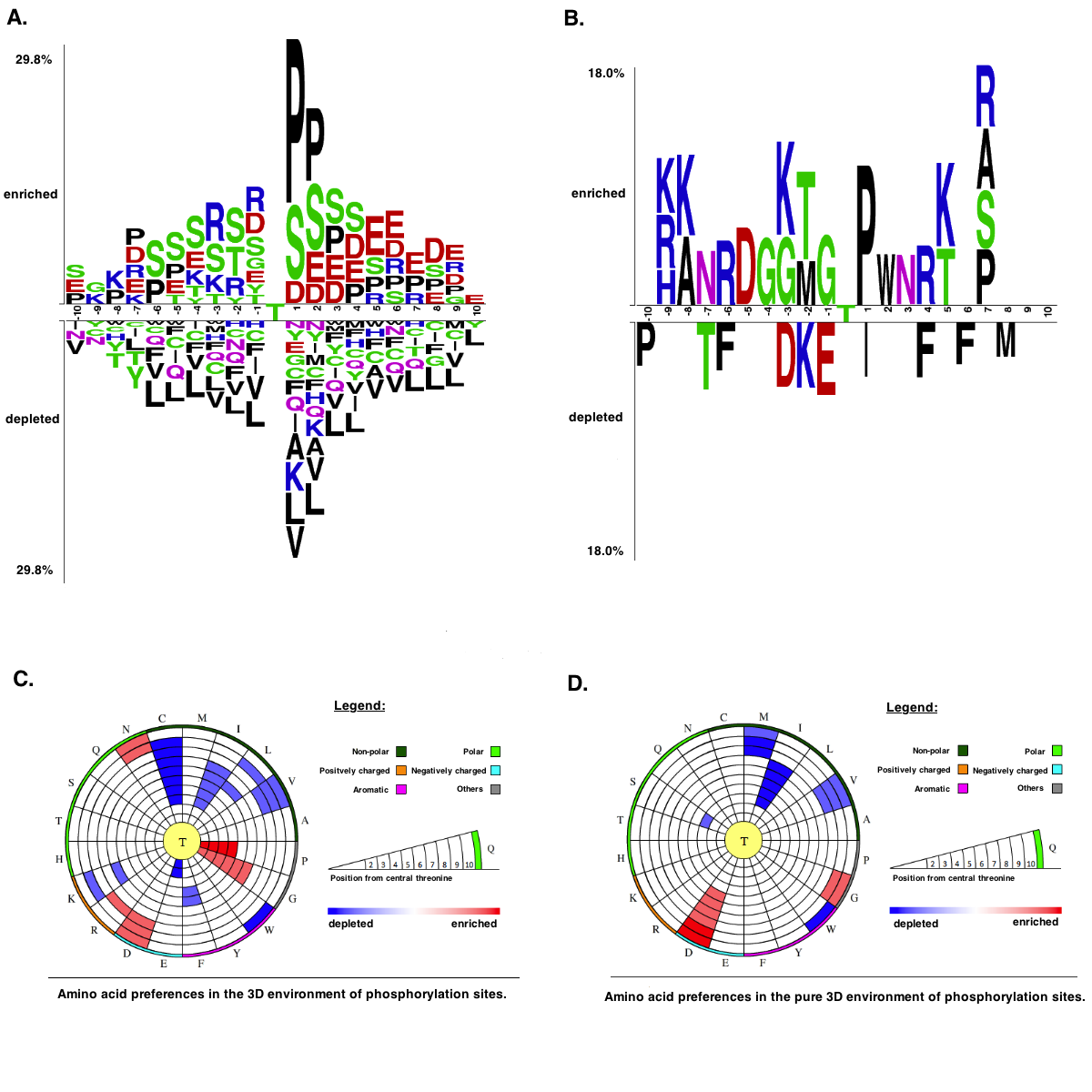
**

**Fig. A.** Two sample logo analysis of global PTSs in the PS1D-70 dataset (**A**) and in the PS3D-90 dataset (**B**), 3D **(C)** and pure 3D **(D)** environments of PTSs in the PS3D-90 dataset.

**
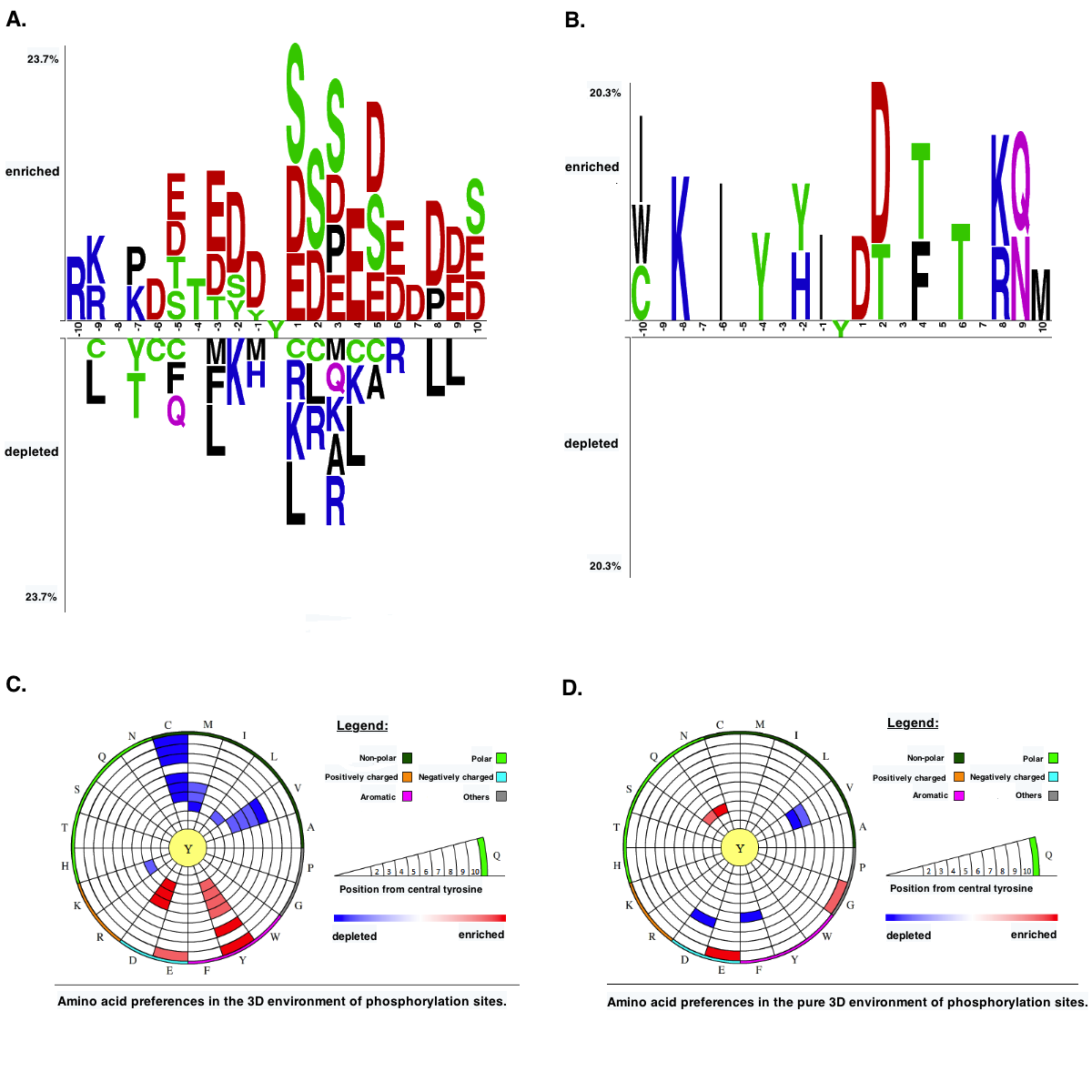
**

**Fig. B.** Two sample logo analysis of global PYSs in the PS1D-70 dataset (**A**) and in the PS3D-90 dataset (**B**), 3D **(C)** and pure 3D **(D)** environments of PYSs in the PS3D-90 dataset.

**
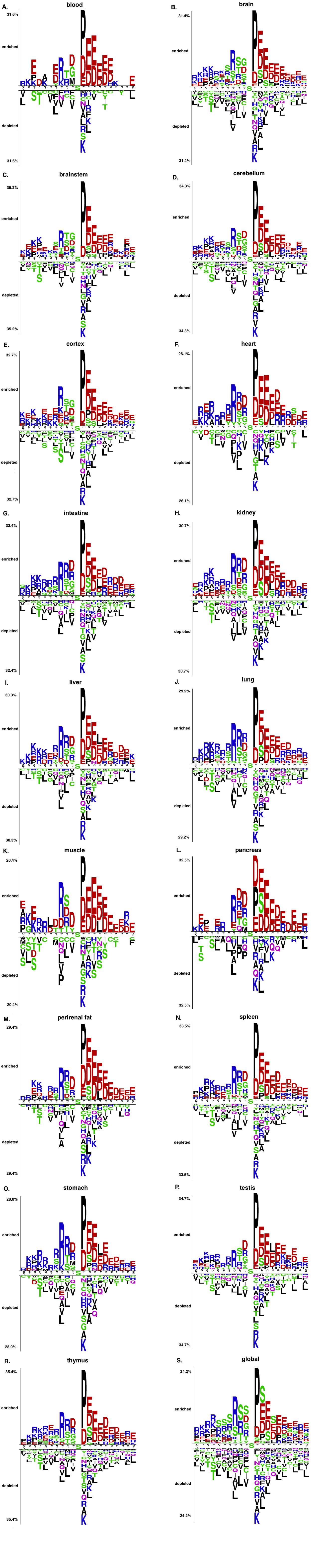
**

**Fig. C.** Two sample logo analysis of PSSs in different tissues in the PS1D-70 dataset (See Fig. D – Fig. T for high resolution graphs).


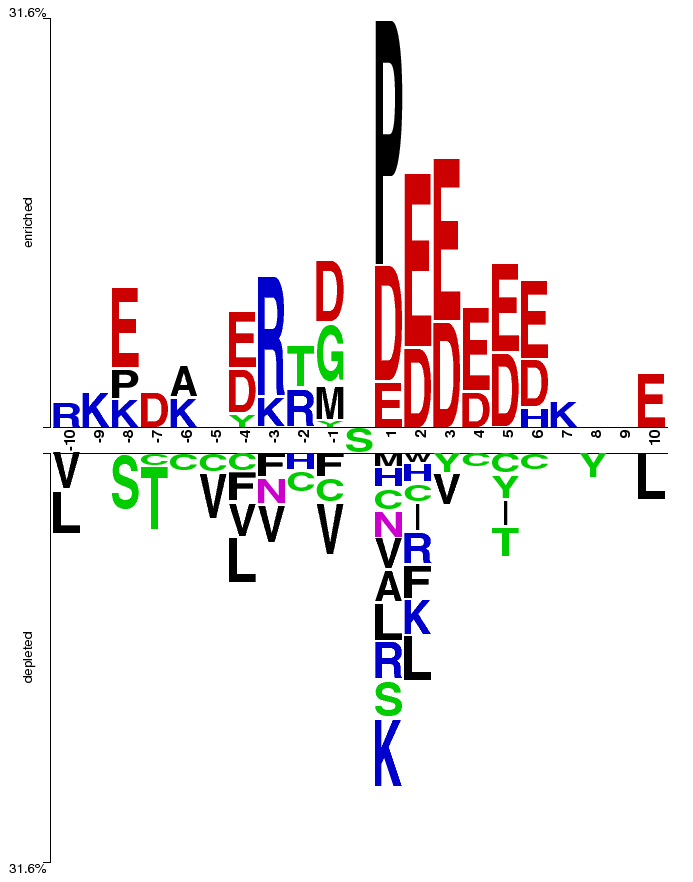


**Fig. D.** Two sample logo analysis of PSSs from the PS1D-70 dataset in blood.


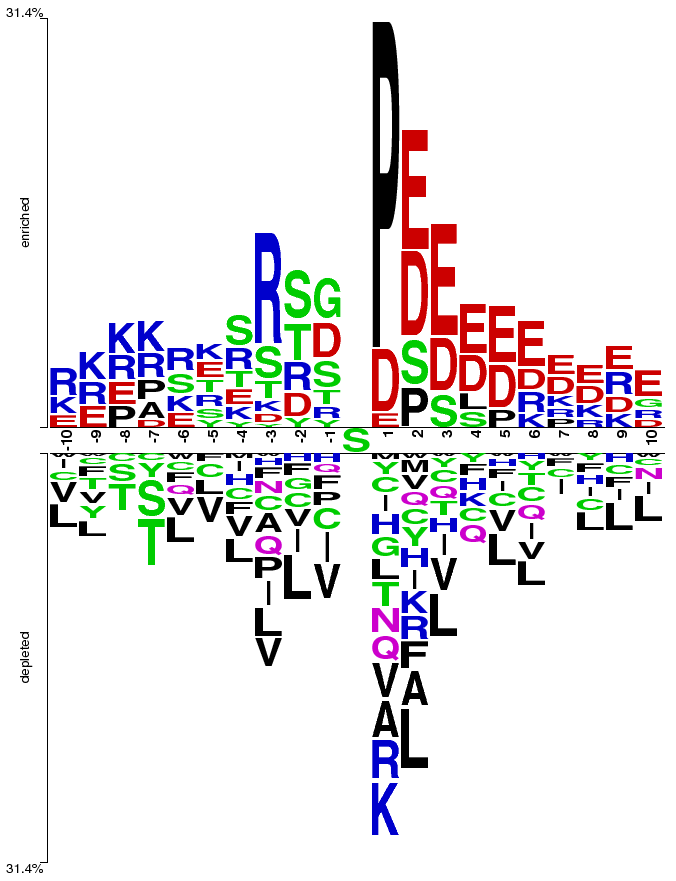


**Fig. E.** Two sample logo analysis of PSSs from the PS1D-70 dataset in brain.


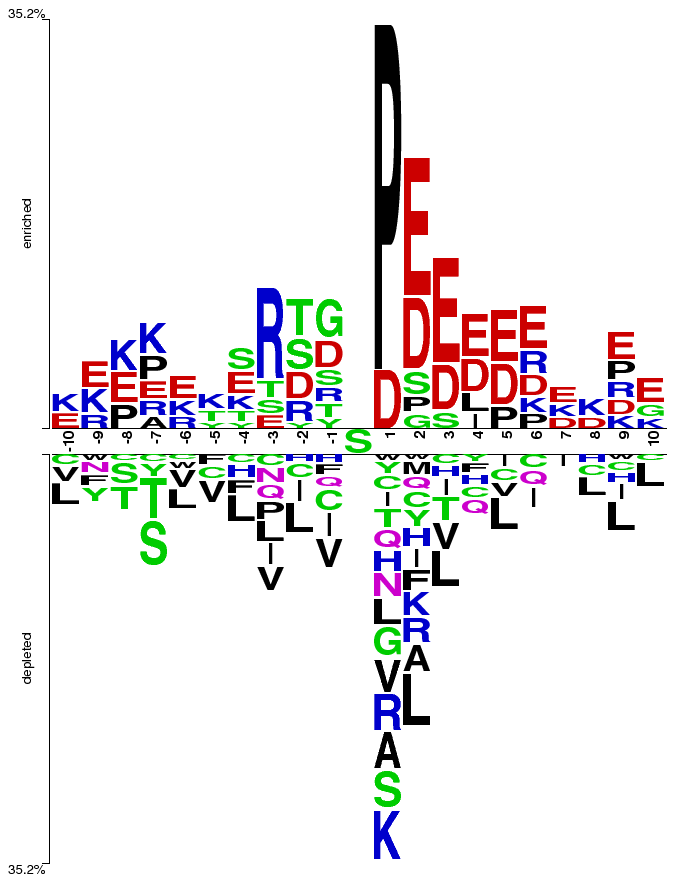


**Fig. F.** Two sample logo analysis of PSSs from the PS1D-70 dataset in brainstem.

**
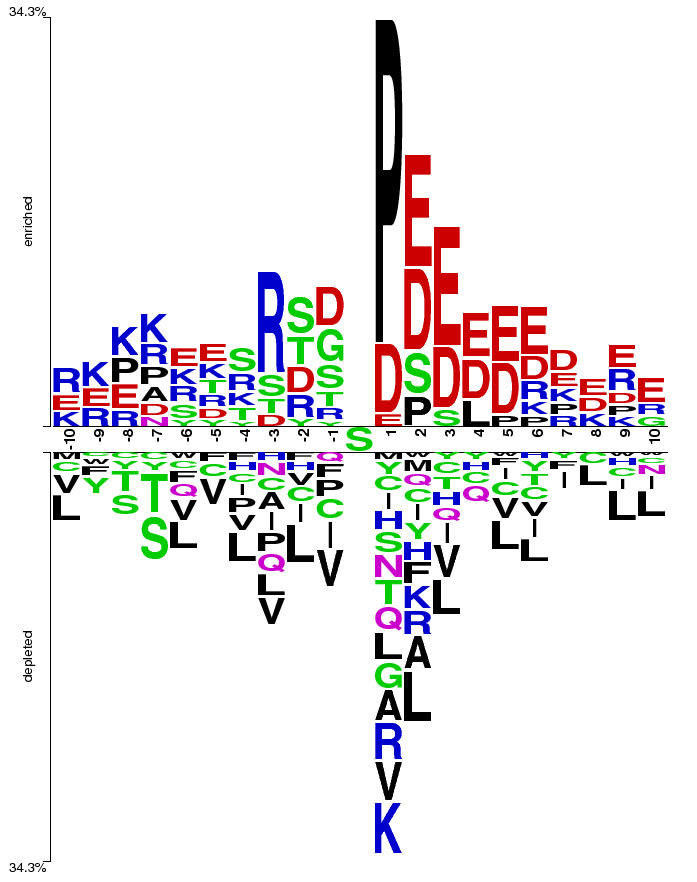
**

**Fig. G.** Two sample logo analysis of PSSs from the PS1D-70 dataset in cerebellum.

**
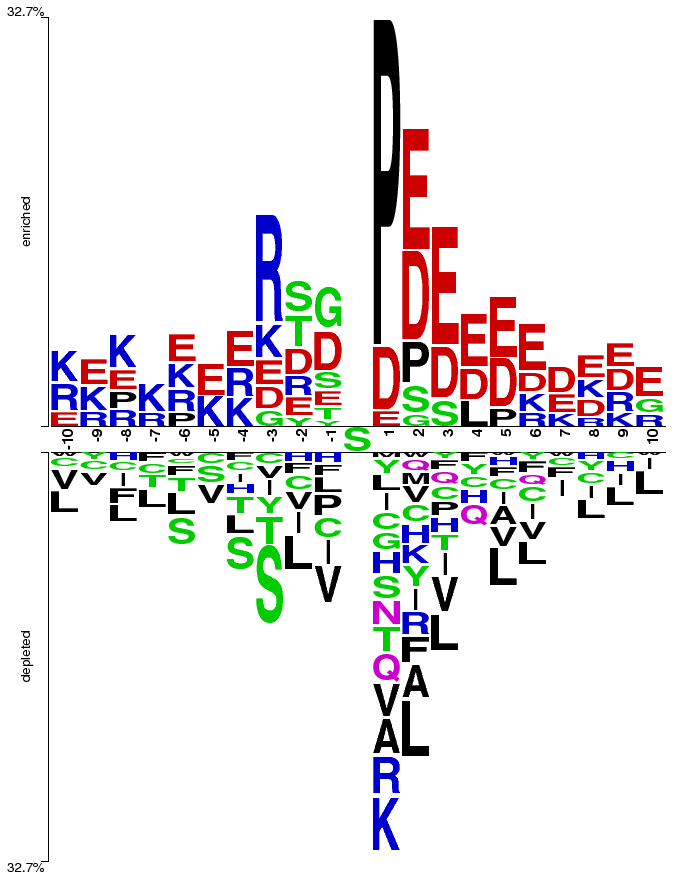
**

**Fig. H.** Two sample logo analysis of PSSs from the PS1D-70 dataset in cortex.


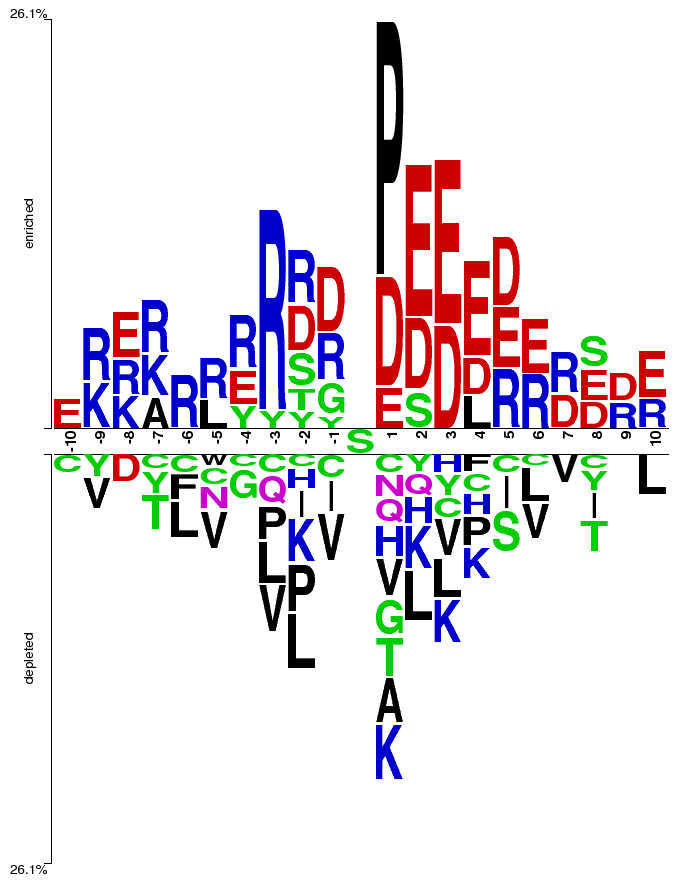


**Fig. I.** Two sample logo analysis of PSSs from the PS1D-70 dataset in heart.


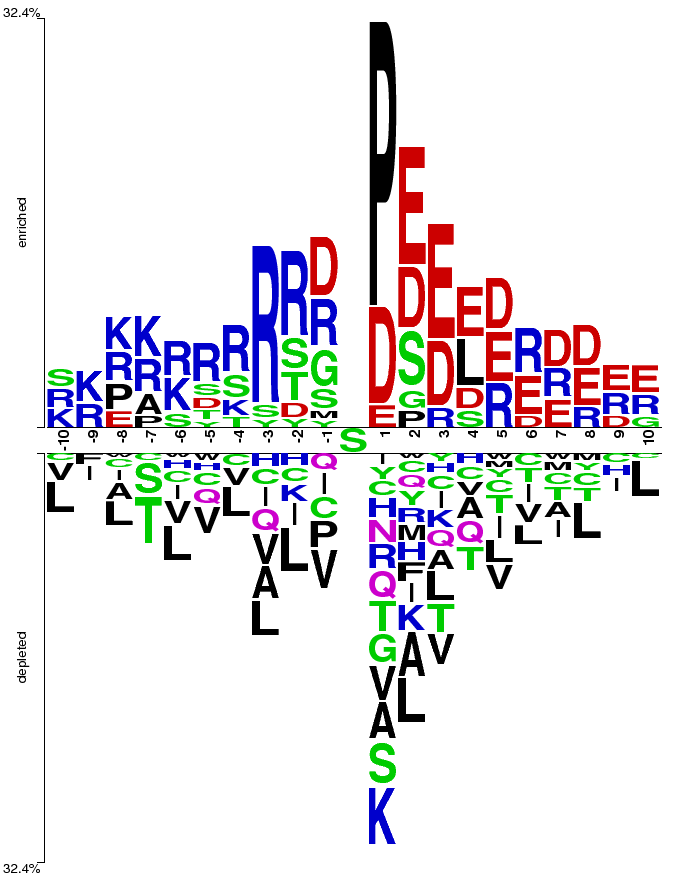


**Fig. J.** Two sample logo analysis of PSSs from the PS1D-70 dataset in intestine.


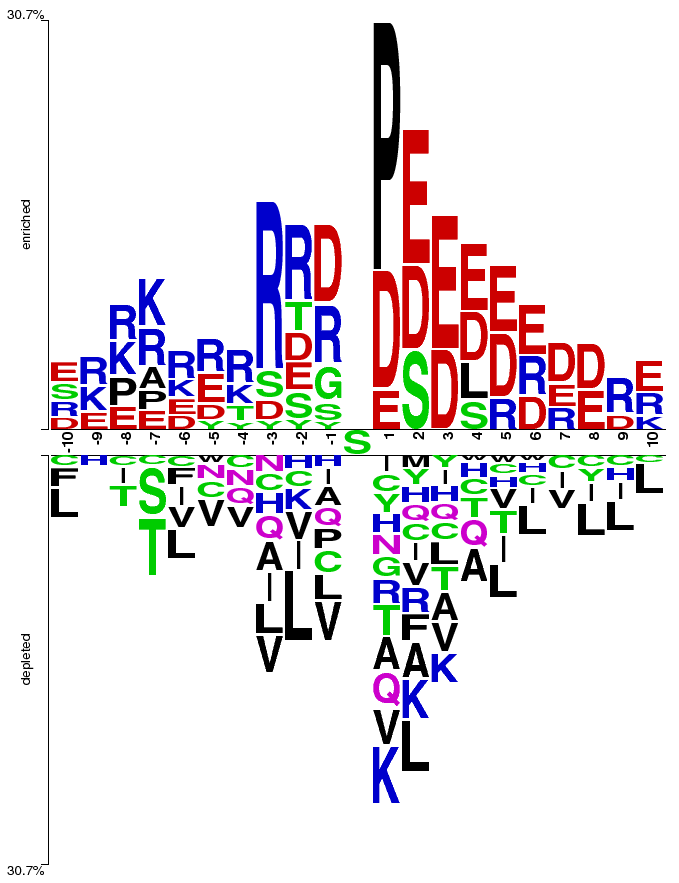


**Fig. K.** Two sample logo analysis of PSSs from the PS1D-70 dataset in kidney.


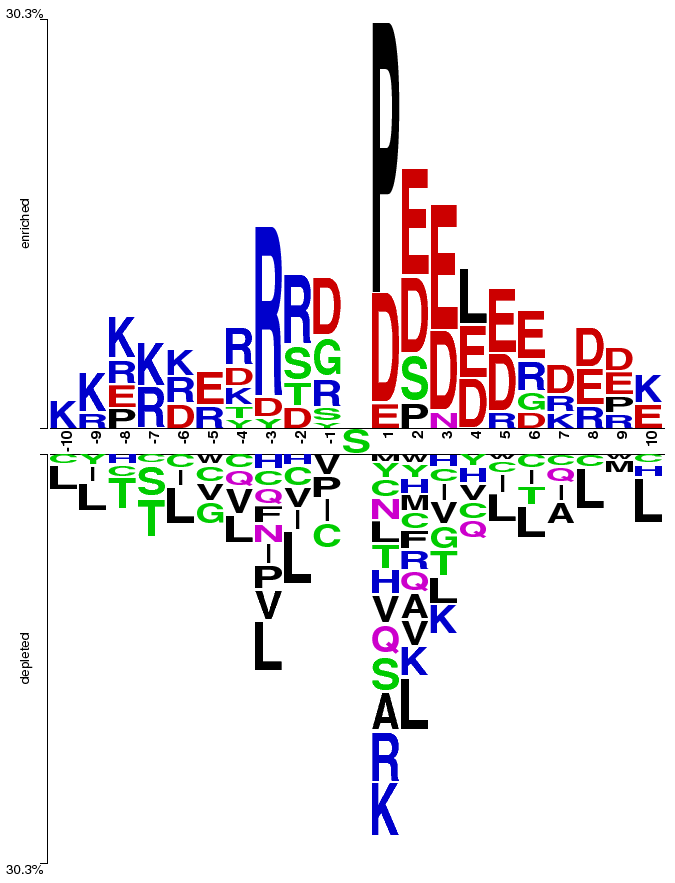


**Fig. L.** Two sample logo analysis of PSSs from the PS1D-70 dataset in liver.


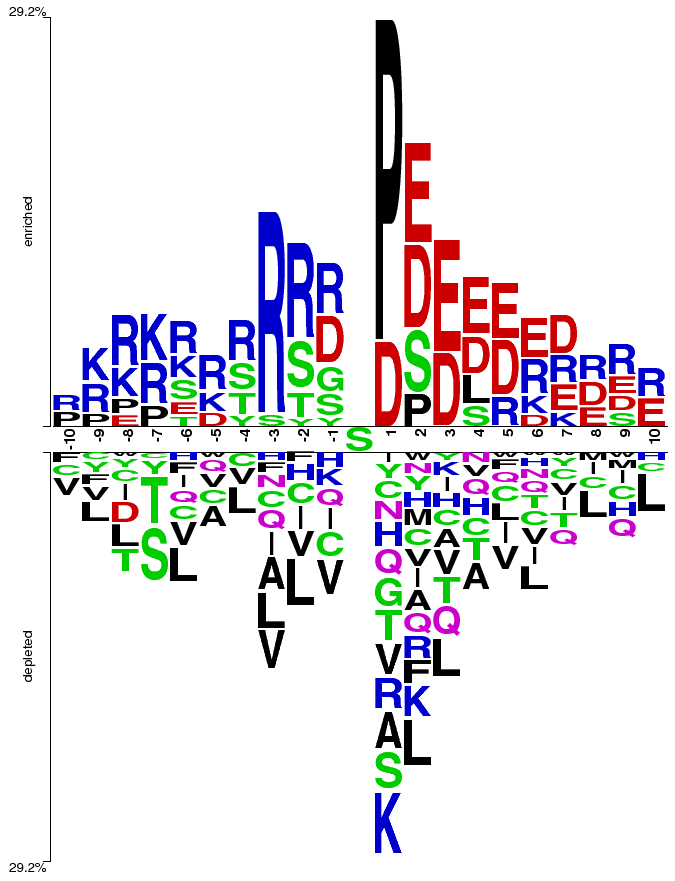


**Fig. M.** Two sample logo analysis of PSSs from the PS1D-70 dataset in lung.


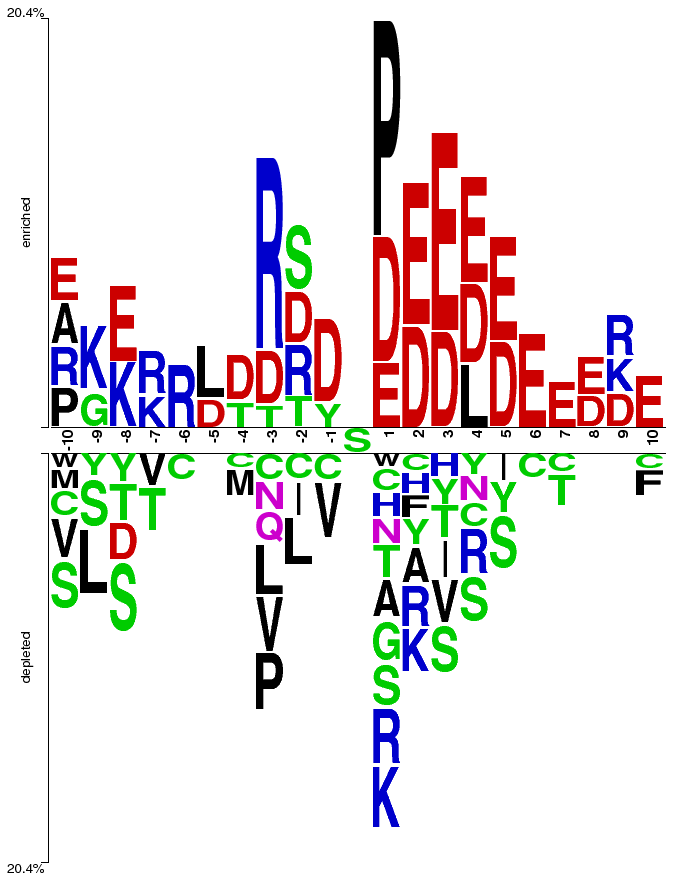


**Fig. N.** Two sample logo analysis of PSSs from the PS1D-70 dataset in muscle.


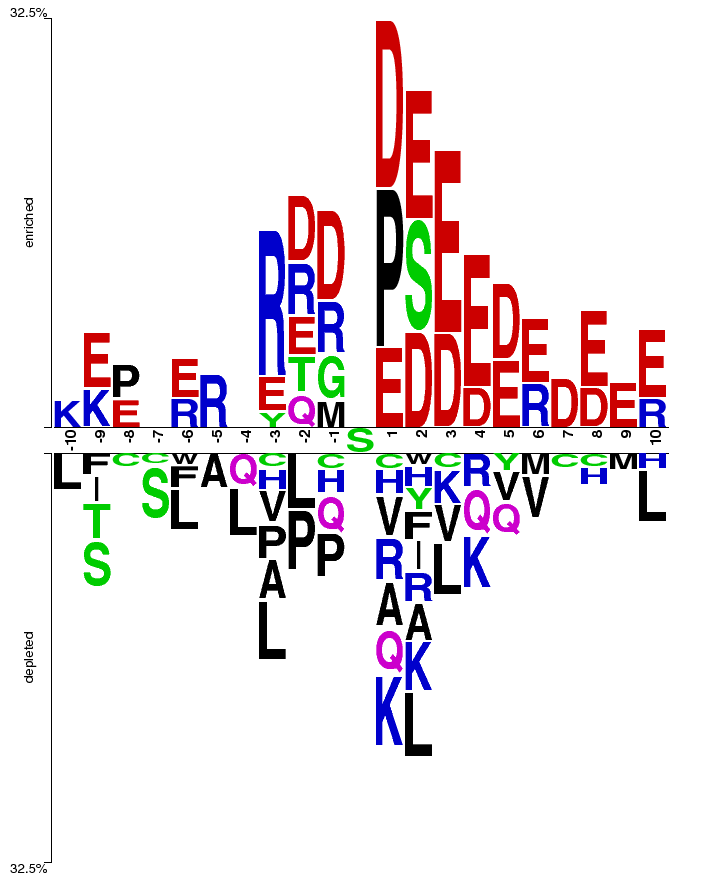


**Fig. O.** Two sample logo analysis of PSSs from the PS1D-70 dataset in pancreas.

**
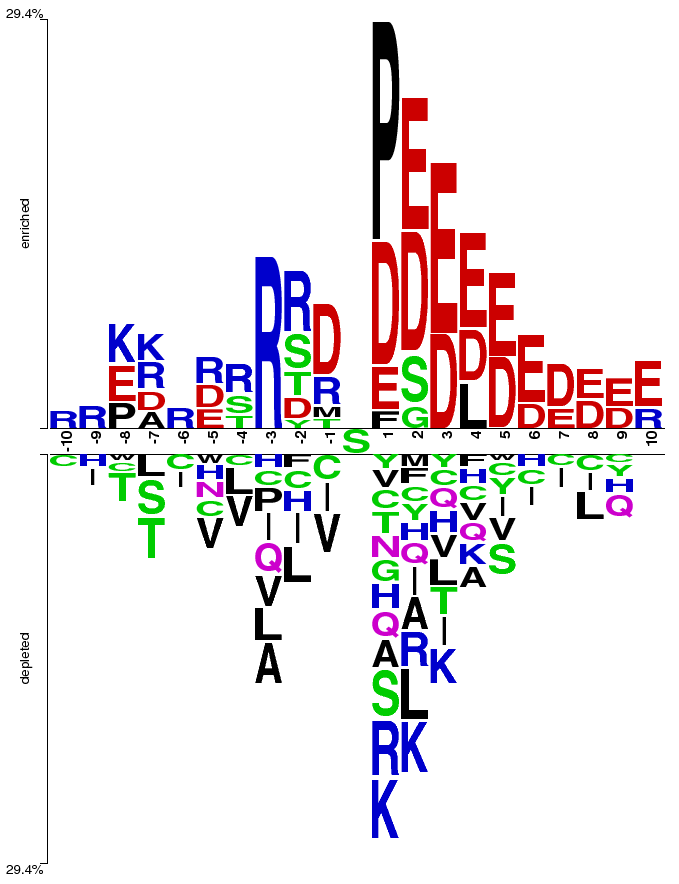
**

**Fig. P.** Two sample logo analysis of PSSs from the PS1D-70 dataset in perirenal fat.

**
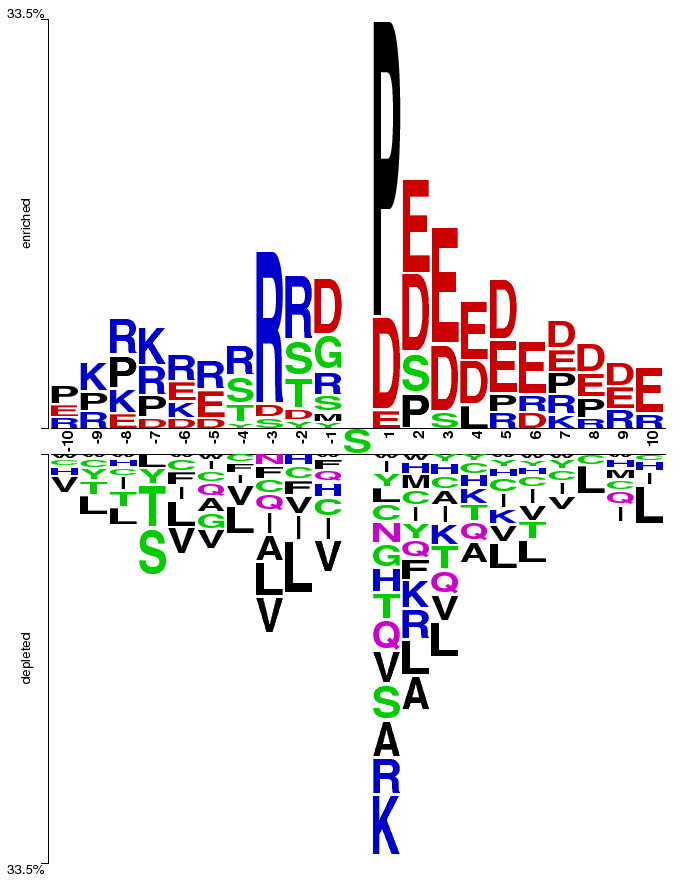
**

**Fig. Q.** Two sample logo analysis of PSSs from the PS1D-70 dataset in spleen.

**
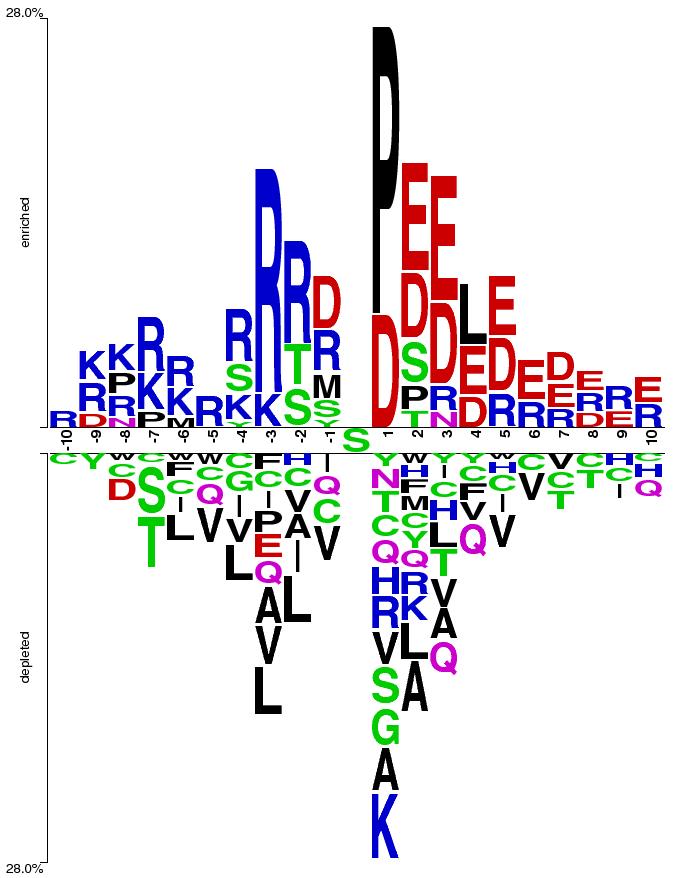
**

**Fig. R.** Two sample logo analysis of PSSs from the PS1D-70 dataset in stomach.

**
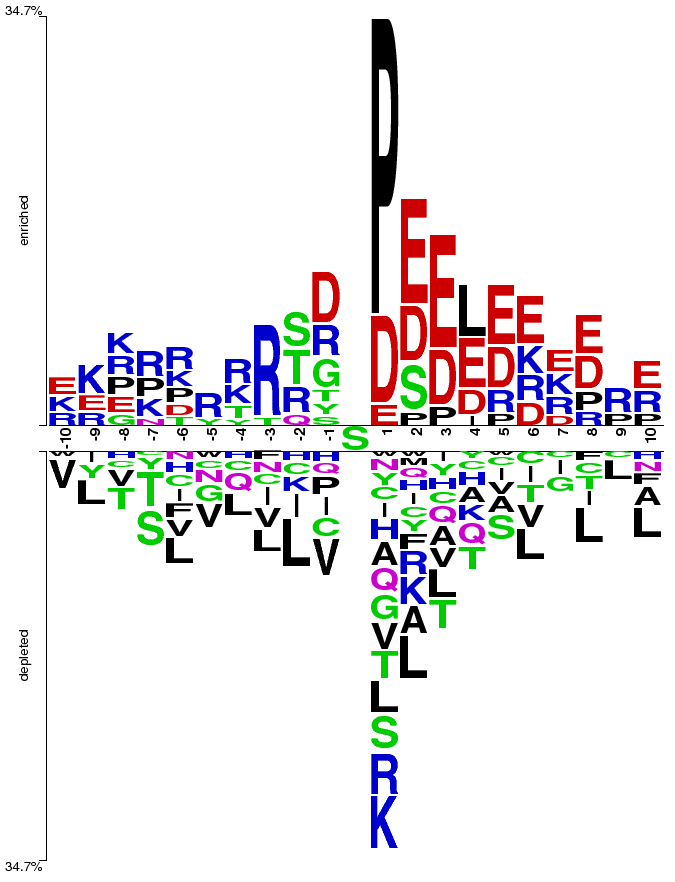
**

**Fig. S.** Two sample logo analysis of PSSs from the PS1D-70 dataset in testis.

**
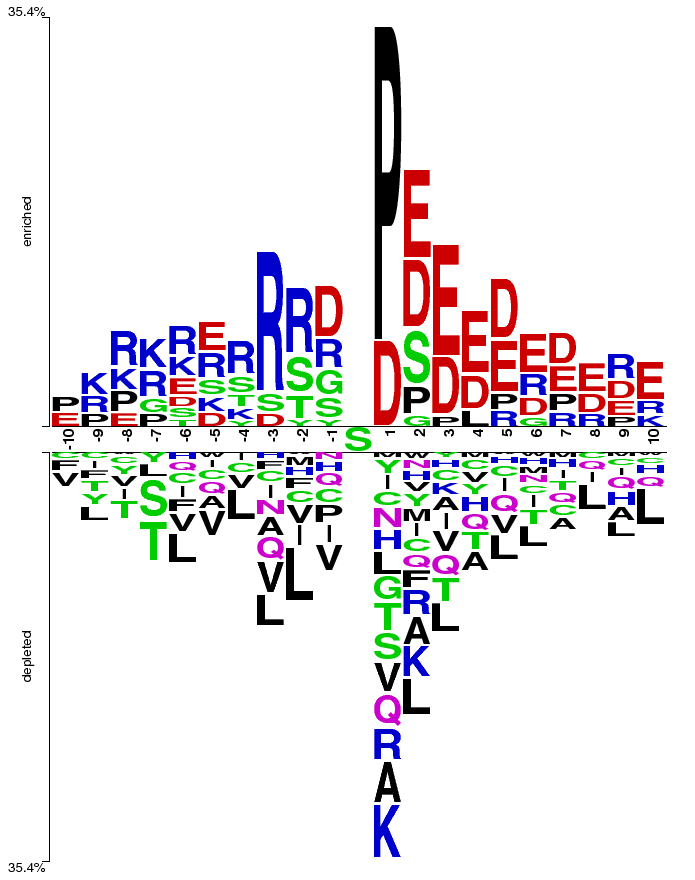
**

**Fig. T.** Two sample logo analysis of PSSs from the PS1D-70 dataset in thymus.

**
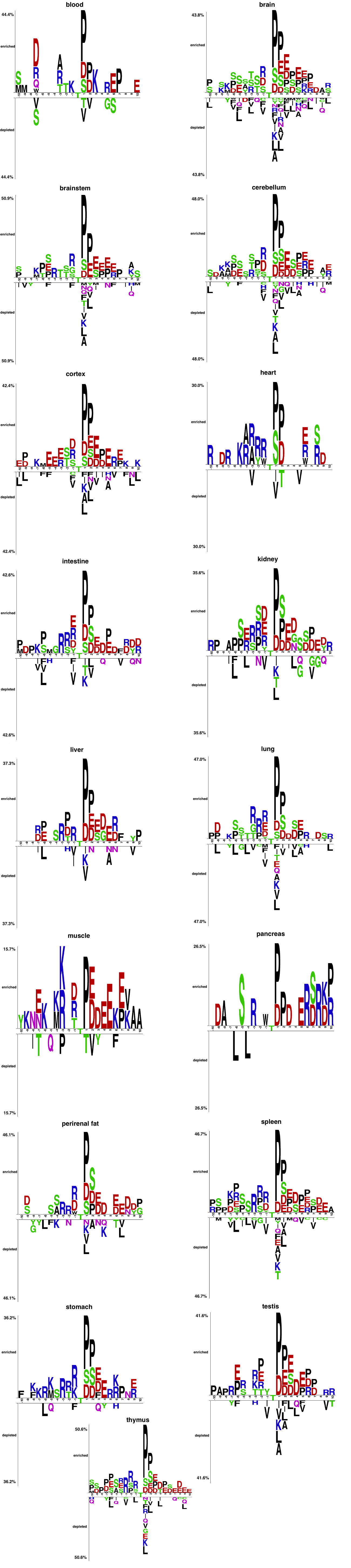
**

**Fig. U.** Two sample logo analysis of PTSs in different tissues in the PS1D-70 dataset.

**
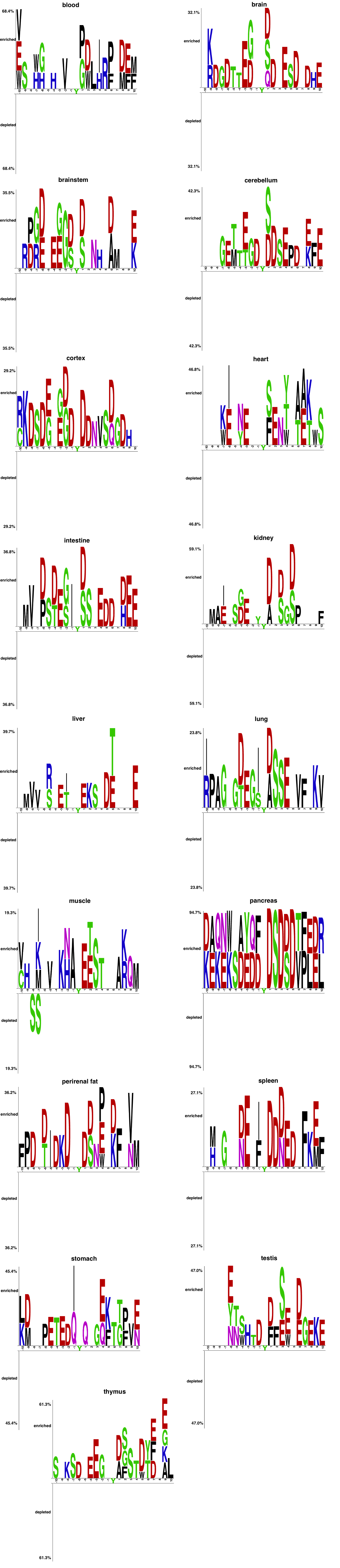
**

**Fig. V.** Two sample logo analysis of PYSs in different tissues in the PS1D-70 dataset.

**
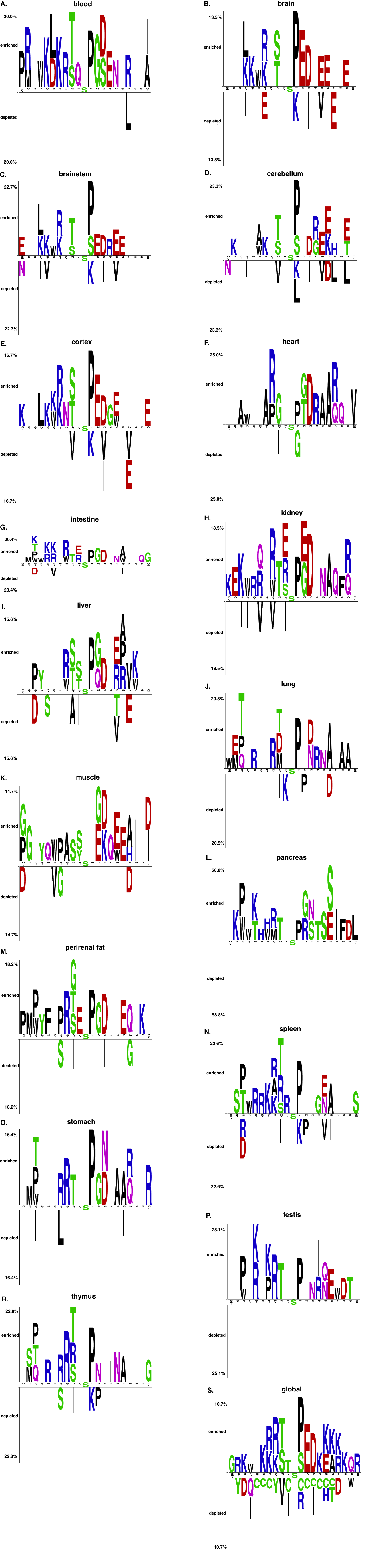
**

**Fig. W.** Two sample logo analysis of PSSs in different tissues in the PS3D-90 dataset.

**
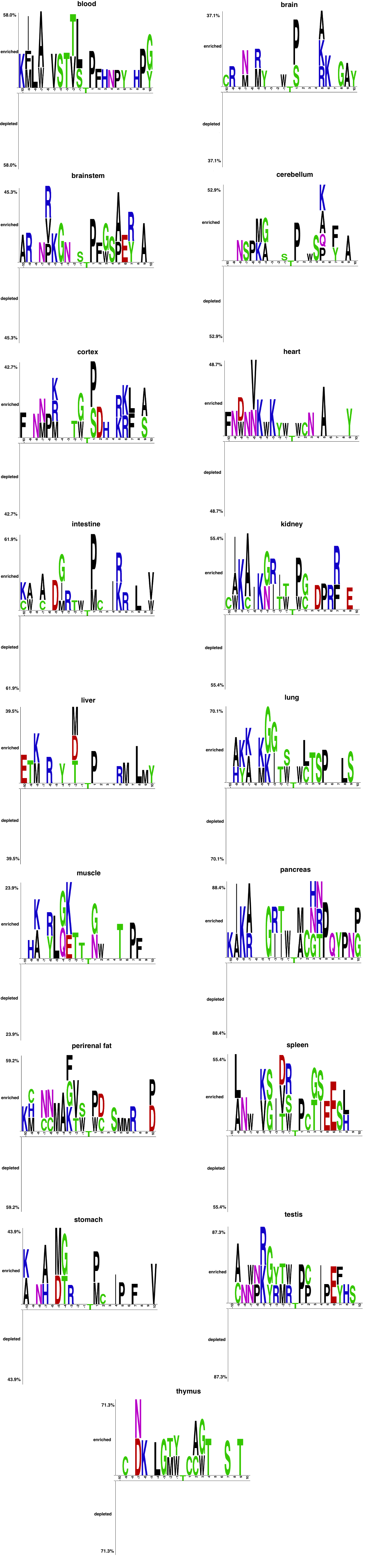
**

**Fig. X.** Two sample logo analysis of PTSs in different tissues in the PS3D-90 dataset.

**
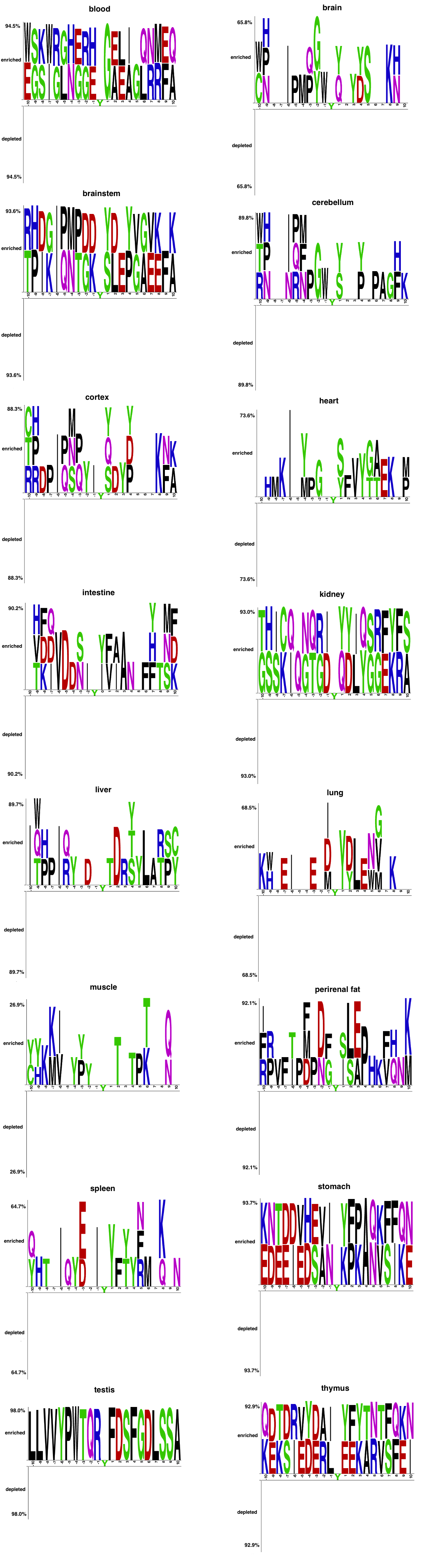
**

**Fig. Y.** Two sample logo analysis of PYSs in different tissues in the PS3D-90 dataset.


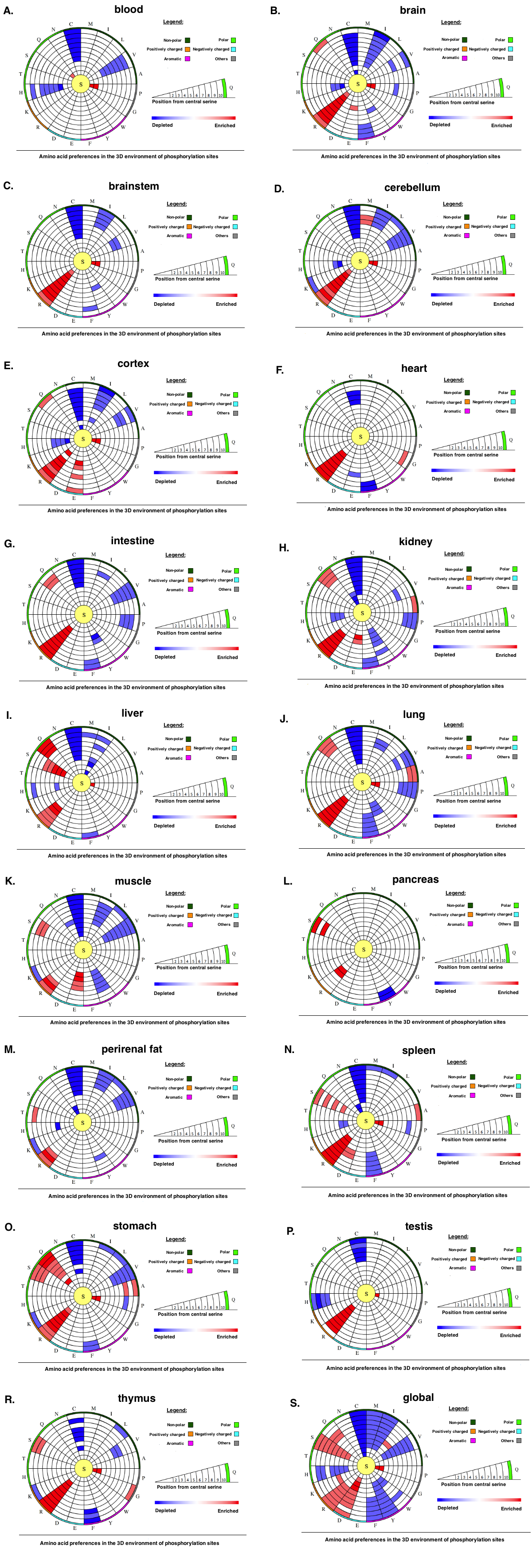


**Fig. Z.** 3D environment of PSSs from the PS3D-90 dataset in different tissues.


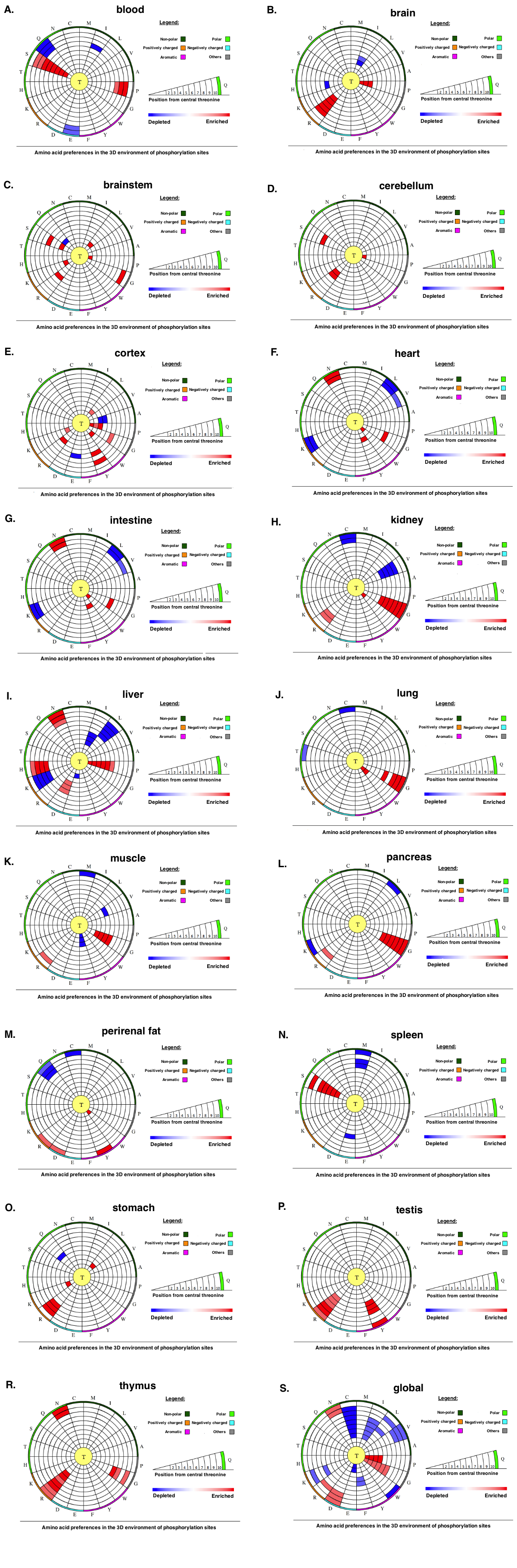


**Fig. AA.** 3D environment of PTSs from the PS3D-90 dataset in different tissues.


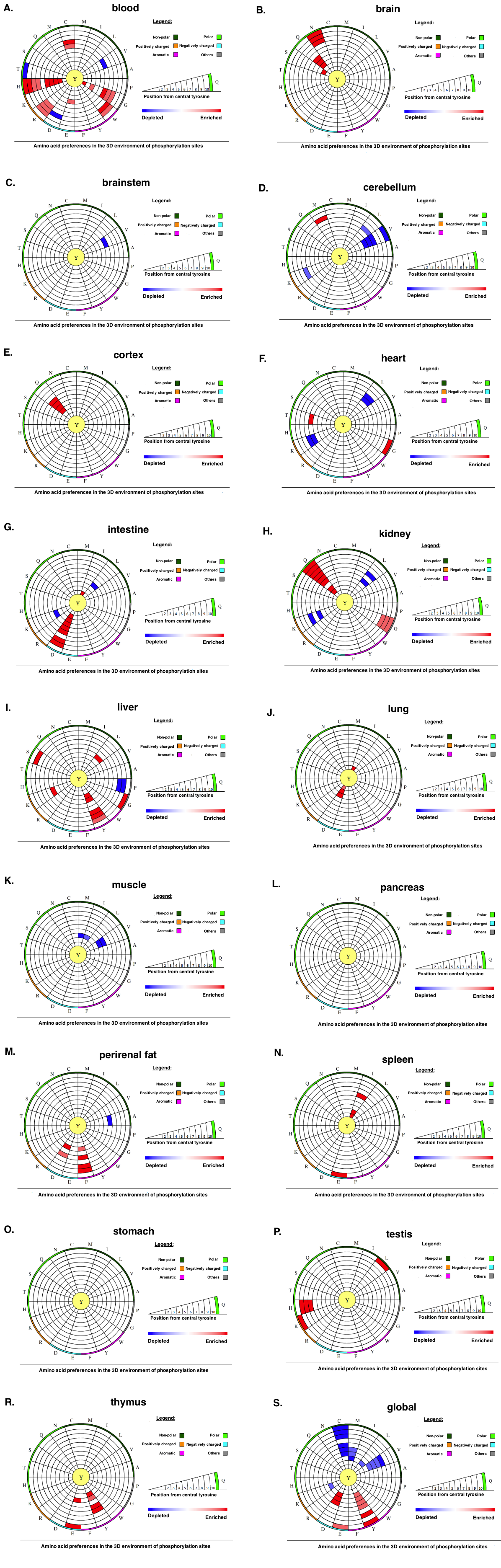


**Fig. AB.** 3D environment of PYSs from the PS3D-90 dataset in different tissues.


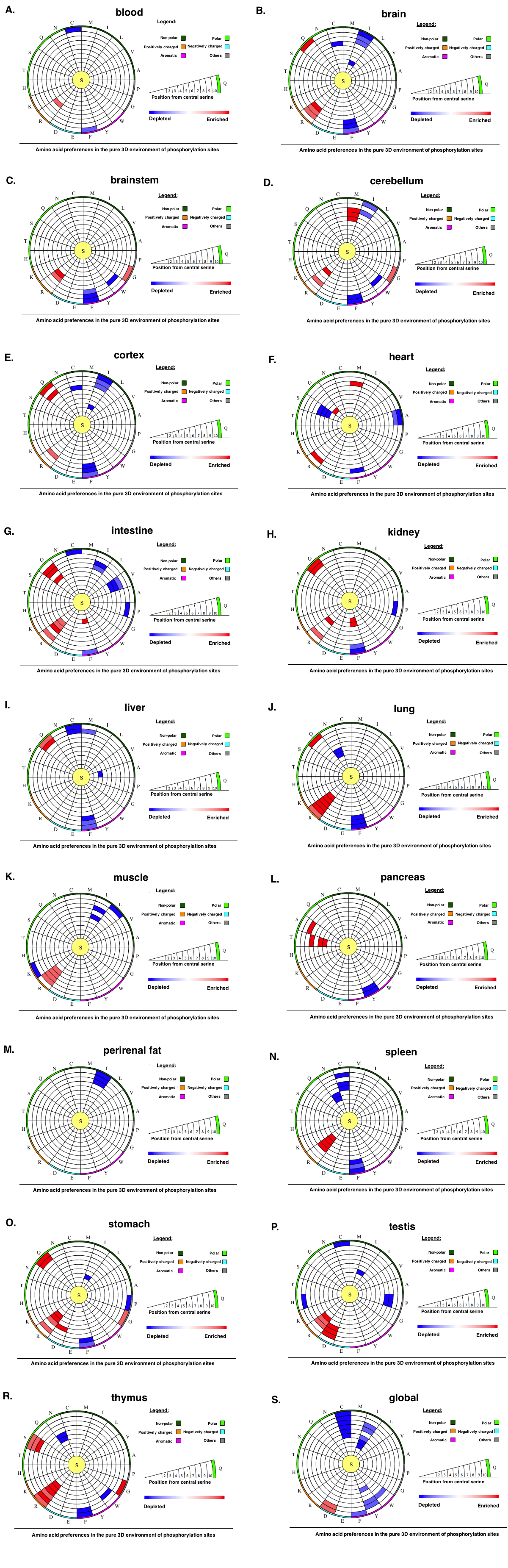


**Fig. AC.** Pure 3D environment of PSSs from the PS3D-90 dataset in different tissues.


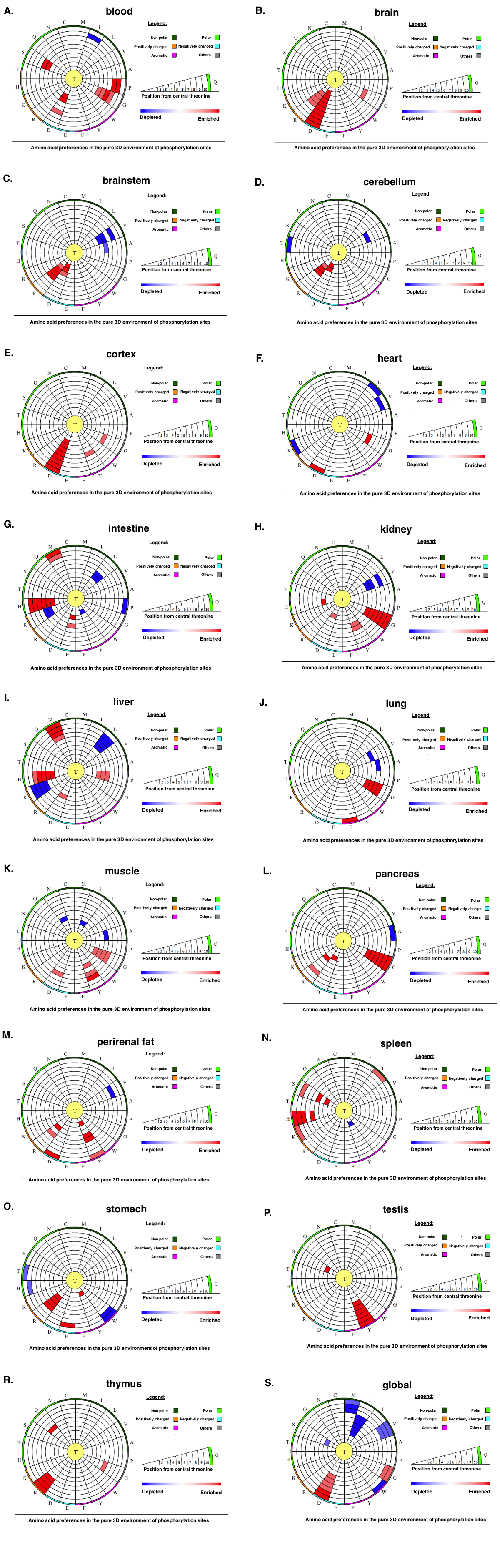


**Fig. AD.** Pure 3D environment of PTSs from the PS3D-90 dataset in different tissues.


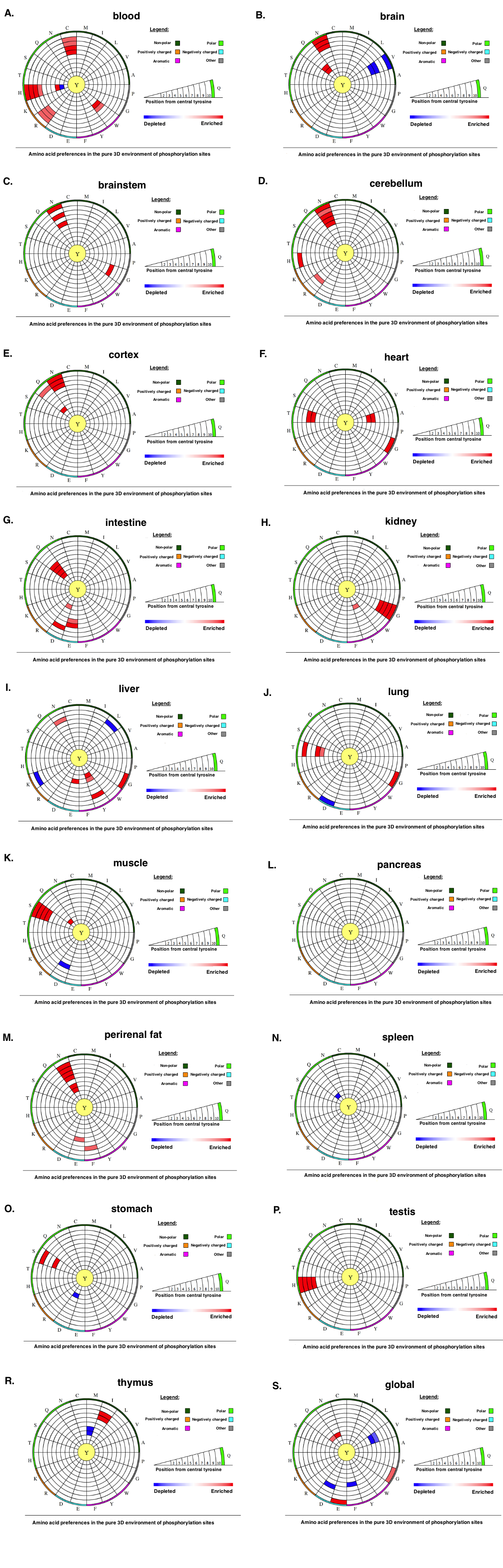


**Fig. AE.** Pure 3D environment of PYSs from the PS3D-90 dataset in different tissues.

**
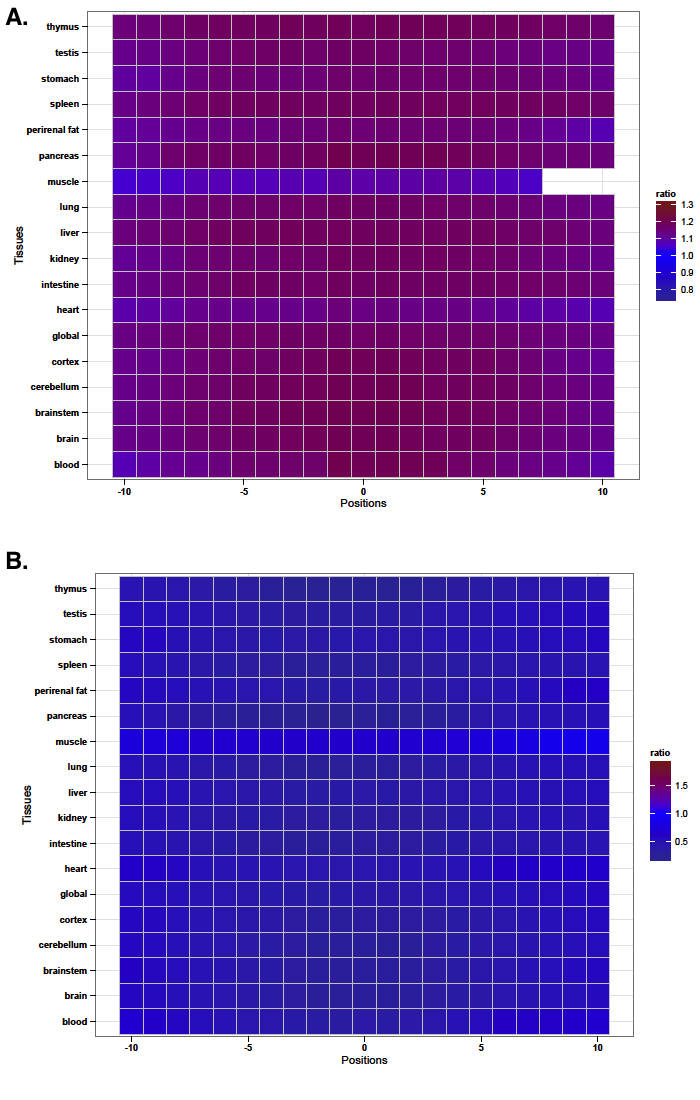
**

**Fig. AF.** Disorder region analysis of PSSs and the residues surrounding them from the PS1D-70 dataset in different tissues. **A.** Ratio represented with shades of blue and red shows the normalized number of PSSs found in disorder regions divided by the normalized number of non-PSSs found in disorder regions. Non-significant ratios (p-value > 0.05) are represented with white cells. **B.** Ratio represented with shades of blue and red shows the normalized number of PSSs found in ordered regions divided by the normalized number of non-PSSs found in ordered regions. Non-significant ratios (p-value > 0.05) are represented with white cells. **A** and **B** are not complement of each other, because some phosphorylation sites with unknown regions also exist.


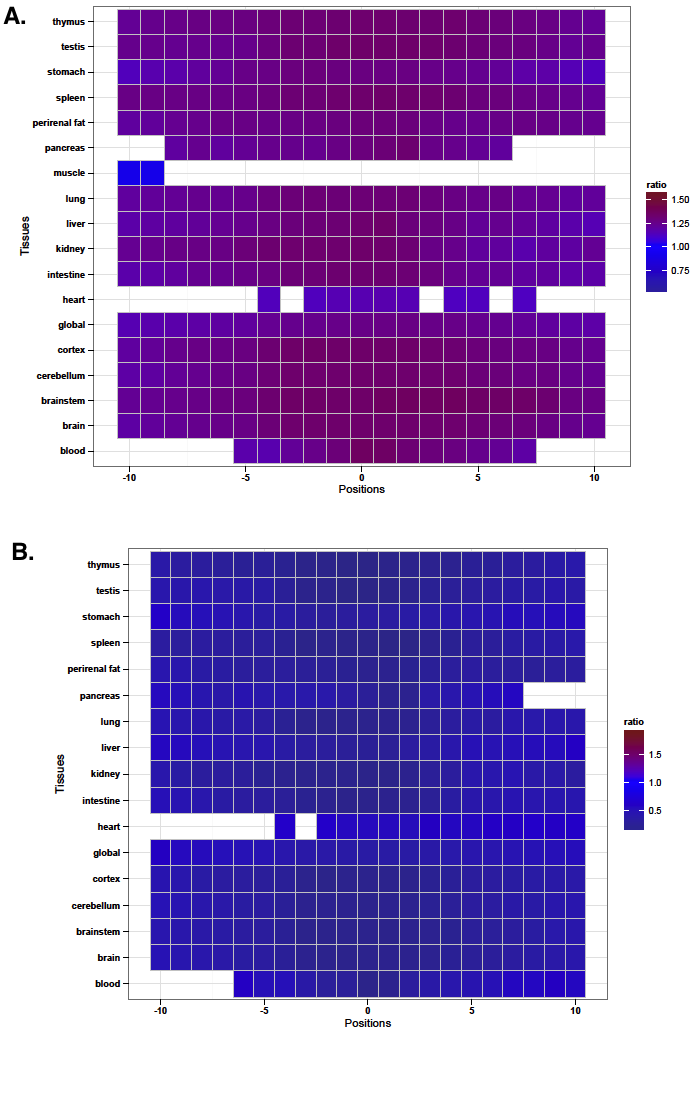


**Fig. AG.** Disorder region analysis of PTSs and the residues surrounding them from the PS1D-70 dataset in different tissues. **A.** Ratio represented with shades of blue and red shows the normalized number of PTSs found in disorder regions divided by the normalized number of non-PTSs found in disorder regions. Non-significant ratios (p-value > 0.05) are represented with white cells. **B.** Ratio represented with shades of blue and red shows the normalized number of PTSs found in ordered regions divided by the normalized number of non-PTSs found in ordered regions. Non-significant ratios (p-value > 0.05) are represented with white cells. **A** and **B** are not complement of each other, because some phosphorylation sites with unknown regions also exist.


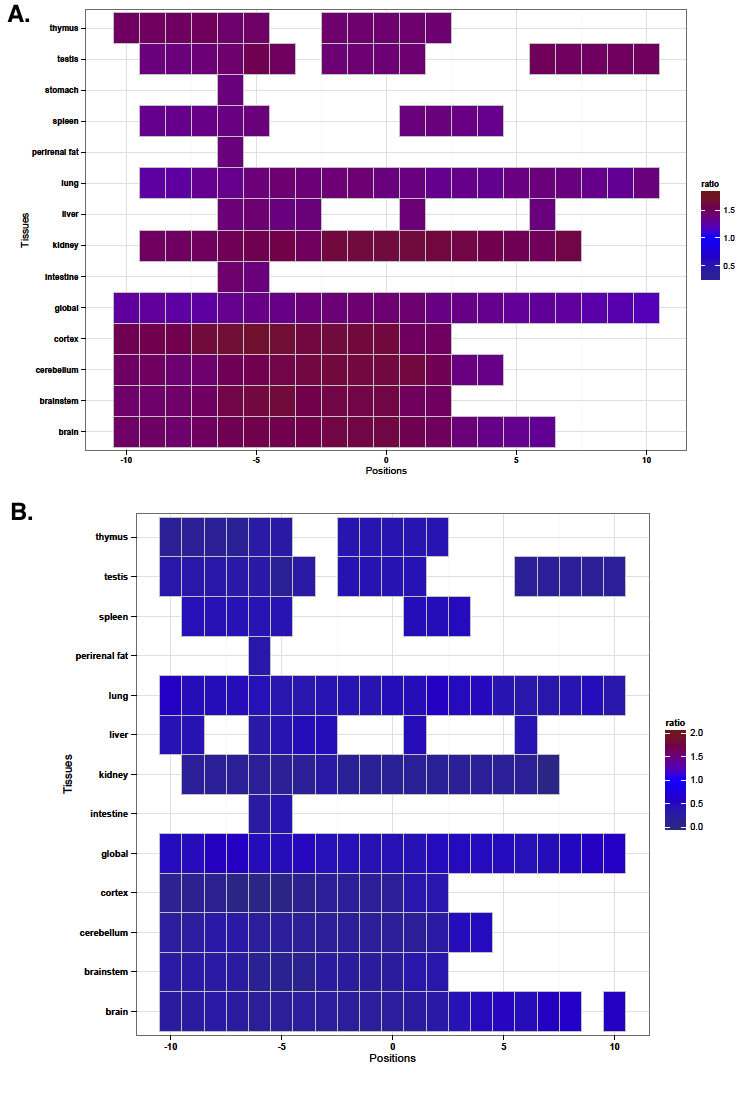


**Fig. AH.** Disorder region analysis of PYSs and the residues surrounding them from the PS1D-70 dataset in different tissues. **A.** Ratio represented with shades of blue and red shows the normalized number of PYSs found in disorder regions divided by the normalized number of non-PYSs found in disorder regions. Non-significant ratios (p-value > 0.05) are represented with white cells. **B.** Ratio represented with shades of blue and red shows the normalized number of PYSs found in ordered regions divided by the normalized number of non-PYSs found in ordered regions. Non-significant ratios (p-value > 0.05) are represented with white cells. **A** and **B** are not complement of each other, because some phosphorylation sites with unknown regions also exist.

**Fig. AI.** Secondary structure analysis of PSSs and the residues surrounding them from the PS3D-90 dataset in different tissues. Ratios represented with shades of blue and red show the normalized number of PSSs found in a particular secondary structure divided by the normalized number of non-PSSs found in the corresponding structure. Non-significant ratios (p-value > 0.05) are represented with white cells.

**Fig. AJ.** Secondary structure analysis of PTSs and the residues surrounding them from the PS3D-90 dataset in different tissues. Ratios represented with shades of blue and red show the normalized number of PTSs found in a particular secondary structure divided by the normalized number of non-PTSs found in the corresponding structure. Non-significant ratios (p-value > 0.05) are represented with white cells.

**Fig. AK.** KEGG pathway analysis of the threonine phosphorylated proteins from the PS1D-70 dataset. Pathways with a corrected p-value < 0.01 in each tissue are considered significant.

**Fig. AL.** KEGG pathway analysis of the tyrosine phosphorylated proteins from the PS1D-70 dataset. Pathways with a corrected p-value < 0.01 in each tissue are considered significant.

**Fig. AM.** SCOP *class* analysis of serine phosphorylation sites and the residues surrounding them in different tissues in the PS3D-90 dataset. Ratio represented with shades of blue shows the normalized number of PSSs found in a particular protein structural class divided by the normalized number of non-PSSs found in the corresponding class. Black circles represent significant p-values (p < 0.05).

**Fig. AN.** Global and tissue-specific occurrence of serine phosphorylated proteins from the PS3D-90 dataset in protein domains. Domains with a corrected p-value < 0.05 are considered significant.

**Fig. AO.** Global and tissue-specific occurrence of threonine phosphorylated proteins from the PS3D-90 dataset in protein domains. Domains with a corrected p-value < 0.05 are considered significant.

**Fig. AP.** Global and tissue-specific occurrence of tyrosine phosphorylated proteins from the PS3D-90 dataset in protein domains. Domains with a corrected p-value < 0.05 are considered significant.
