# Supplementary material for: Sequence- and Structure-Based Analysis of Tissue-Specific Phosphorylation Sites
Source: PLoS One. 2016 Jun 22;11(6):e0157896. doi: 10.1371/journal.pone.0157896 (PMC4917084; doi:10.1371/journal.pone.0157896)
Supplement: S1 Supporting Tables — (DOCX) [file pone.0157896.s004.docx]

**SUPPLEMENTARY TABLES**

| **Tissue** | **AA** | **Motif** | **Motif Score** |
| --- | --- | --- | --- |
| Blood | S | pS-P  pS-X-E  R-X-X-pS  pS-D-X-E | 16.00  14.09  11.21  15.39 |
|  | T | pT-P | 8.34 |
|  | Y | No motif |  |
| Global | S | R-pS-X-S-P  R-S-X-pS-P  pS-P-X-X-S-P  R-pS-X-S  S-P-pS  S-P-X-X-pS-P  S-X-X-X-pS-P  pS-X-X-X-S-P  pS-X-S-P  R-X-X-S-X-pS  R-X-X-pS-P  R-S-X-pS  pS-P-X-X-X-R  pS-D-E-E  pS-P-X-X-X-K  pS-E-X-E-X-D  pS-X-D-E-X-E  pS-D-D-E  pS-P-X-R  pS-D-X-E-D  pS-E-E-E  pS-D-X-E  pS-X-X-S-P  R-X-X-X-pS-P  R-X-X-pS-X-E  pS-S-P  R-X-pS-X-S  pS-P-X-X-X-X-X-X-X-X-R  pS-X-D-E  R-X-G-pS  S-P-X-pS  pS-D-E-D  pS-P-X-X-X-X-X-X-X-X-K  pS-P-X-X-X-X-X-X-X-X-E  pS-X-E-E  pS-X-X-D-X-X-E  pS-D-X-D-X-E  pS-X-X-X-X-D-X-E  R-X-X-pS-X-X-X-X-E  R-X-X-X-X-X-X-X-pS-P  E-E-X-X-X-X-X-X-X-pS  R-R-X-pS  S-P-X-X-pS-X-X-X-X-R  R-X-X-S-X-X-pS  pS-P-R  pS-X-X-X-X-E-E  pS-D-D-D  R-X-X-S-P-X-X-X-X-X-pS  R-X-X-S-X-X-X-X-X-pS  pS-P-X-X-X-X-X-X-X-R  pS-R-X-X-S  pS-X-D-D  pS-X-E-D  pS-P-X-X-R  D-X-D-pS  R-X-X-S-X-X-X-pS  pS-X-X-R-X-X-S  pS-X-X-X-X-X-R-X-X-S  R-R-X-X-pS  pS-X-D-X-D  R-X-X-X-X-X-X-X-X-X-pS-P  R-X-pS-S  S-P-X-X-X-X-X-X-X-pS-X-X-X-X-X-X-X-X-R  R-X-X-S-X-X-X-X-pS  D-D-X-X-X-X-X-pS  S-P-X-X-X-X-X-X-X-X-pS-X-X-X-X-X-X-X-R  pS-X-S-X-X-X-X-X-X-R  D-X-X-X-X-X-X-X-X-pS  R-X-X-S-X-X-X-X-X-X-pS-X-X-X-L  R-X-X-X-X-X-X-X-X-pS-P  pS-X-X-X-X-R-X-X-S  R-X-pS-P  pS-X-S-X-D  pS-P  pS-X-X-X-X-X-X-X-X-X-D  pS-X-X-X-X-X-X-X-D-X-E  pS-X-X-X-X-X-X-X-X-X-R  R-X-X-X-pS-X-X-X-X-X-X-S  P-X-X-X-X-X-X-X-X-X-pS-X-X-X-X-X-X-R-X-X-S  R-X-X-pS  S-X-X-X-X-X-X-X-X-X-pS-X-X-X-X-X-X-X-R  pS-X-D-X-X-D  R-X-pS  pS-X-X-D  pS-X-X-E-E  pS-X-X-X-X-X-X-X-X-R  R-X-S-X-pS  D-X-X-X-X-X-X-X-X-X-pS  S-X-X-X-X-X-X-X-pS-X-X-X-D  R-X-X-X-X-X-X-X-pS  pS-X-X-X-X-X-X-X-E-X-E  R-X-X-X-X-X-X-X-X-X-pS  pS-X-X-R  D-X-X-X-X-X-X-pS  pS-X-X-X-X-X-X-D  R-X-X-X-X-X-X-pS  pS-X-X-X-X-X-R  R-X-X-X-X-pS  K-X-pS  D-X-X-X-pS  pS-X-X-X-X-X-X-R  pS-X-X-K  K-X-X-X-X-X-X-X-pS  pS-R  pS-D  pS-X-X-X-X-X-X-X-R  pS-X-X-E  pS-X-R  pS-X-X-X-X-K  pS-X-X-S  P-X-X-X-X-X-X-pS  pS-X-X-X-R  pS-X-X-G | 42.91  42.66  47.48  32.00  32.00  44.63  32.00  32.00  32.00  32.00  29.14  32.00  29.66  42.17  29.59  41.24  41.09  42.37  27.24  38.52  40.94  32.00  32.00  23.53  32.00  32.00  32.00  24.71  32.00  27.54  32.00  36.13  24.11  30.68  25.67  25.24  32.83  28.13  26.21  23.89  26.96  28.19  38.18  32.00  25.82  23.71  37.75  31.03  29.73  25.89  32.00  25.14  23.76  29.37  26.27  27.96  30.42  32.00  22.50  24.57  23.31  25.08  36.27  25.99  23.79  36.22  22.55  16.00  29.67  22.04  32.00  22.31  22.76  16.00  16.00  23.40  16.00  23.60  35.57  16.00  23.09  26.06  16.00  16.00  25.76  15.65  22.64  13.87  21.07  13.28  19.05  12.49  14.65  11.28  12.54  11.29  10.74  10.75  10.65  9.84  9.66  9.74  9.77  8.13  8.62  7.88  8.37  8.27  8.36  8.02  6.02  6.10  6.32 |
|  | T | pT-P-P  pT-P-E  pT-X-S-P  pT-S-P  pT-P  S-X-pT  pT-X-X-X-X-E  pT-X-S  pT-X-D  S-X-X-pT  pT-D-X-E | 32.00  23.30  24.71  32.00  16.00  15.65  13.33  8.42  9.06  7.56  12.85 |
|  | Y | pY-X-X-X-E  pY-D  pY-X-X-S | 6.76  6.78  6.03 |
| Brain | S | **pS-P-E***  R-X-X-pS-P  **E-X-X-X-X-X-X-X-X-pS-P***  **pS-P-X-X-E***  pS-P  pS-D-D-E  pS-D-X-E  R-X-X-pS  pS-X-E-D  pS-E-X-E  pS-X-E  pS-X-D-D  R-X-pS  pS-X-D  **pS-X-S-P**  **E-X-X-X-X-pS***  D-X-X-X-X-pS  R-X-X-X-X-X-X-pS  **pS-X-X-X-X-X-X-X-X-D***  pS-X-X-X-X-X-X-X-X-R  **pS-X-X-X-X-X-X-X-X-E***  R-X-X-X-X-X-X-X-X-pS  **E-X-X-X-X-X-X-X-X-pS***  pS-X-X-X-X-E  pS-X-X-X-X-R  R-X-X-X-X-X-X-X-pS  pS-X-X-X-X-X-R  R-X-X-X-X-pS  R-X-X-X-X-X-X-X-X-X-pS  **pS-X-X-X-X-X-X-X-X-K***  pS-X-X-X-R  **K-X-pS**  E-X-X-X-X-X-pS  pS-X-X-E  E-X-X-X-X-X-X-X-pS | 24.35  23.65  23.69  22.98  16.00  44.19  30.32  16.00  22.36  30.18  16.00  25.74  15.95  13.97  28.00  13.64  11.67  11.57  11.61  10.42  10.41  9.81  8.35  11.02  8.34  7.64  8.94  7.36  6.26  6.05  7.50  6.40  6.41  6.12  6.06 |
|  | T | pT-P-P  pT-P  **pT-S-P**  **pT-X-X-X-X-E** | 25.98  16.00  20.41  6.94 |
|  | Y | No motif |  |
| Heart | S | R-X-X-pS-P  pS-P-X-X-X-R  pS-D-E-E  R-X-X-pS  pS-P  **pS-X-D-E**  R-pS  pS-D-X-D  pS-E-X-E  pS-X-X-X-X-X-X-X-X-R  R-X-X-X-X-X-X-X-X-pS  E-pS | 26.15  22.71  38.96  16.00  16.00  22.41  11.21  17.52  14.14  6.95  6.29  6.68 |
|  | T | No motif |  |
|  | Y | No motif |  |
| Intestine | S | R-X-X-pS-P  pS-P-X-X-X-X-R  **R-X-X-X-X-X-X-X-X-pS-P**  pS-D-D-E  **R-R-X-X-pS**  pS-D-E-E  pS-P-X-X-X-R  pS-D-X-E  R-X-X-X-pS-P  R-X-X-pS  pS-P  R-pS-X-S  pS-D-X-D  pS-E-X-E  R-X-X-X-X-X-X-X-X-X-pS  pS-X-X-R  R-X-pS  pS-X-X-X-X-X-X-X-X-X-R  pS-X-X-X-X-X-X-X-R  pS-R  pS-X-D  pS-X-X-X-R  R-X-X-X-X-X-X-X-pS  pS-X-X-X-X-R  E-X-X-X-X-X-X-X-pS  pS-X-X-X-X-X-X-E  R-X-X-X-X-X-X-pS  R-X-X-X-pS  R-pS  R-X-X-X-X-X-pS  pS-X-X-X-X-X-X-R | 31.26  30.31  24.63  41.11  22.15  40.90  23.47  29.48  22.17  16.00  16.00  22.02  30.72  32.00  16.00  16.00  16.00  14.29  12.08  12.38  11.79  10.46  11.63  10.95  9.48  8.77  8.31  8.50  6.89  6.17  6.35 |
|  | T | pT-P-P  pT-P | 22.82  14.91 |
|  | Y | No motif |  |
| Kidney | S | R-X-X-pS-P  pS-P-X-X-X-X-R  pS-D-D-E  pS-D-E-E  pS-P  R-R-X-pS  pS-E-X-E  **R-X-R-X-X-pS***  R-pS-X-S  pS-X-E-D  **D-pS-D***  pS-X-D-D  R-X-X-pS  pS-X-X-X-X-X-X-X-D  R-X-pS  pS-X-X-X-X-X-X-X-X-X-R  D-X-X-X-X-pS  R-X-X-X-pS  R-X-X-X-X-X-X-pS  D-X-X-X-X-X-X-pS  R-X-X-X-X-X-X-X-pS  pS-X-X-X-R  E-X-X-X-X-X-X-X-pS  pS-X-X-X-X-E  R-X-X-X-X-X-X-X-X-X-pS  pS-X-X-X-X-R-X-X-S  **D-X-pS***  pS-X-X-X-X-X-X-X-X-R | 30.61  29.10  41.79  40.00  16.00  24.51  29.30  22.03  22.06  25.75  30.11  24.74  13.99  13.67  11.95  11.49  9.93  8.80  9.88  7.51  8.24  9.50  6.72  7.22  7.45  13.06  6.06  6.53 |
|  | T | pT-P | 16.00 |
|  | Y | No motif |  |
| Liver | S | pS-P-X-X-X-X-R  pS-P  pS-D-D-E  R-X-X-pS  pS-D-X-E  **pS-X-X-X-X-D***  pS-X-X-X-X-R  R-X-pS  pS-X-X-X-X-X-X-X-D  R-pS  E-pS  pS-X-X-X-X-X-X-X-R  pS-X-X-X-X-X-X-X-X-X-R  R-X-X-X-X-X-X-X-X-pS | 22.35  16.00  40.42  16.00  32.00  16.00  10.45  9.72  10.04  8.52  7.30  7.12  6.63  6.30 |
|  | T | pT-P | 16.00 |
|  | Y | No motif |  |
| Muscle | S | R-X-X-pS  pS-P  pS-D-X-E  E-X-X-X-X-X-X-X-pS  **pS-X-X-D**  pS-X-X-X-X-E | 16.00  12.55  29.26  8.56  7.53  6.61 |
|  | T | pT-P | 7.78 |
|  | Y | No motif |  |
| Lung | S | pS-P-X-X-X-X-R  R-X-X-pS-P  pS-P-X-X-X-R  **pS-P-X-X-X-X-X-X-X-X-R**  R-R-X-pS  pS-D-D-E  R-X-X-X-pS-P  pS-D-E-E  **R-X-X-pS-X-E**  pS-P-X-X-K  pS-D-X-E  R-pS-X-S  pS-P  R-X-X-pS  R-X-pS  pS-D-X-D  pS-X-X-X-X-X-X-X-X-R  R-X-X-S-X-pS  R-X-X-X-X-X-X-X-pS  pS-X-X-X-X-R-X-X-S  pS-X-X-X-X-X-X-X-X-X-R  R-X-X-X-pS  pS-E-X-E  pS-X-X-X-X-X-R  R-X-X-X-X-X-X-pS  R-pS  pS-X-X-R  pS-X-X-X-X-X-X-E  R-X-X-X-X-X-X-X-X-pS  pS-X-X-X-R  R-X-X-X-X-X-X-X-X-X-pS  pS-R  pS-X-X-X-X-X-X-X-R  **D-pS***  **K-X-X-pS***  R-X-X-X-X-X-pS  E-X-X-pS  pS-X-X-X-X-R  **pS-X-X-X-X-X-X-D**  **pS-X-X-X-X-X-X-X-K*** | 32.00  28.55  24.74  22.97  25.01  40.92  24.26  38.79  22.52  23.01  29.48  24.63  16.00  16.00  16.00  32.00  16.00  23.45  16.00  22.71  15.48  16.00  30.53  13.73  14.75  10.78  11.79  10.92  11.27  10.98  12.40  10.51  10.52  9.36  10.49  9.23  8.28  8.48  7.13  6.44 |
|  | T | pT-P-P  pT-P | 22.72  16.00 |
|  | Y | No motif |  |
| Pancreas | S | pS-D-X-E  R-X-X-pS  **pS-E-E***  pS-X-D-D  pS-P  pS-X-X-E  D-X-X-X-X-pS | 29.53  13.67  20.99  15.45  8.98  8.18  7.00 |
|  | T | No motif |  |
|  | Y | No motif |  |
| Perirenal fat | S | pS-P  pS-D-D-E  pS-D-X-E  R-X-X-pS  pS-X-E  pS-X-D-D  D-X-X-X-X-X-X-pS  R-pS  pS-X-D  pS-X-X-R  E-X-X-X-X-X-X-X-pS  R-X-X-X-X-X-X-X-X-pS  pS-X-X-E | 16.00  40.21  31.05  16.00  13.36  21.17  10.15  10.86  8.77  7.30  7.08  8.04  6.03 |
|  | T | pT-P | 16.00 |
|  | Y | No motif |  |
| Spleen | S | R-X-X-pS-P  **pS-P-X-X-X-X-X-R***  **P-X-pS-P***  pS-P  pS-D-D-E  pS-D-E-E  R-R-X-pS  pS-E-X-E-X-D  pS-D-X-E  R-X-X-pS  pS-E-X-E  pS-D-X-D  R-pS  pS-X-X-X-X-X-X-R  pS-X-D  pS-X-X-R  **R-X-X-S-X-X-pS**  R-X-pS  **E-X-E-X-X-X-X-X-pS***  **pS-X-X-X-X-X-E***  pS-X-X-X-R  pS-R  R-X-X-X-X-X-X-X-pS  pS-X-X-X-X-X-R  pS-X-X-X-X-X-X-X-X-X-R  E-X-X-pS  **pS-X-X-X-X-X-X-X-X-X-E***  pS-X-X-X-X-X-X-X-R  R-X-X-X-pS  E-X-X-X-X-X-pS  E-X-X-X-X-X-X-X-pS | 26.33  25.31  23.39  16.00  45.40  38.28  25.72  38.08  30.72  16.00  31.65  32.00  16.00  14.65  13.74  13.28  17.85  10.67  18.65  9.81  10.24  10.12  10.28  10.02  9.79  8.19  8.51  7.41  9.24  6.65  7.24 |
|  | T | pT-P-P  pT-P  pT-D | 25.31  16.00  6.28 |
|  | Y | No motif |  |
| Stomach | S | R-X-X-pS-P  R-X-X-X-pS-P  **R-X-X-pS-X-X-D***  pS-P-X-X-X-R  R-X-X-pS  **K-X-X-X-X-X-pS-P***  pS-D-E-E  pS-P  pS-D-D-E  R-X-pS  pS-X-X-X-X-X-X-X-X-R  R-pS  pS-D-X-D  pS-X-X-X-X-R  R-X-X-S-X-pS  R-X-X-X-X-X-X-pS  pS-X-X-R  pS-D-X-E  pS-X-X-X-X-X-X-X-X-X-R  R-X-X-X-X-X-X-X-X-pS  pS-X-X-X-X-X-R  R-X-X-X-X-X-X-X-pS  pS-X-X-X-R  R-X-X-X-X-X-X-X-X-X-pS | 26.03  24.24  22.47  23.53  16.00  22.31  38.25  16.00  39.91  16.00  14.95  12.52  21.24  11.93  18.87  10.69  9.79  18.71  8.20  8.60  7.32  7.93  7.87  6.57 |
|  | T | pT-P | 16.00 |
|  | Y | No motif |  |
| Testis | S | R-X-X-pS-P  pS-P-X-X-X-X-R  **pS-P-X-X-X-X-K***  pS-D-D-E  pS-P  pS-D-X-E  R-X-X-pS  pS-E-X-E  pS-D-X-D  **pS-X-E-X-L***  R-pS  pS-X-X-X-X-X-X-X-X-X-R  **pS-X-X-X-X-X-X-X-X-X-D**  pS-X-X-X-X-X-X-R  pS-X-X-R  R-X-X-X-X-X-X-pS  **K-X-X-pS-X-X-X-X-X-X-X-X-X-E***  pS-X-X-X-X-E  R-X-X-X-X-pS | 26.22  24.33  22.92  38.49  16.00  32.00  16.00  32.00  26.98  19.10  11.41  10.30  9.05  9.06  8.16  8.70  15.87  7.29  6.42 |
|  | T | pT-P-P  pT-P  pT-D | 26.54  16.00  7.64 |
|  | Y | No motif |  |
| Thymus | S | R-X-X-pS-P  pS-P-X-X-X-X-R  pS-P-X-R  pS-P-X-X-K  pS-D-D-E  **pS-P-X-X-X-X-X-X-X-R**  pS-D-E-E  pS-P  R-R-X-pS  pS-E-X-E-X-D  pS-E-X-E  R-X-X-pS  pS-D-X-E  **pS-R-S***  pS-D-X-D  R-X-X-S-X-pS  **pS-X-X-X-X-D-E***  R-X-pS  **D-E-X-X-X-X-X-X-X-pS***  **D-D-X-X-X-X-X-pS**  R-X-X-X-X-X-X-X-pS  pS-X-X-X-X-R-X-X-S  pS-X-D  pS-X-X-X-X-X-X-X-X-X-R  **E-E-X-X-X-X-X-X-X-pS**  **pS-X-X-X-X-X-R-X-X-S**  pS-X-E  pS-X-X-R  R-pS  R-X-X-X-X-X-X-X-X-pS  pS-X-X-X-R  R-X-X-X-X-X-X-X-X-X-pS  D-X-X-X-X-X-X-pS  R-X-X-X-X-X-X-pS  R-X-X-X-pS  pS-X-X-X-X-X-X-R  **E-X-pS***  R-X-X-X-X-X-pS  pS-X-X-X-X-R  **pS-X-R**  **K-X-X-X-X-pS***  **pS-X-P***  pS-X-X-X-X-E | 28.88  25.51  23.29  22.13  43.35  22.36  46.21  16.00  26.74  39.88  30.91  16.00  32.00  22.56  32.00  23.74  22.91  16.00  22.00  26.94  14.68  22.40  14.00  12.85  17.79  17.79  10.30  10.84  11.15  10.51  10.90  10.57  9.88  9.75  8.87  8.34  7.61  7.24  8.03  8.44  8.08  7.68  7.84 |
|  | T | pT-P-P  pT-P | 26.36  16.00 |
|  | Y | No motif |  |

**Table A.** Sequence motifs of all phosphorylation sites at 70% sequence similarity with the p < 0.000001 significance threshold. The motif score represents the sum of the negative log probabilities used to fix each position of the motif. The higher the motif score, the more statistically significant the corresponding motif is.

| AA/Pos | -6 | -5 | -4 | -3 | -2 | -1 | 1 | 2 | 3 | 4 | 5 | 6 |
| --- | --- | --- | --- | --- | --- | --- | --- | --- | --- | --- | --- | --- |
| G | n/n | n/n | n/n | n/+ | -/n | +/+ | -/n | n/n | n/n | n/n | n/n | n/n |
| S | +/+ | +/n | +/+ | +/+ | +/+ | +/+ | n/+ | +/+ | +/+ | +/+ | n/+ | n/n |
| T | n/n | +/n | +/n | +/+ | +/n | +/n | -/n | n/n | -/n | n/n | n/n | -/n |
| Y | n/+ | +/+ | +/n | +/+ | +/n | +/n | -/n | -/- | -/n | -/n | n/- | -/n |
| C | -/n | -/n | -/- | -/- | -/n | -/n | -/n | -/n | -/n | -/n | -/- | -/- |
| N | n/n | n/n | n/n | -/- | n/- | n/- | -/- | n/- | n/n | n/n | n/n | n/n |
| Q | -/n | n/n | n/n | -/n | n/n | -/n | -/- | -/- | -/- | -/n | n/- | -/- |
| K | +/n | +/n | +/- | +/n | n/- | n/n | -/- | -/- | n/- | -/- | n/n | +/n |
| R | +/n | +/n | +/n | +/- | +/- | +/n | -/- | -/- | n/- | n/- | n/- | +/+ |
| H | n/n | n/n | -/- | -/- | -/n | -/- | -/n | -/- | -/n | -/n | -/- | -/- |
| D | n/+ | n/+ | n/+ | +/+ | +/+ | +/+ | +/+ | +/+ | +/+ | +/+ | +/+ | +/+ |
| E | +/+ | +/n | +/n | n/+ | n/+ | n/+ | +/n | +/+ | +/+ | +/+ | +/+ | +/+ |
| P | n/n | n/n | n/- | -/n | n/n | -/n | +/+ | +/+ | n/- | n/n | +/+ | n/n |
| A | n/n | n/+ | n/+ | -/n | n/n | n/n | -/- | -/n | n/n | n/n | n/n | n/n |
| W | -/- | n/n | n/n | -/n | n/n | n/n | n/n | -/n | -/n | n/n | -/n | n/n |
| F | -/n | -/n | -/n | -/n | -/n | -/n | n/- | -/- | n/- | -/- | -/n | n/n |
| L | -/n | -/- | -/n | -/- | -/n | n/- | -/- | -/- | -/- | +/- | -/- | -/- |
| I | n/- | n/n | -/- | -/- | -/n | -/- | -/- | -/- | -/- | n/n | -/n | -/- |
| M | n/n | n/n | -/- | n/n | n/n | n/n | -/- | -/n | n/n | n/n | n/n | n/n |
| V | -/- | -/- | -/- | -/- | -/n | -/- | -/- | -/- | -/- | n/- | -/- | -/- |

**Table B.** Two sample logo comparison of phosphorylation sites between our study and the study of Lundby *et al* in brain. “+” and “-” represent enrichment and depletion of amino acids at a particular position, respectively, whereas “n” represents the lack of depletion or enrichment. Numerator of each fraction in each cell corresponds to the finding in our study, whereas denominator corresponds to the observation in the study of Lundby *et al*. 58.8% of the cases overlap in both studies (n/n, +/+, -/-). In 27.5% of the cases we found a particular amino acid enriched/depleted in the corresponding position, and the study by Lundby et al. found no signal, whereas in 12.1% of the cases it is *vice versa*. All discordant cases (n/+, n/-, +/n, -/n, +/-, -/+) are highlighted with yellow.

| AA/Pos | -6 | -5 | -4 | -3 | -2 | -1 | 1 | 2 | 3 | 4 | 5 | 6 |
| --- | --- | --- | --- | --- | --- | --- | --- | --- | --- | --- | --- | --- |
| G | n/n | -/- | n/n | n/+ | n/n | +/n | -/n | n/n | n/- | n/n | n/- | n/n |
| S | n/n | n/n | n/n | n/n | +/+ | +/n | -/+ | +/+ | n/n | n/n | -/n | n/- |
| T | +/n | n/n | +/n | +/n | +/n | +/+ | -/n | n/n | -/- | -/- | n/n | -/- |
| Y | n/n | +/n | +/n | n/- | n/n | +/n | -/- | -/n | -/n | -/n | n/n | n/n |
| C | -/- | -/n | -/n | -/n | -/n | -/n | -/n | -/n | -/n | -/n | -/n | -/n |
| N | -/n | -/n | n/n | -/n | n/n | n/n | -/n | n/n | n/n | n/n | n/n | n/n |
| Q | n/n | n/n | -/- | n/n | +/n | -/- | -/n | -/- | -/n | -/- | n/n | n/n |
| K | +/+ | n/n | +/n | n/- | -/- | n/- | -/- | -/n | n/- | -/n | n/n | +/+ |
| R | +/n | +/+ | +/+ | +/- | +/- | +/n | -/- | -/- | n/- | n/- | +/n | +/n |
| H | -/n | n/n | -/n | n/n | -/n | -/n | -/n | -/n | -/- | -/n | n/n | n/n |
| D | +/+ | n/+ | n/n | n/+ | n/+ | +/+ | +/+ | +/+ | +/+ | +/+ | +/+ | +/+ |
| E | n/+ | n/+ | n/+ | n/+ | n/+ | n/+ | +/+ | +/+ | +/+ | +/+ | +/+ | +/+ |
| P | +/n | n/n | n/n | n/n | n/n | -/n | +/n | +/n | +/+ | n/n | +/n | n/n |
| A | n/n | n/n | n/n | n/n | n/- | n/n | -/n | -/- | -/- | -/- | -/n | n/n |
| W | n/n | -/n | n/n | n/n | n/n | n/n | -/- | -/n | n/n | n/n | -/n | n/n |
| F | -/- | n/- | n/n | -/n | n/n | n/- | n/- | -/- | n/n | n/n | n/n | n/n |
| L | -/- | n/n | -/n | -/n | -/- | n/n | -/- | -/- | -/n | +/+ | n/- | -/- |
| I | -/- | n/n | n/n | -/n | -/- | -/- | -/- | -/- | -/n | +/n | -/n | -/n |
| M | n/n | n/- | n/n | n/n | n/- | n/n | n/- | -/n | n/- | n/n | n/n | n/n |
| V | -/n | -/- | n/n | -/n | n/n | -/- | -/- | n/n | -/- | n/- | -/n | -/- |

**Table C.** Two sample logo comparison of phosphorylation sites between our study and the study of Lundby *et al* in testis. “+” and “-” represent enrichment and depletion of amino acids at a particular position, respectively, whereas “n” represents the lack of depletion or enrichment. Numerator of each fraction in each cell corresponds to the finding in our study, whereas denominator corresponds to the observation in the study of Lundby *et al*. 57.5% of the cases overlap in both studies (n/n, +/+, -/-). In 29.2% of the cases we found a particular amino acid enriched/depleted in the corresponding position, and the study by Lundby et al. found no signal, whereas in 12.1% of the cases it is *vice versa*. All discordant cases (n/+, n/-, +/n, -/n, +/-, -/+) are highlighted with yellow.

| Tissue | Number of PSS | Number of non-PSS | Solvent accessibility | B-factor scores |
| --- | --- | --- | --- | --- |
| Global | 423 | 4162 | **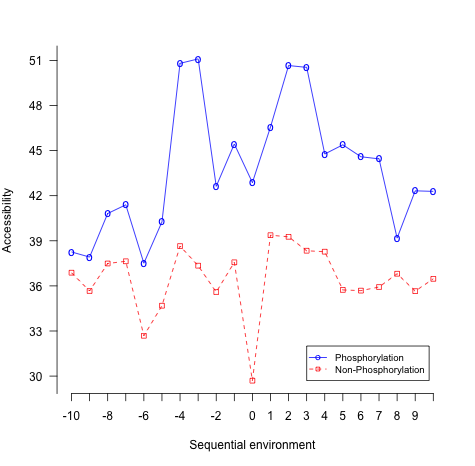** | **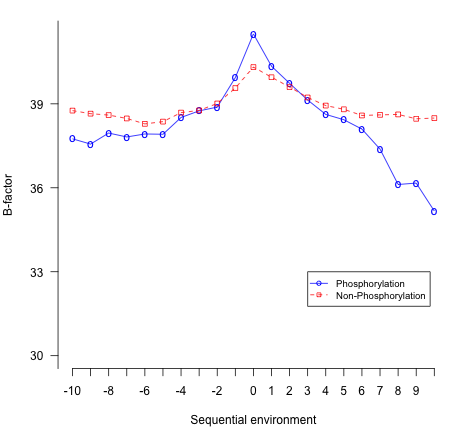** |
| Blood | 38 | 625 | **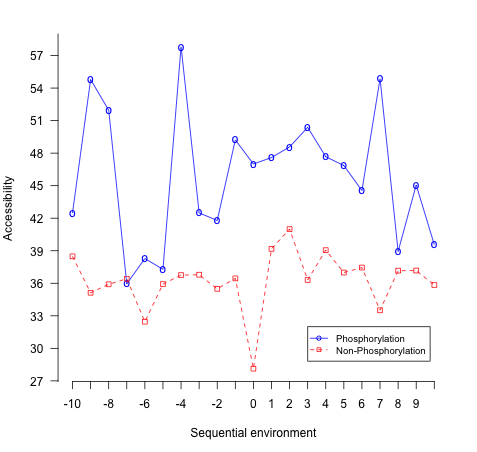** | **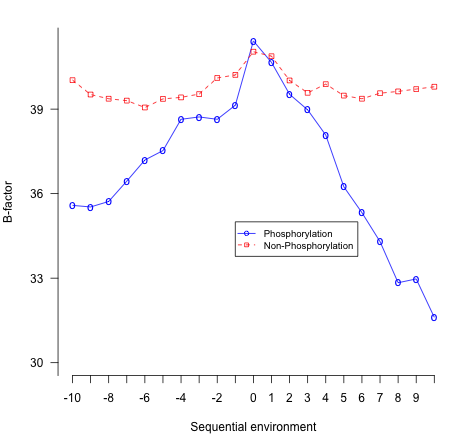** |
| Brain | 122 | 1758 | **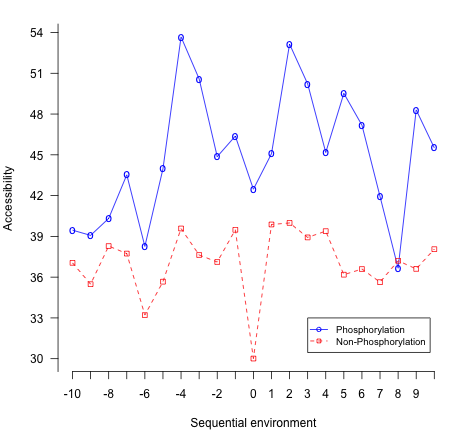** | **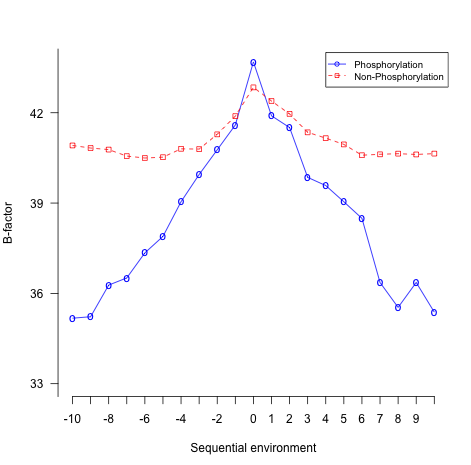** |
| Brainstem | 81 | 1318 | **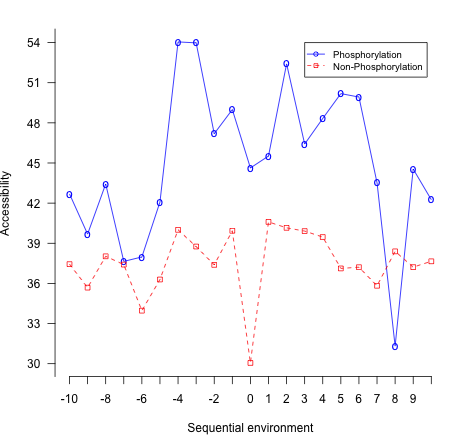** | **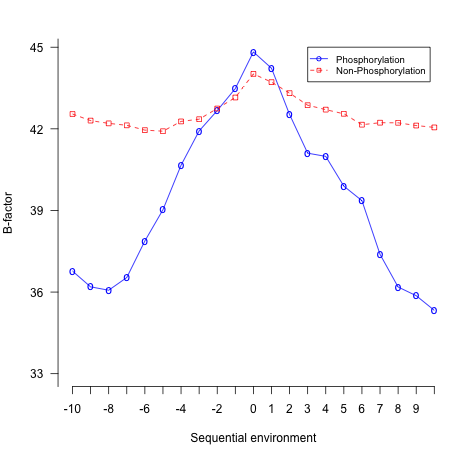** |
| Cerebellum | 82 | 1411 | **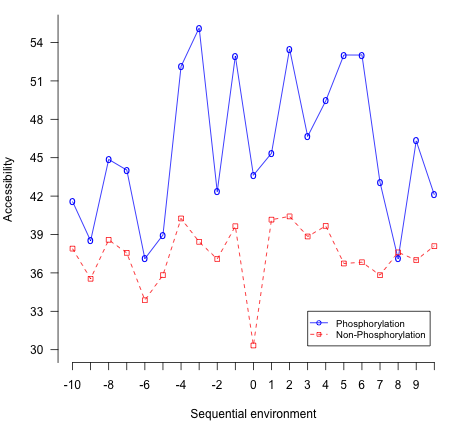** | **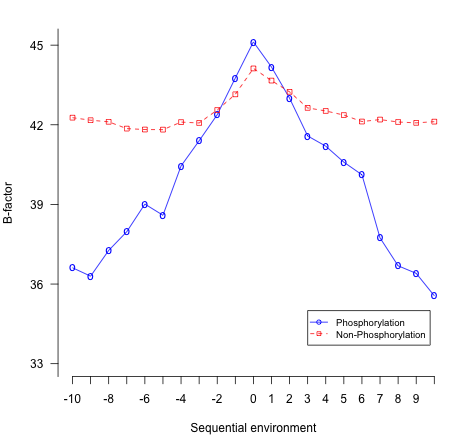** |
| Cortex | 93 | 1483 | **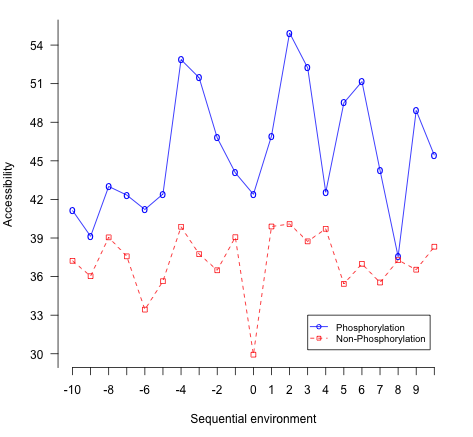** | **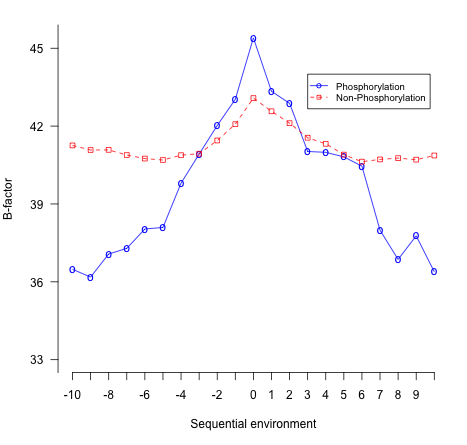** |
| Heart | 44 | 668 | **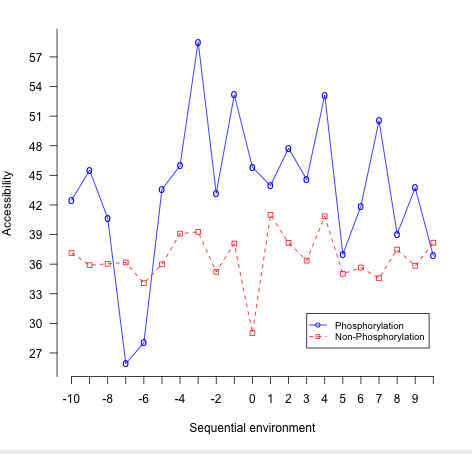** | **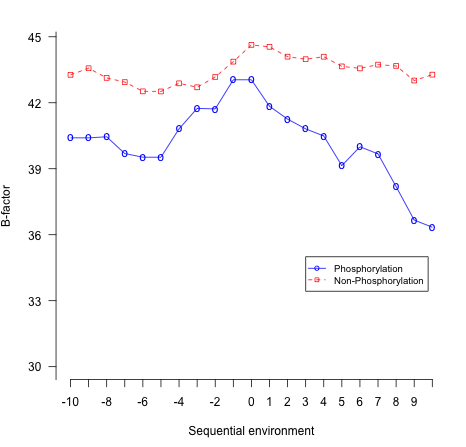** |
| Intestine | 69 | 1139 | **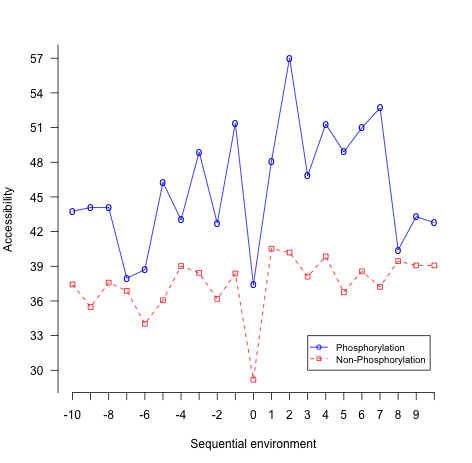** | **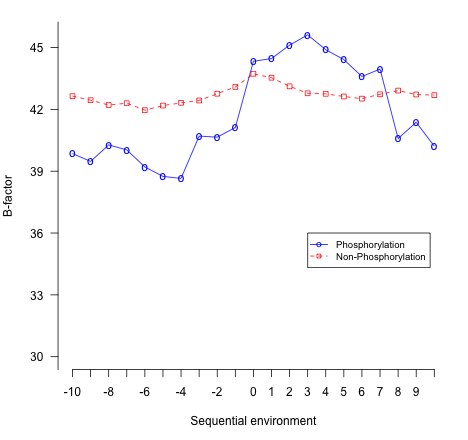** |
| Kidney | 66 | 1062 | **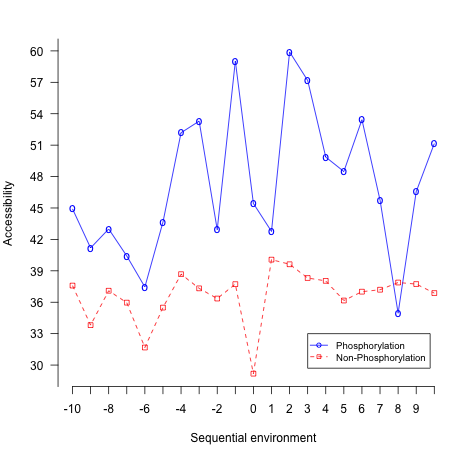** | **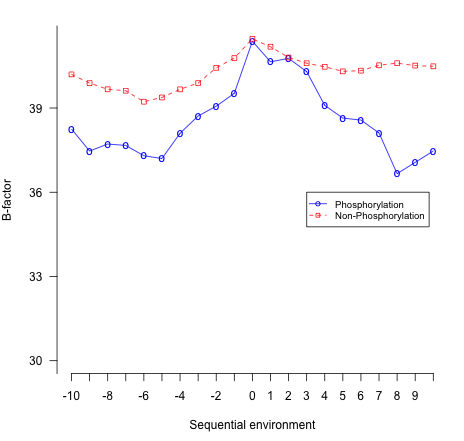** |
| Liver | 97 | 1305 | **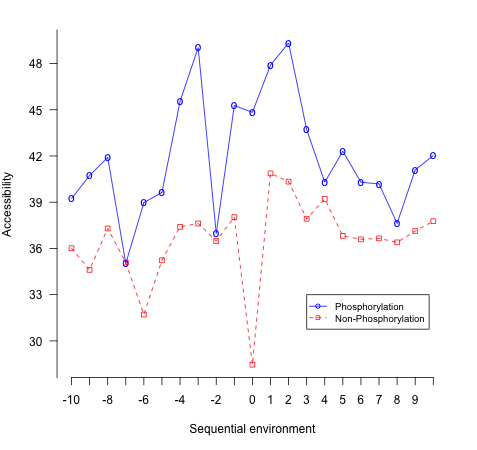** | **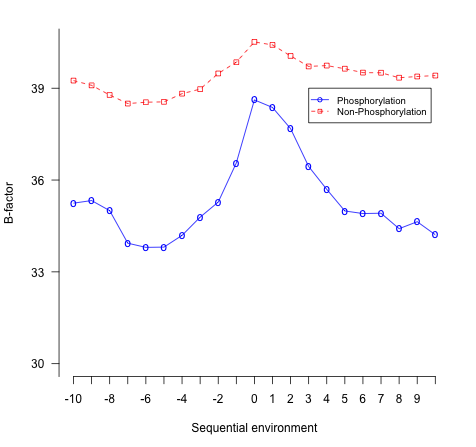** |
| Lung | 73 | 1216 | **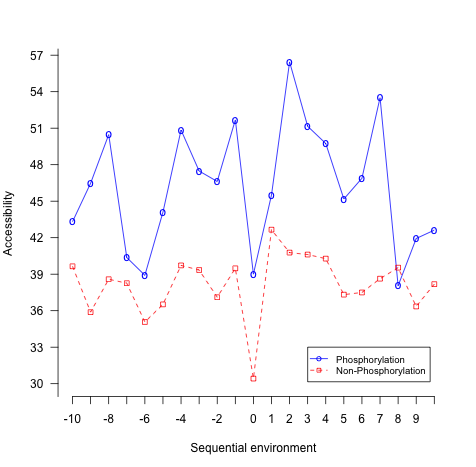** | **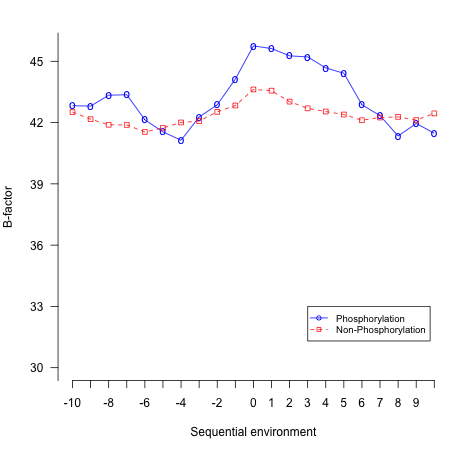** |
| Muscle | 95 | 889 | **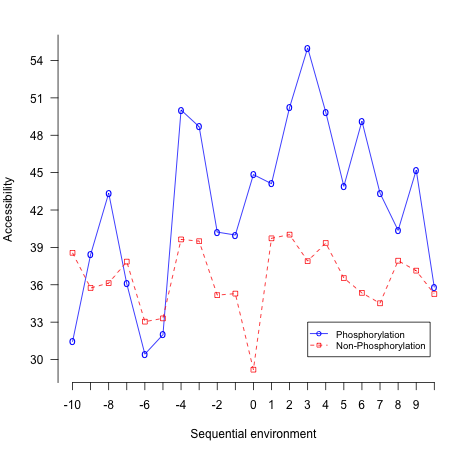** | **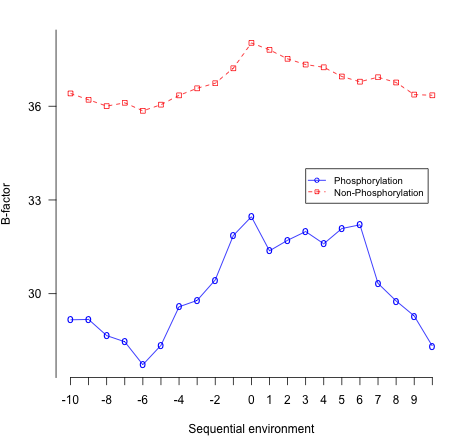** |
| Pancreas | 10 | 349 | **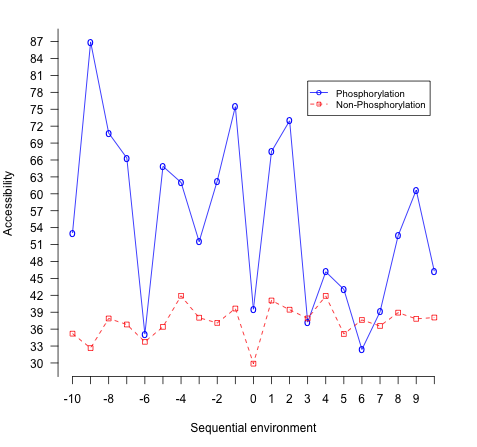** | **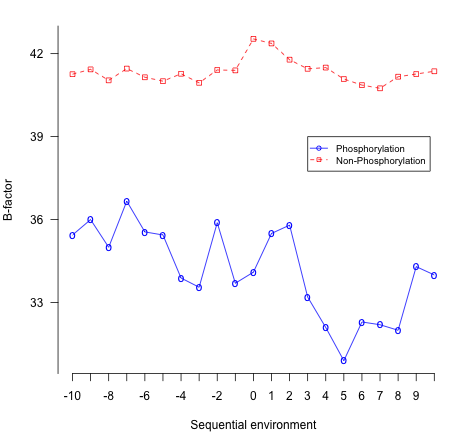** |
| Perirenal fat | 56 | 850 | **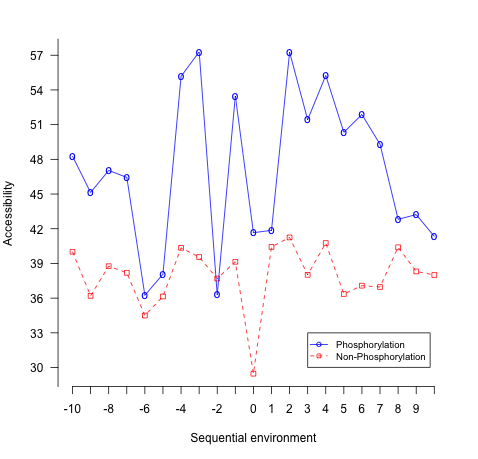** | **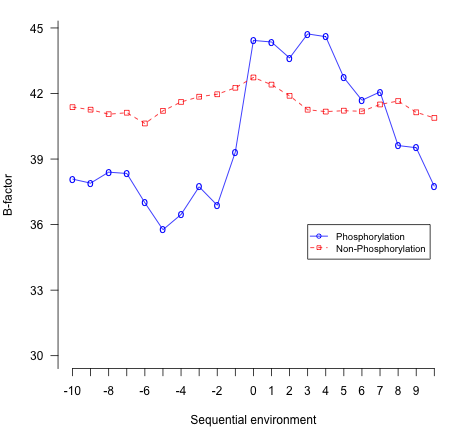** |
| Spleen | 73 | 1330 | **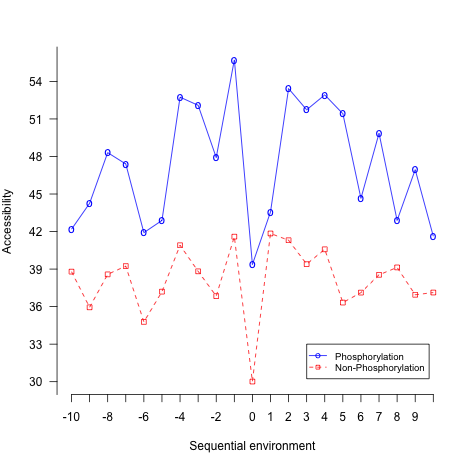** | **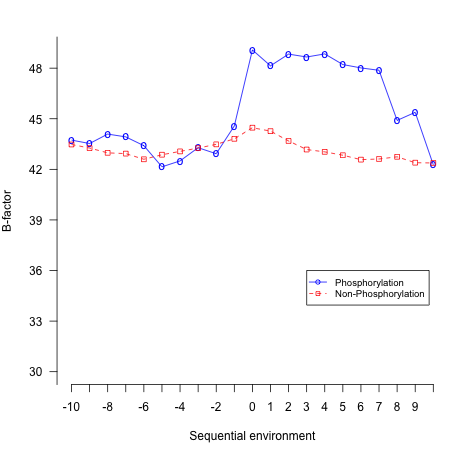** |
| Stomach | 66 | 1107 | **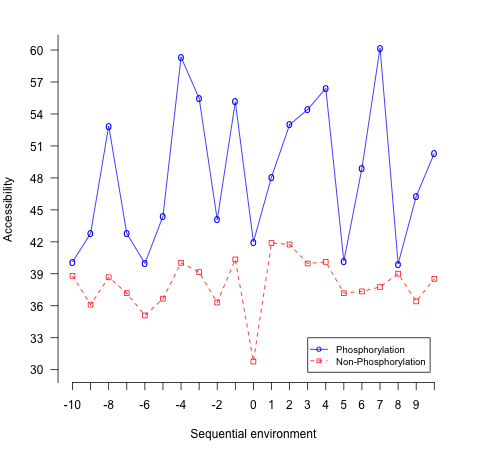** | **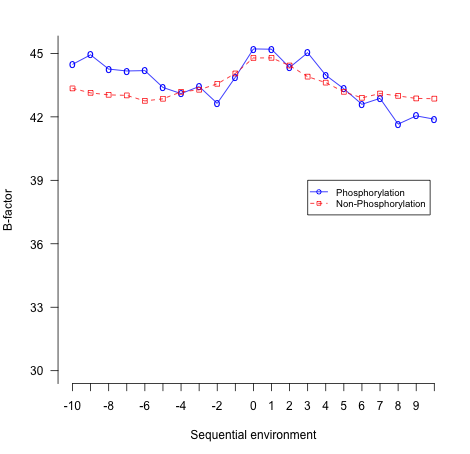** |
| Testis | 45 | 922 | **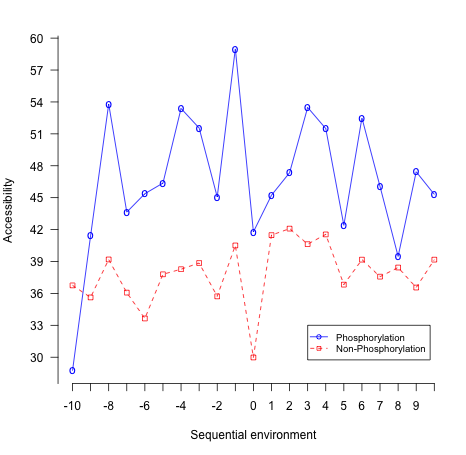** | **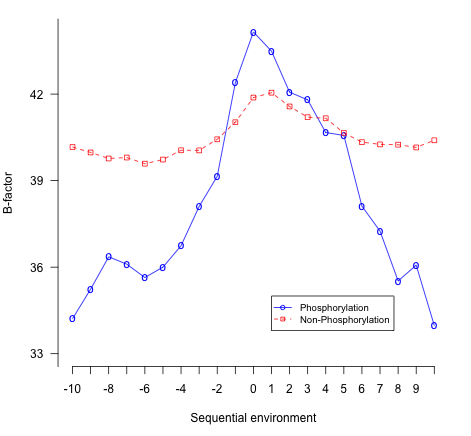** |
| Thymus | 65 | 1154 | **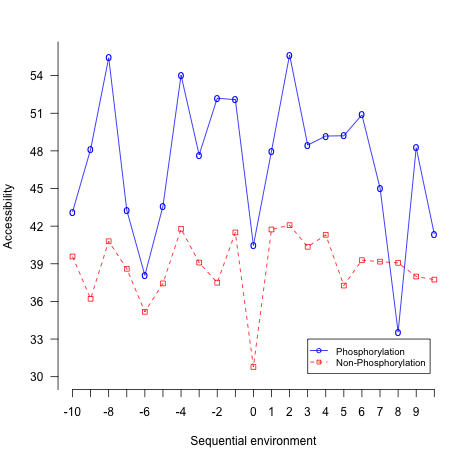** | **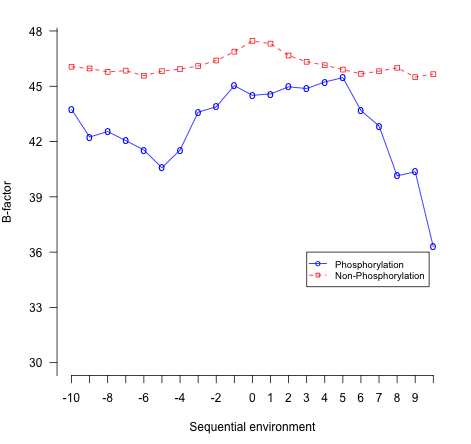** |

**Table D.** Accessibility and B-factor analysis of PSS in different tissues in the PS3D-90 dataset.

| Tissue | Number of PTS | Number of non-PTS | Solvent accessibility | B-factor scores |
| --- | --- | --- | --- | --- |
| Global | 140 | 3790 | **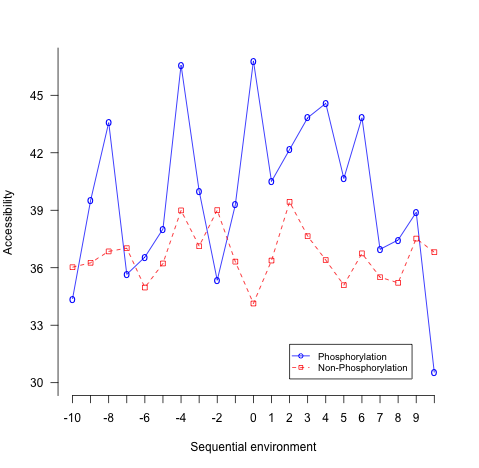** | **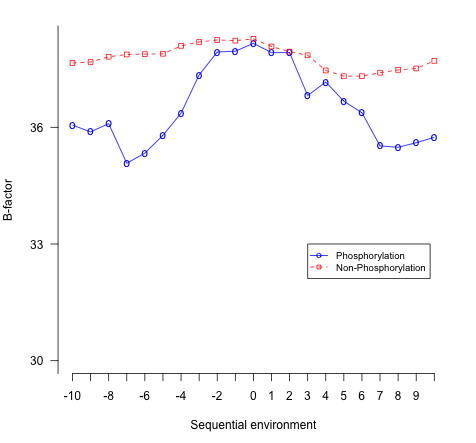** |
| Blood | 6 | 567 | **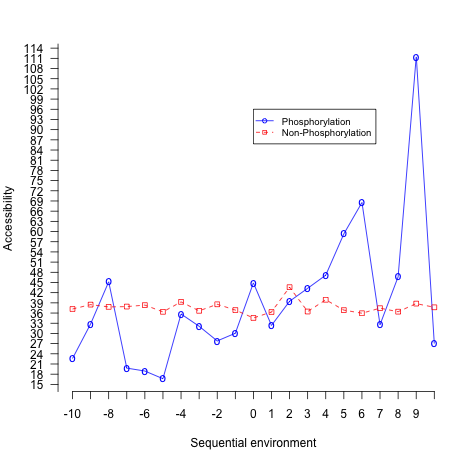** | **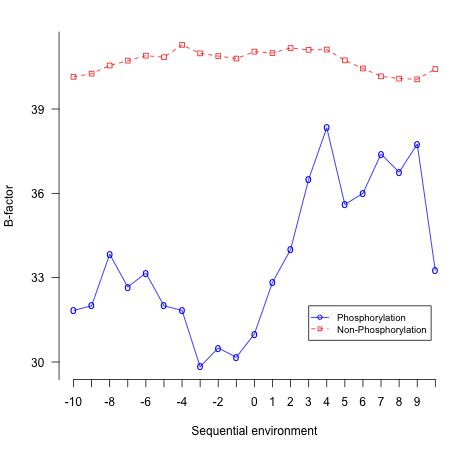** |
| Brain | 26 | 1597 | **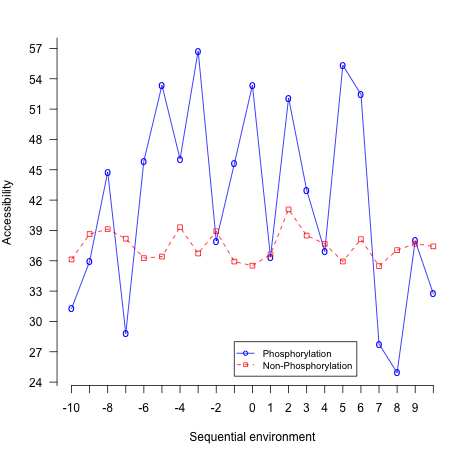** | **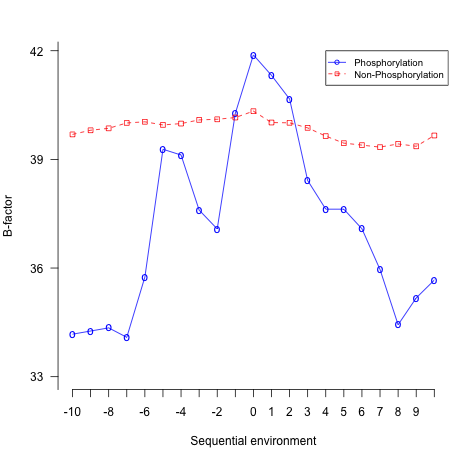** |
| Brainstem | 13 | 1223 | **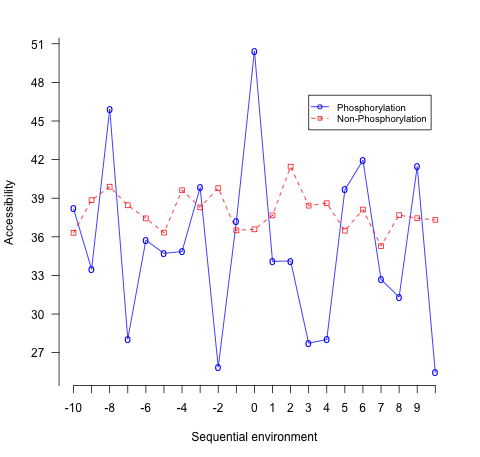** | **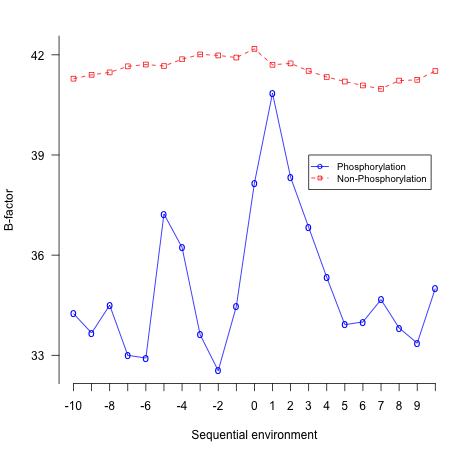** |
| Cerebellum | 16 | 1281 | **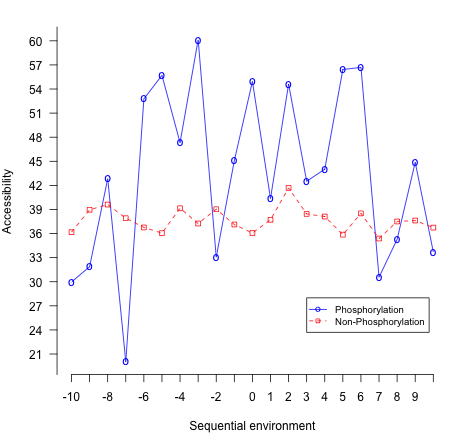** | **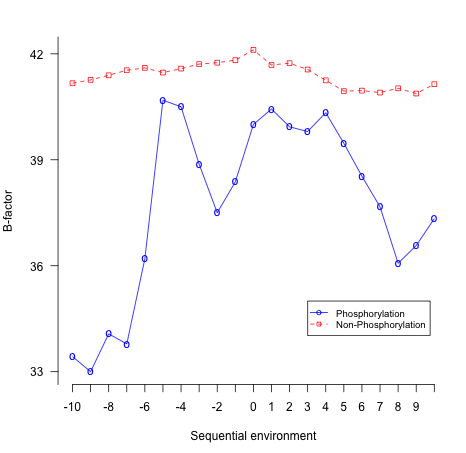** |
| Cortex | 17 | 1399 | **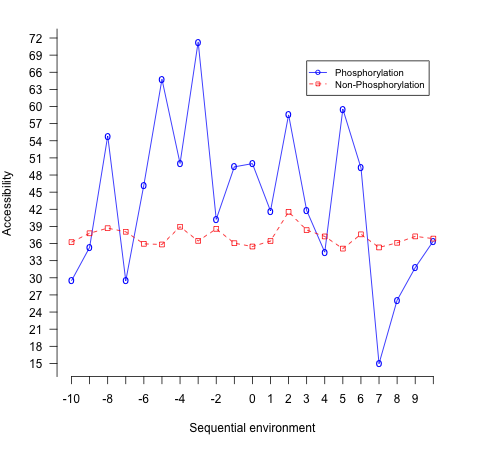** | **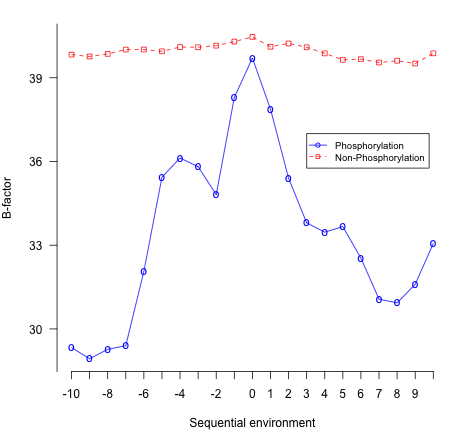** |
| Heart | 10 | 652 | **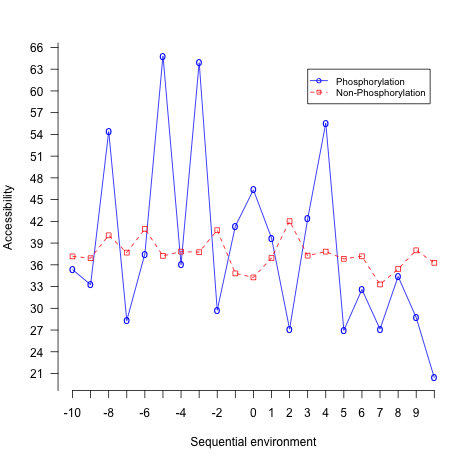** | **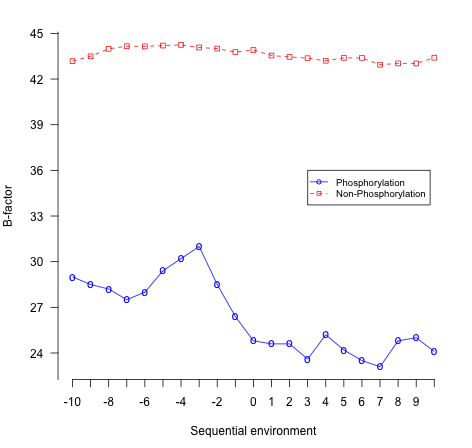** |
| Intestine | 10 | 1146 | **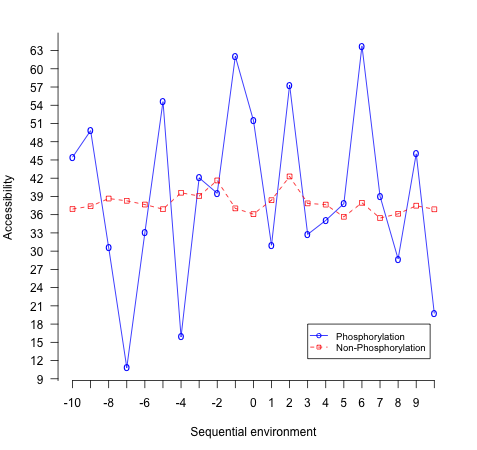** | **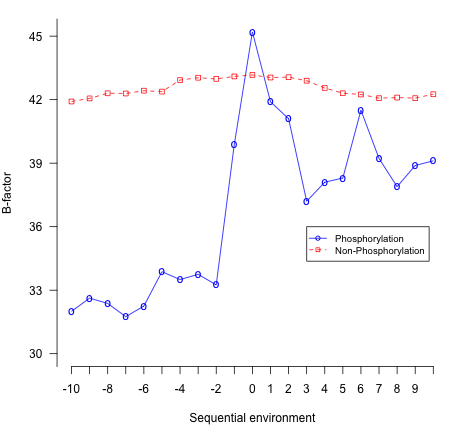** |
| Kidney | 9 | 1002 | **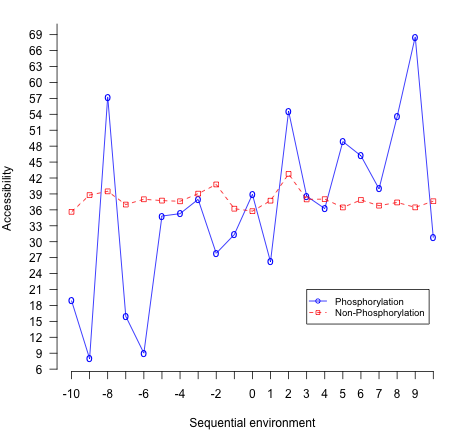** | **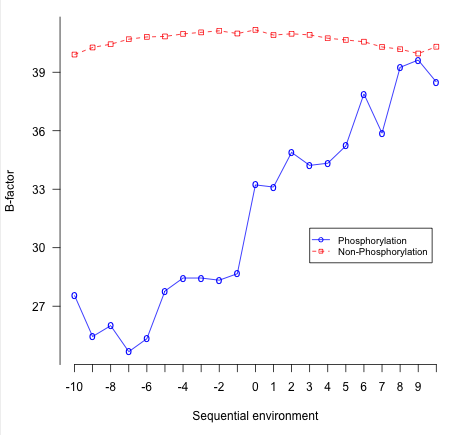** |
| Liver | 22 | 1179 | **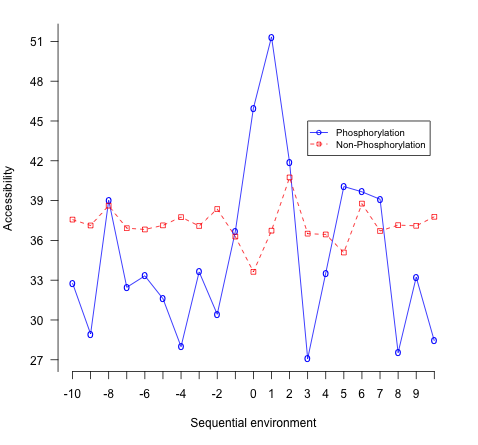** | **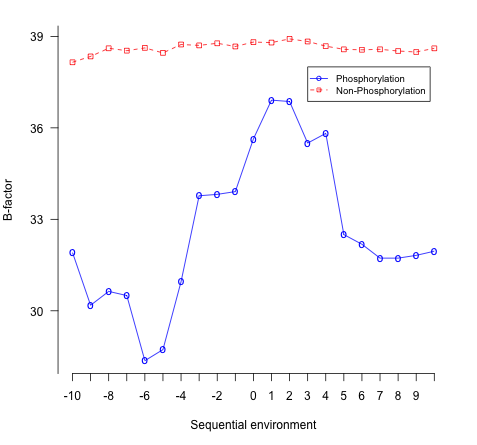** |
| Lung | 6 | 1176 | **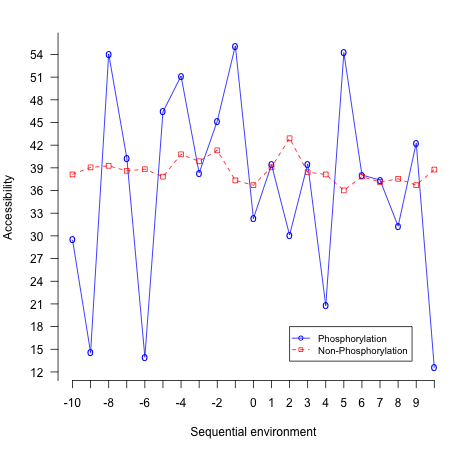** | **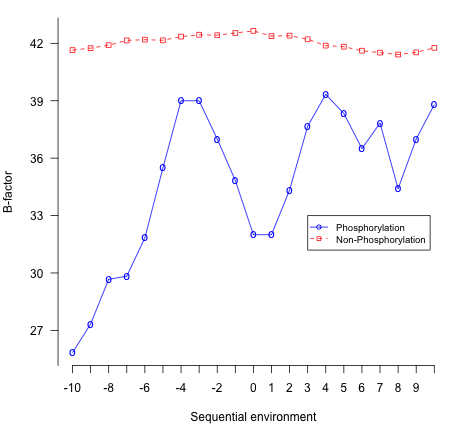** |
| Muscle | 51 | 847 | **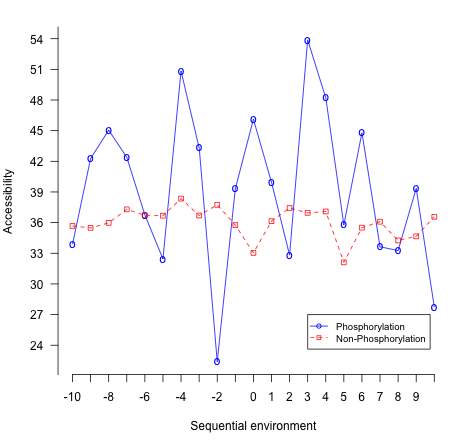** | **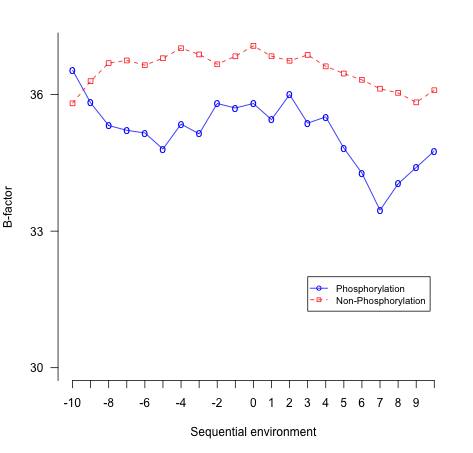** |
| Pancreas | 3 | 319 | **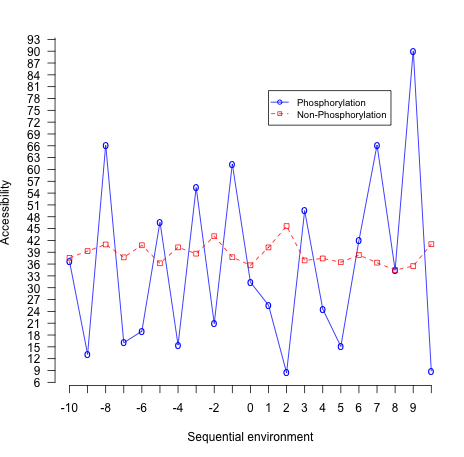** | **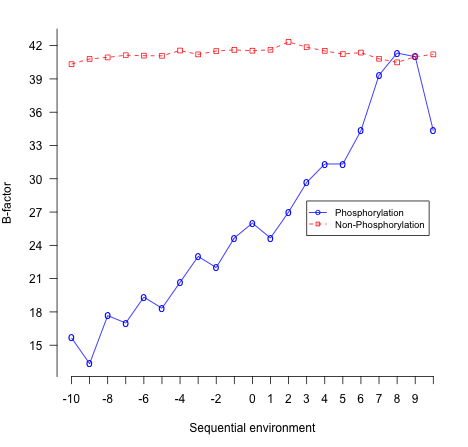** |
| Perirenal fat | 8 | 796 | **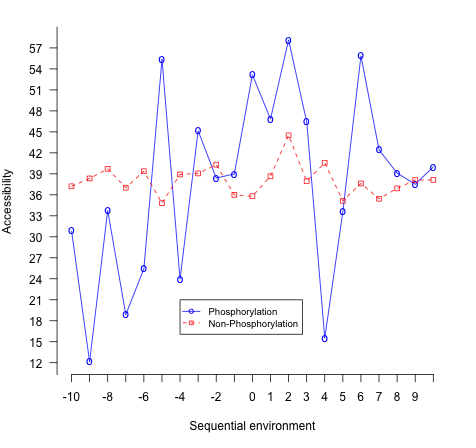** | **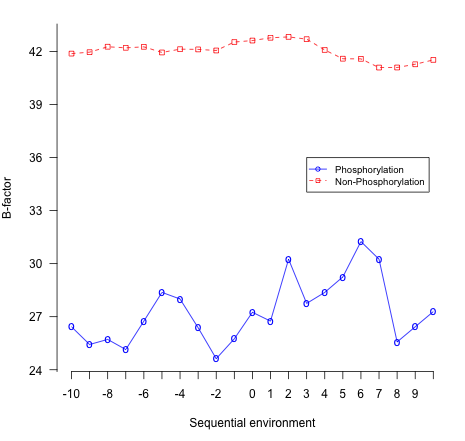** |
| Spleen | 7 | 1280 | **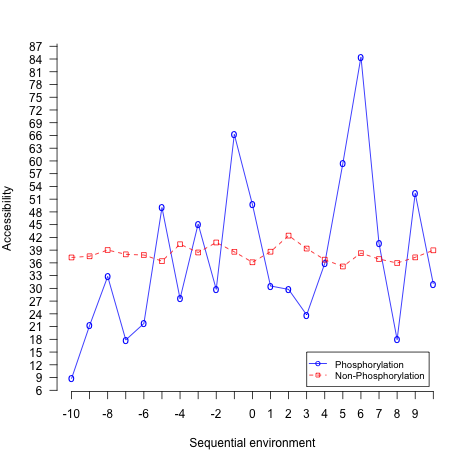** | **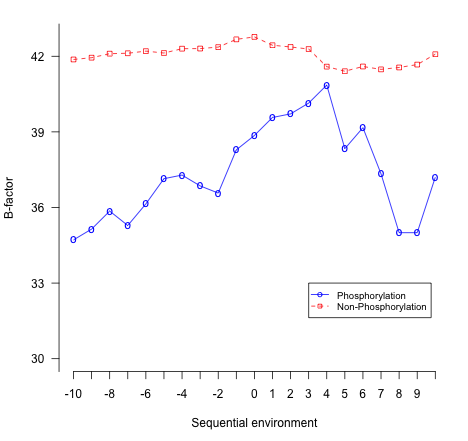** |
| Stomach | 13 | 1128 | **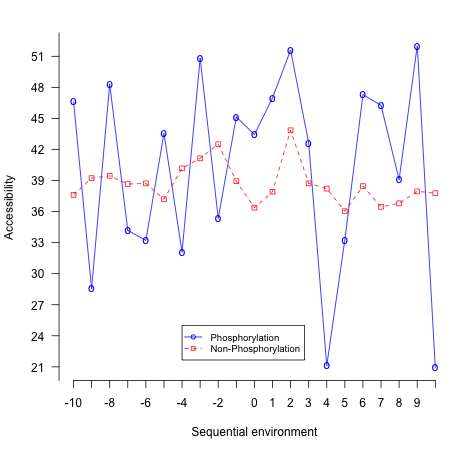** | **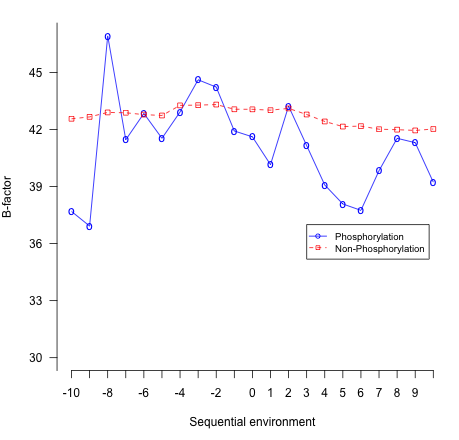** |
| Testis | 4 | 848 | **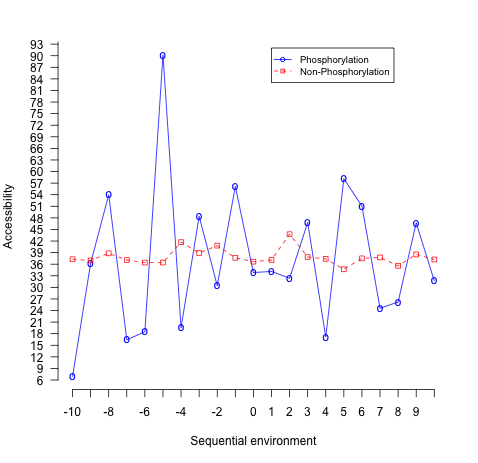** | **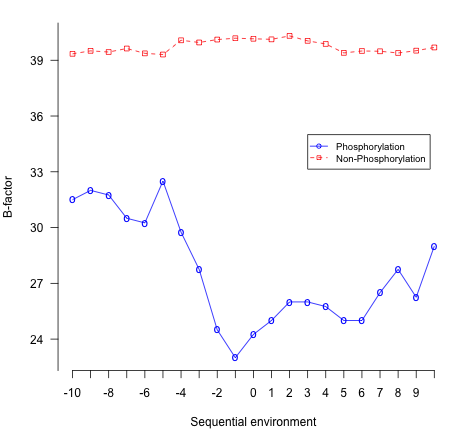** |
| Thymus | 5 | 1110 | **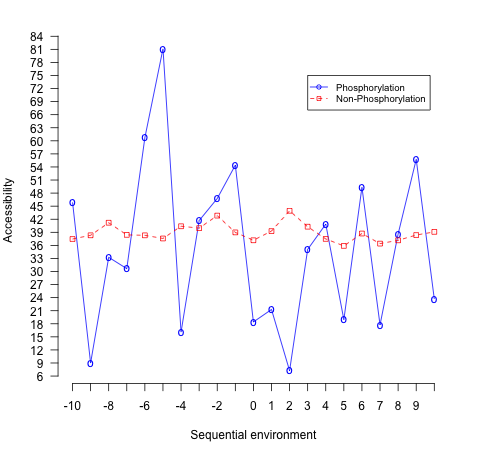** | **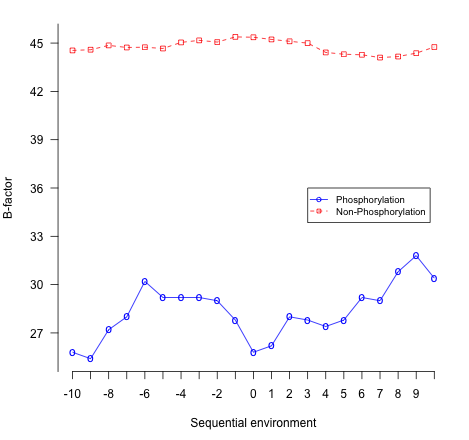** |

**Table E.** Accessibility and B-factor analysis of PTS in different tissues in the PS3D-90 dataset.

| Tissue | Number of PYS | Number of non-PYS | Solvent accessibility | B-factor scores |
| --- | --- | --- | --- | --- |
| Global | 46 | 2804 | **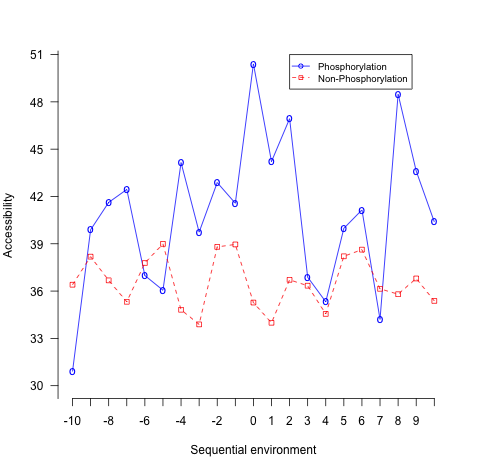** | **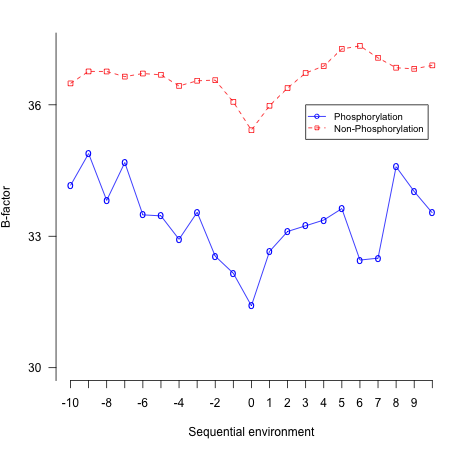** |
| Blood | 2 | 397 | **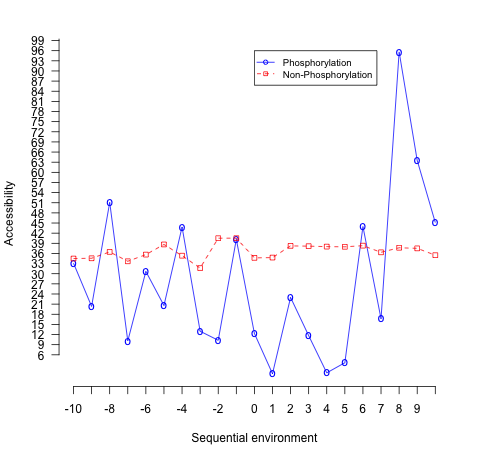** | **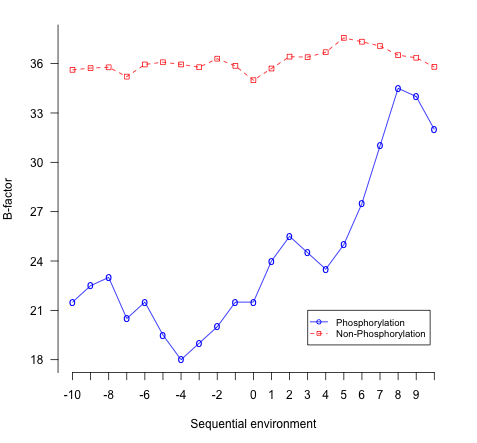** |
| Brain | 4 | 1159 | **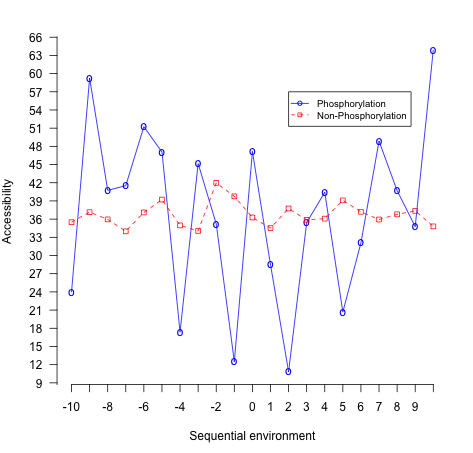** | **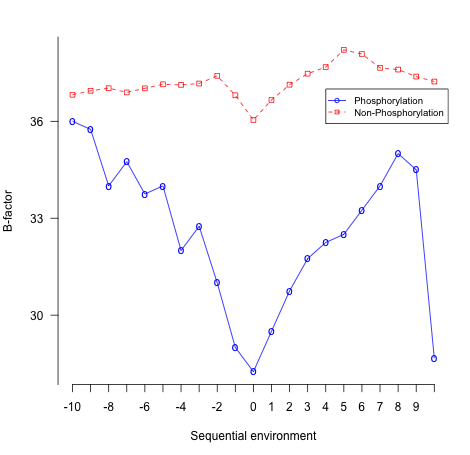** |
| Brainstem | 2 | 829 | **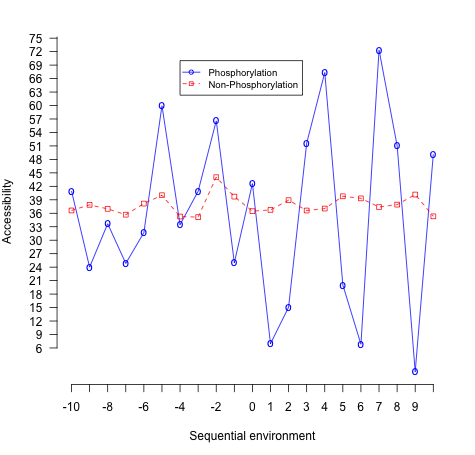** | **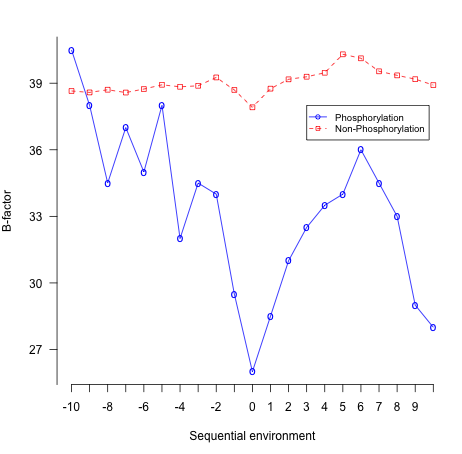** |
| Cerebellum | 3 | 906 | **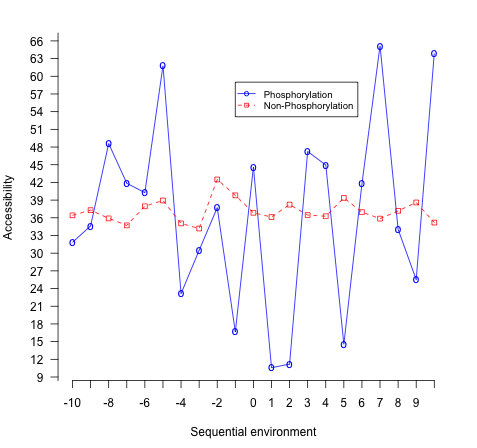** | **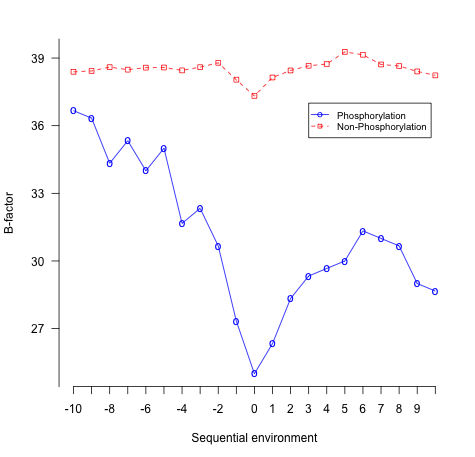** |
| Cortex | 3 | 974 | **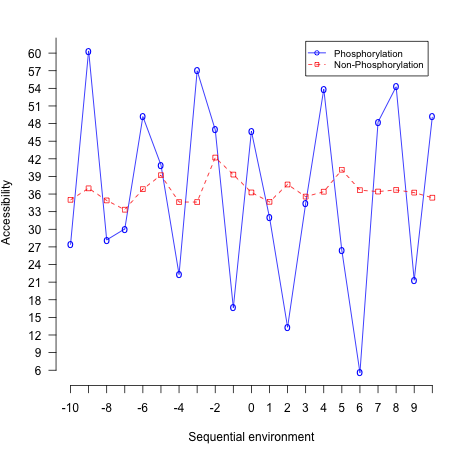** | **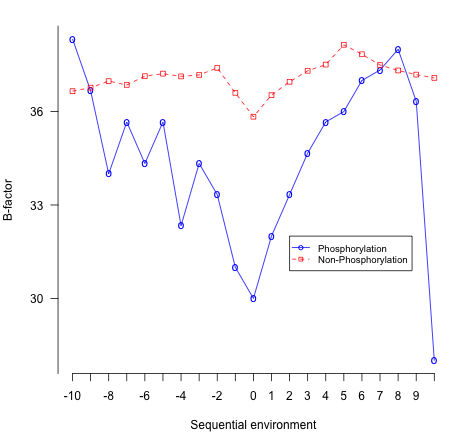** |
| Heart | 5 | 452 | **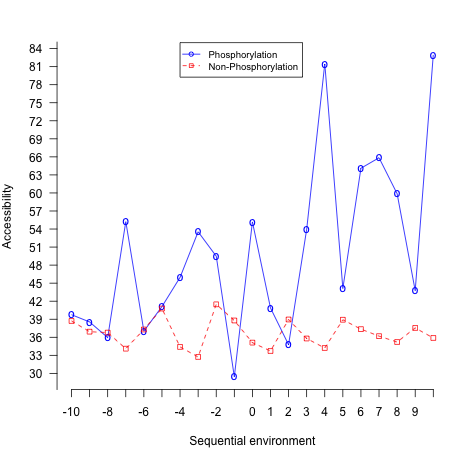** | **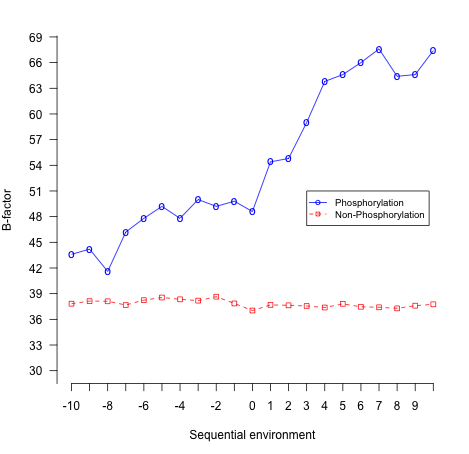** |
| Intestine | 3 | 788 | **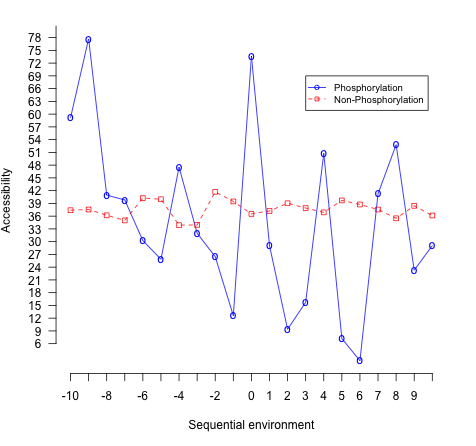** | **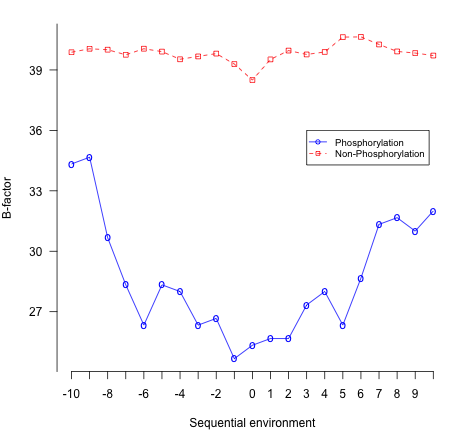** |
| Kidney | 2 | 731 | **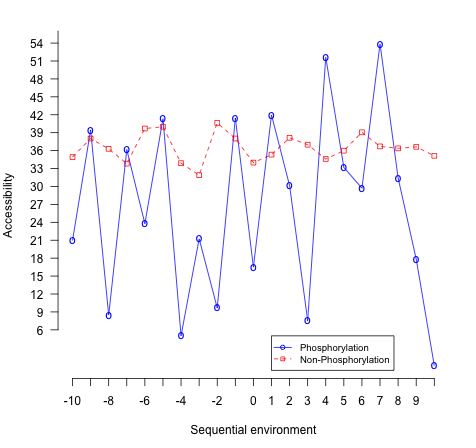** | **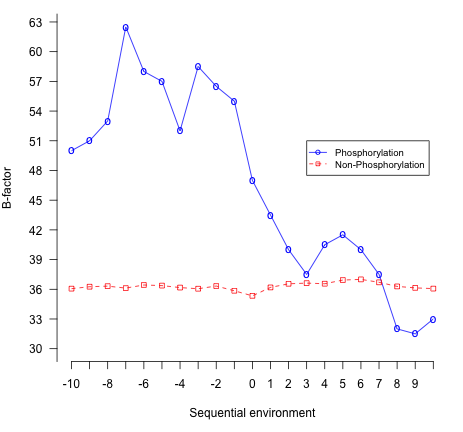** |
| Liver | 3 | 918 | **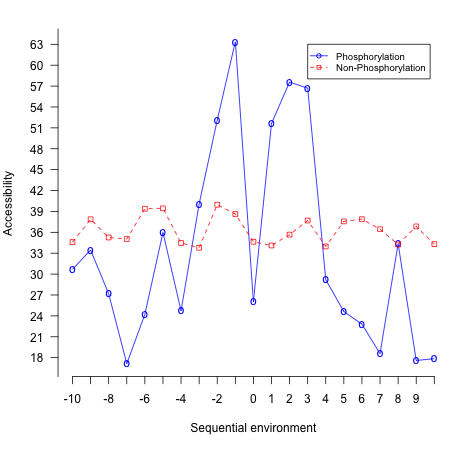** | **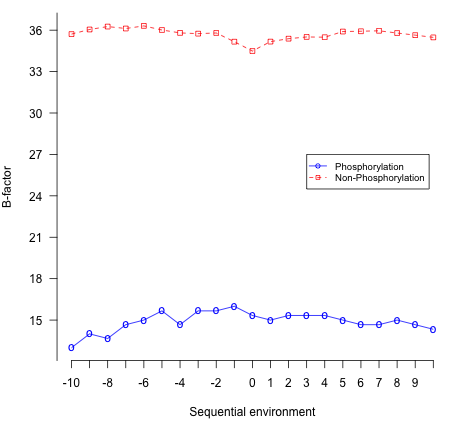** |
| Lung | 6 | 834 | **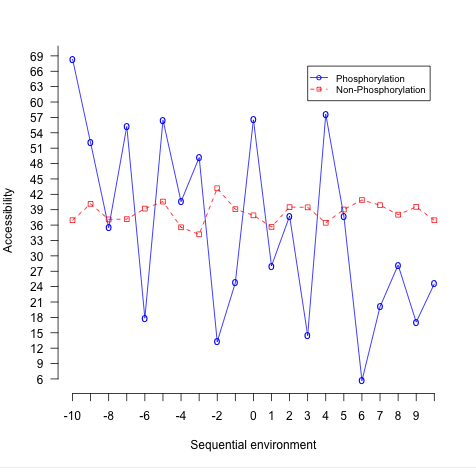** | **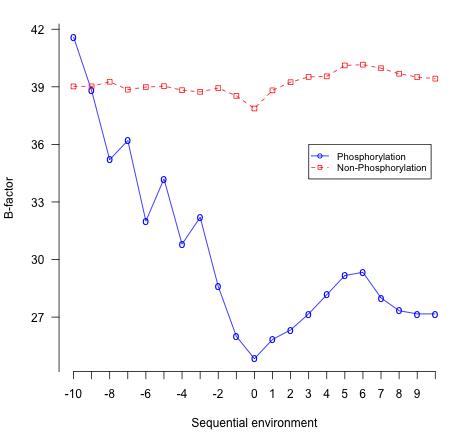** |
| Muscle | 27 | 602 | **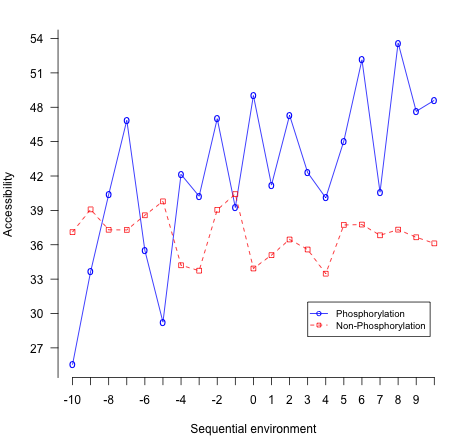** | **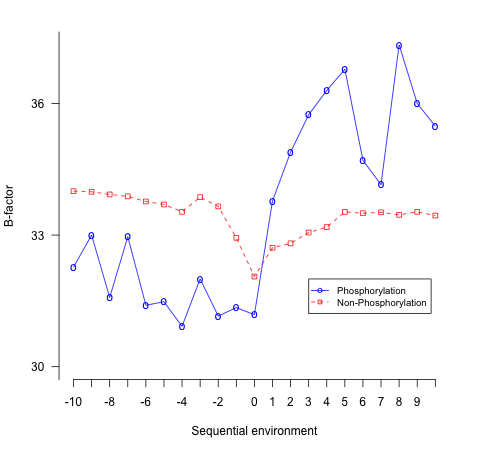** |
| Pancreas | 0 | 276 | Not applicable | Not applicable |
| Perirenal fat | 3 | 557 | **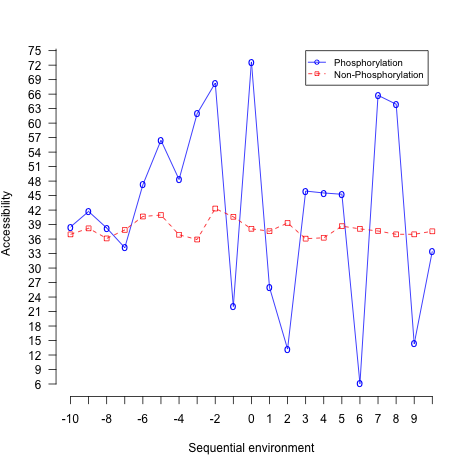** | **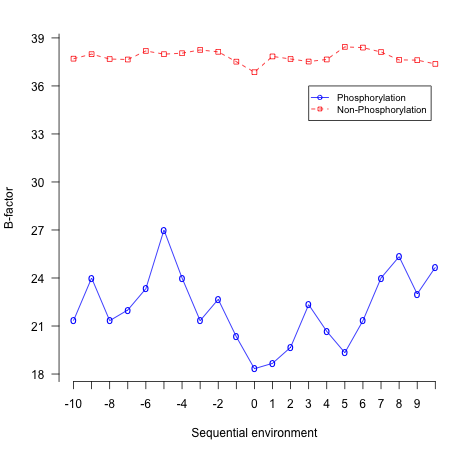** |
| Spleen | 4 | 949 | **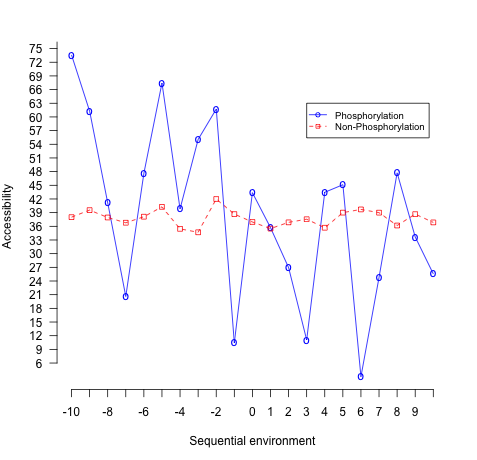** | **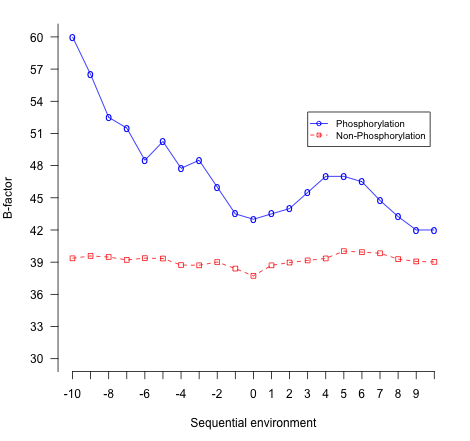** |
| Stomach | 2 | 727 |  |  |
| Testis | 1 | 661 |  |  |
| Thymus | 2 | 826 |  |  |

**Table F.** Accessibility and B-factor analysis of PYS in different tissues in the PS3D-90 dataset.
